# Graphite lubricates Mercury’s global contraction

**DOI:** 10.1038/s41467-026-75459-x

**Published:** 2026-08-26

**Authors:** Natalia A. VERGARA SASSARINI, Matteo MASSIRONI, Telemaco TESEI, Andrea BISTACCHI

**Affiliations:** 1 INAF, Osservatorio Astronomico di Padova, Vicolo dell’Osservatorio 5, 35122 Padova, Italy; 2 CISAS, Università degli Studi di Padova, 35131 Padova, Italy; 3 Dipartimento di Geoscienze, Università degli Studi di Padova, 35131 Padova, Italy; 4 Dipartimento di Scienze dell’Ambiente e della Terra, Università degli Studi di Milano - Bicocca, Milano, Italy

**Keywords:** Tectonics, Structural geology, Inner planets

## Abstract

Secular cooling of Mercury’s interior drove planetary contraction, forming widespread lobate scarps that are the surface expressions of thrust faults and fold-and-thrust belts. Despite their importance to Mercury’s global tectonics, the mechanics and rheology of these features remain poorly understood. Using Critical Taper analysis calibrated by friction experiments, we estimate the maximum basal friction (µ_b_) from observed wedge geometry and show that the topography of large fold-and-thrust belts is consistent with weak graphite-bearing, low-angle fault zones. This finding indirectly supports models proposing a graphite flotation crust during Mercury’s early differentiation. We infer that remnants of this carbon-rich layer, heterogeneously distributed within Mercury’s crust because of early impacts, caused the weakening of large portions of the crust during later contraction, allowing the nucleation of weak, low-angle thrust faults. Since the gently dipping, frictionally weak thrusts require greater horizontal shortening to build the observed relief than steeper, stronger faults, Mercury’s cumulative radial contraction may exceed previous, more conservative estimates.

## Abstract (web summary)

Laboratory experiments and mechanical modeling show that Mercury’s widespread low-angle thrust faults likely formed due to graphite-rich layers in its crust, which acted as a solid lubricant and weakened fault zones during planetary contraction. These findings suggest that remnants of an early graphite flotation crust facilitated the development of weak, shallow-dipping faults, indicating Mercury’s total radial contraction may be greater than previously estimated.

## Introduction

**Fig. 1:**
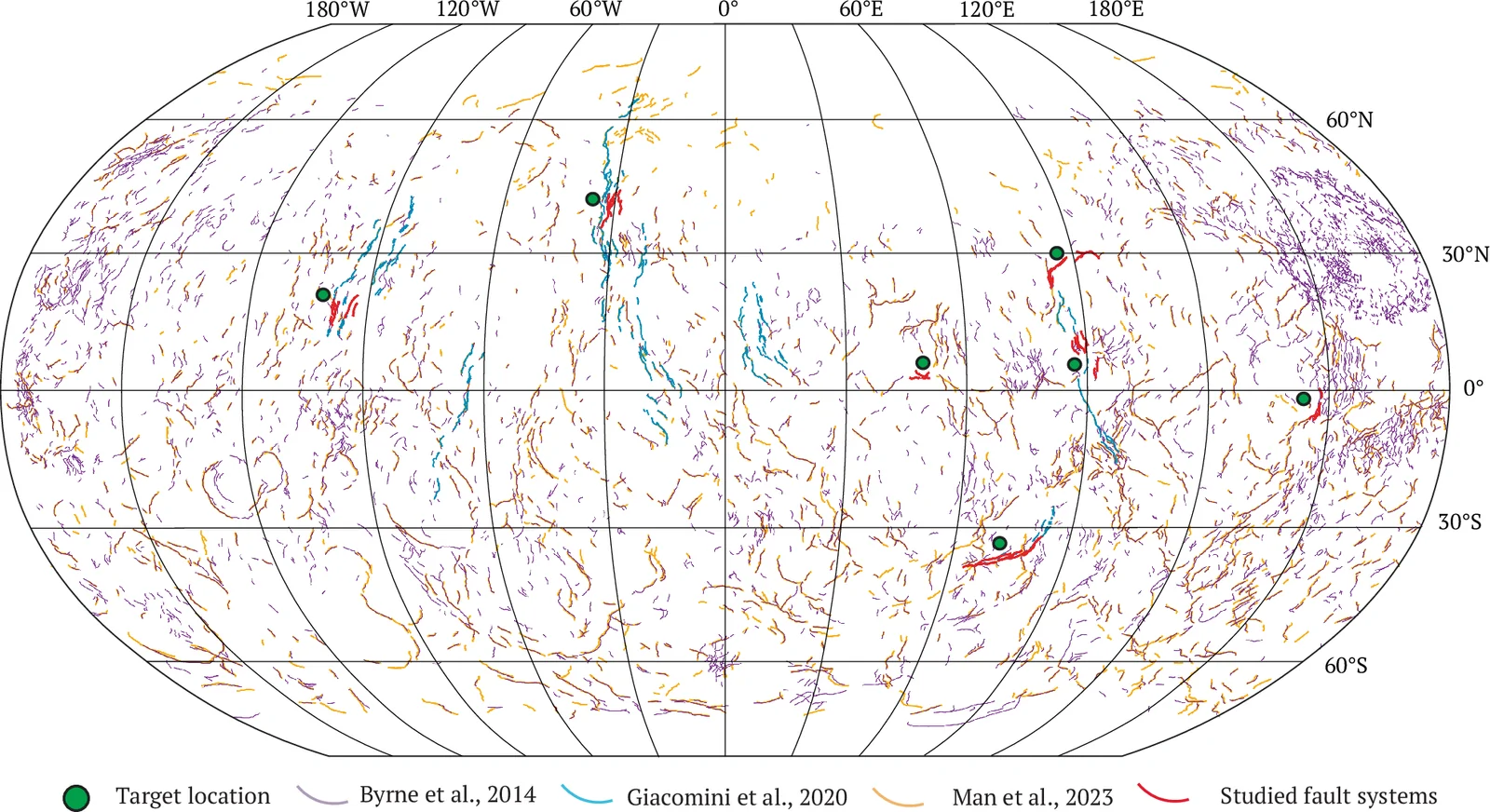
Shortening structures on Mercury^7,69,88^ shown in a Robinson projection centered at 0° latitude. The green circles represent the selected targets for this study, while the selected thrust systems are marked by solid red lines.

Lobate scarps are Mercury’s most characteristic and widely distributed contractional tectonic features. They vary in length from tens to hundreds of kilometers^1,2^ and are interpreted as the surface expressions of large thrust faults that accommodated the concurrent effect of early tidal despinning and planetary contraction driven primarily by secular cooling of Mercury’s core and mantle^3,4^. The estimated decrease of planetary radius widely ranges from 1-3 km^5,6^ to ~4-7 km^7^ (Fig. 1). A comprehensive interpretation of thrust-fault geometry, kinematics, and mechanics underlying lobate scarps remains elusive.

Earlier studies used simple elastic dislocation models, which are suitable for small deformation and assume “Andersonian” fault geometries^8,9^ to constrain fault parameters such as dip angle (i.e., ~30-35°), depth of faulting, and displacement of large-scale lobate scarps^10–14^. More recent investigations of the most prominent lobate scarps^15–19^ indicate that they can be interpreted as fold-and-thrust belts (FTBs^7^) and suggest the presence of regionally continuous low-angle thrust faults associated with hanging-wall anticlines. Indeed, low-angle thrusts associated with several lobate scarps have been directly derived from deformed craters^16,17^ and modeling^20^. However, the formation of low-angle faults is inconsistent with Mohr-Coulomb rheology, in which faults nucleate at 30-40° to the major principal stress σ_1_ (that is horizontal in contractional regimes in the context of Anderson’s theory^8,9^). Low-angle thrusts require the presence of mechanically weak layers to nucleate and need elevated pore-fluid overpressures^21^ and/or low-friction rocks to accumulate slip^22,23^. On Earth, weak layers are commonly constituted by bodies of salt, sulfates- or phyllosilicate-rich rocks with a low friction coefficient (μ < 0.25;^22,24–27^) and are often associated with elevated fluid pressure. However, fluid pressure favors slip on mechanically poorly oriented faults, but acts isotropically and does not favor the initial formation of low angle faults over optimally oriented ones (dipping 30-40°). Moreover, on Mercury, long-term and large-scale regional fluid overpressure and widespread hydrated weak rocks are not expected because of its anhydrous crust lacking any hydrologic cycle. Instead, Mercury’s surface is the most carbon-rich among the terrestrial planets, with an average carbon content of approximately 1.4 ± 0.9 wt.%^28–30^, thought to be in the form of graphite, a well-known solid lubricant (friction coefficient µ ~ 0.1)^31,32^. In particular, Low Reflectance Material (LRM), which covers about 15% of Mercury’s surface and exhibits reflectance values up to 30% below the global average^29,33^, is thought to be particularly enriched in graphite, with modeled global near-surface abundances on the order of 1 wt.%^28^, locally enriched up to ~4-5 wt%^29,34,35^ in the most concentrated low-reflectance deposits (e.g., dark spots, Rachmaninoff Basin^34,35^). This material is thought to be derived from a graphite-rich flotation crust formed during the early stages of crustal differentiation and subsequently excavated by large impacts^28,33,34,36^. Remnants of this layer, heterogeneously distributed and disrupted by early bombardment, may persist at depth beneath the present-day upper crust and, through impact gardening, be transported into shallower layers or locally exposed at the surface^29,33,34,36^. This redistribution occurs within a lithosphere already mechanically weakened by billions of years of impact bombardment, lava sheet cooling, thermal stresses, and the concurrent interplay of tidal despinning and global contraction as inferred from present tectonic fabrics^37,38^. In this setting, graphite can greatly reduce the strength in fault zones and in the crust^39–43^^,^, making it a strong candidate for facilitating slip in Mercurian faults.

**Fig. 2:**
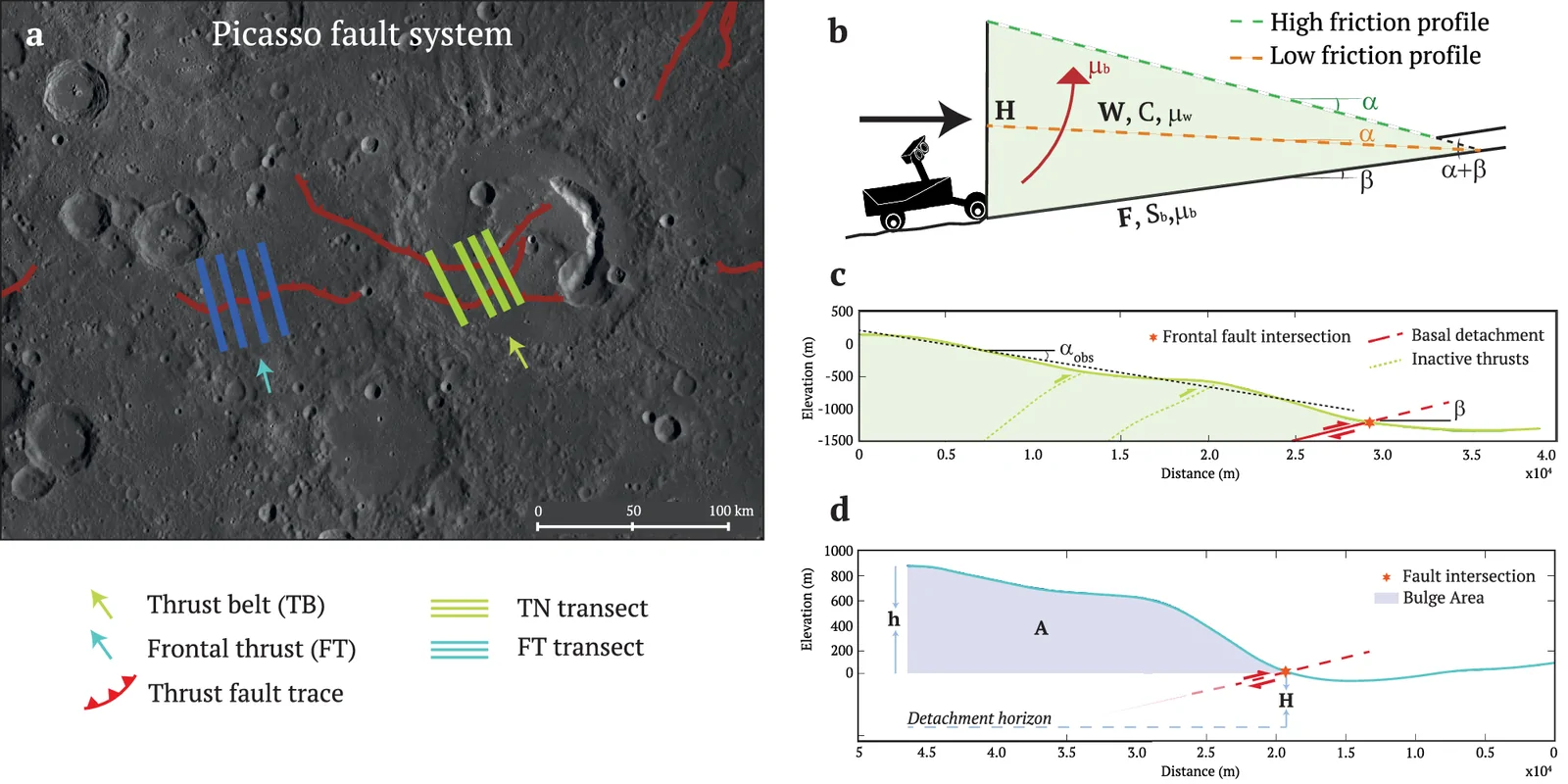
Modeling framework and topographic inputs of the Basal-Friction Model, illustrated for the Picasso Fault Systema. **a** The Picasso Fault System, shown in an equirectangular projection centered at 0° longitude. The green solid lines represent cross-section transects for the FTB, and the blue lines represent cross-section transects for the frontal thrust used in the Basal-Friction Model (BFM). **b** Cartoon representation of the Critical Taper Theory and the main parameters used for modeling. **H** represents the fault depth, α is the topographic profile angle, ß is the dip of the fault plane, **W** represents the strength parameters of the wedge including the cohesion (C) and friction coefficient (µ_w_) (see Methods, Eq. 1), and **F** represents the fault plane parameters, which include the basal cohesion (S_b_) and basal friction coefficient (µ_b_) (see Methods, Eq. 1). The straight black arrow shows the vergence of transport, while the red arrow indicates the theoretical increase of µ_b_ with a steeper profile. **c** Example of an averaged cross-section profile of the FTB, derived from multiple transects traced across the belt in the vergence direction (see panel **a**). These transects were used to extract the topographic profiles in QGIS and calculate the observed topographic angle (α_obs_). **d** Example (not to scale) of an averaged cross-section profile of the associated frontal thrust in the Picasso Fault System target, used to calculate the depth of the fault plane from the intersection point.

The mechanical framework that describes the mechanics of fold-and-thrust belts (FTBs) is the Critical Taper theory (CT^24,44^), which allows the modeling of large and steady crustal deformation. In the CT model, a wedge-shaped FTB results from the long-term equilibrium between compressive stresses, the strength of the wedge materials, and the friction along the basal detachment (Fig. 2b). If rocks obey a Coulomb-like behavior, friction along an inclined basal detachment sustains a topographic relief and a relatively constant slope of the wedge (α; see Fig. 2b, c), assumed to be critically stressed and on the verge of failure. Therefore, knowledge of the geometrical properties of the FTBs and the strength of the wedge materials allows estimating the strength of the basal detachment. In this work, we employ the CT theory, constrained by friction experiments, to derive the friction coefficient of the basal detachment (µ_b_) for thrust faults on Mercury using a Basal-Friction Model (BFM), which links the observed wedge taper to the strength of the wedge and of the basal detachment. Our approach highlights the crucial role of graphite-bearing layers in controlling the geometry and mechanics of Mercury’s major shortening landforms, linking surface expressions of crustal deformation to the composition of Mercury’s early crust.

## Results

### Fold-and-thrust belts: geometry and mechanical characterization

We selected seven lobate scarp systems, each several hundred kilometers long, interpreted as large fold-and-thrust belts across a broad portion of Mercury’s surface, each with sufficient topographic resolution for CT analysis of the basal friction (µ_b_) (Fig. 1; Supplementary Table 1). These structures share a characteristic arcuate morphology in map view and an asymmetric topographic profile in cross-section, characterized by a steep frontal slope and a gently sloping backlimb^1,2,18,45,46^. They represent the hanging walls of thrust faults, whose fold asymmetry records the direction of tectonic transport^7,47^. The observed topographic angle of these wedges (α_obs_), calculated from slopes in cross-section (Fig. 2a, c; Methods), ranges from very gentle (0.14°) to moderately inclined (4.24°), with an average of ~1.1° (Supplementary Table 1). Constraints on thrust geometry, specifically the basal dip (ß) of the studied thrust faults, come from crater-deformation studies, which treat faulted impact craters as passive strain markers^16,17^. In those cases, vertical rim offset and asymmetric hanging-wall uplift measured in digital terrain models (DTMs) are used to reconstruct the geometry of the shallow portion of the fault, yielding typical dip angles between ~5° and ~15° (Supplementary Fig. 1). Such shallow dips imply that Mercury’s crust is mechanically layered, including weak layers and low-angle faults that operate under effectively low-friction conditions^21,48^.

We independently estimated the detachment depths (H) using a fault-propagation-fold approach^49^ applied to the frontal thrust associated with each FTB (Fig. 2d; Methods). These depths vary significantly among the studied structures, from ~1.4 km at Unity Rupes to ~9.0 km at Endeavor Rupes, with an average of ~3.8 km (Supplementary Table 1).

**Fig. 3:**
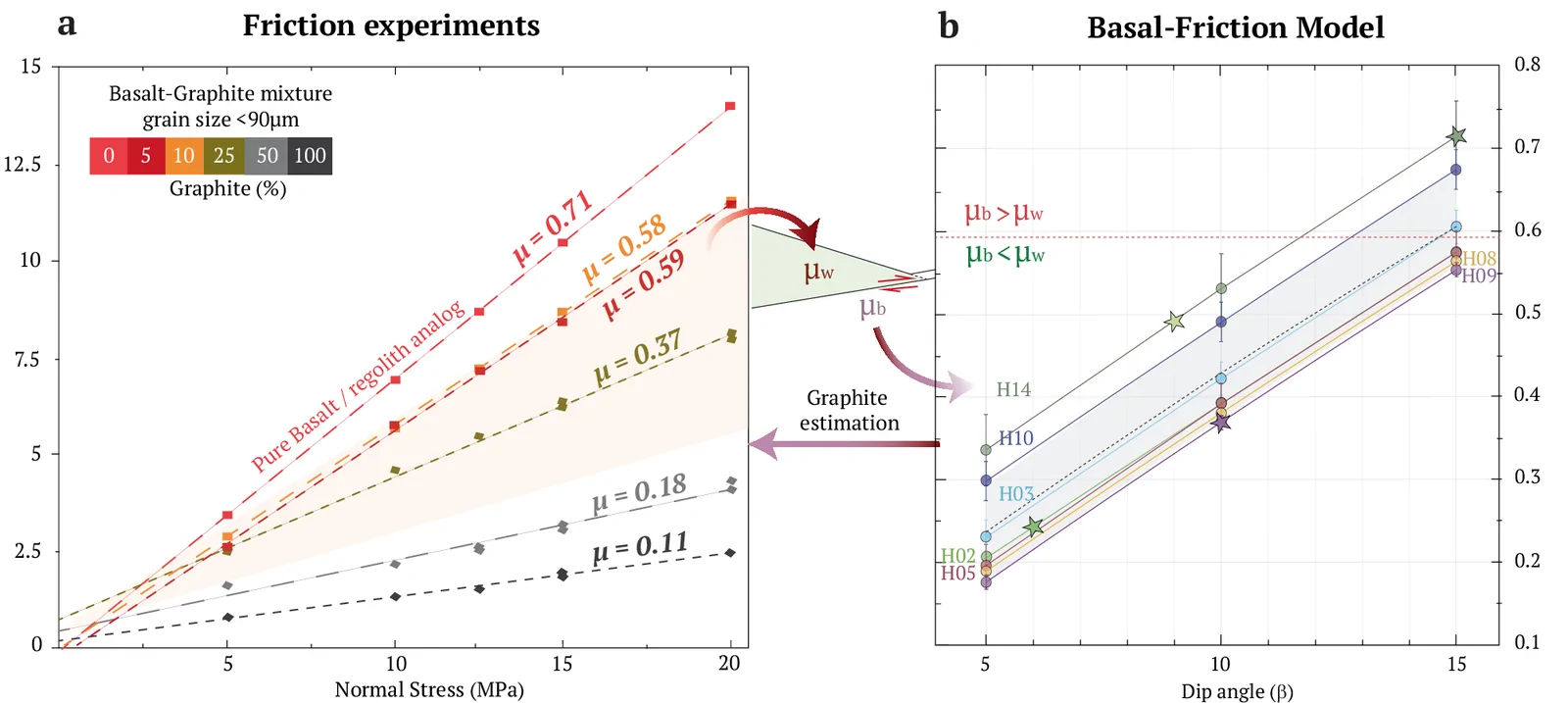
Rotary shear experiment results vs Basal-Friction Model (BFM). **a** Calculated Coulomb envelopes for a basalt-graphite gouge mixture deformed under dry conditions at increasing normal stress steps and at a shearing velocity of 10 µm/s. Each curve represents a different basalt-graphite proportion with their estimated µ value atop. The light orange area shows the range of calculated µ_b_ values. **b** Predicted basal coefficient of friction (µ_b_) values for the seven studied FTBs, identified by their quadrangle name. The average predicted µ_b_ values are represented in light gray. The red dashed line marks µ_w_ = 0.59, above which µ_b_ > µ_w_. The middle panel illustrates how the measured µ_w_ from friction experiments (i.e., 5% graphite slope) was used as an input for the BFM. The modeled µ_b_ values were then compared with friction experiments on various basalt/graphite mixtures that were used to constrain the amount of graphite on Mercury’s faults.

To characterize the friction of plausible graphite-bearing fault rocks, we sheared simulated fault gouges at slow velocity in a rotary shear apparatus (Methods). We directly tested the mechanical effect of graphite enrichment on mixtures of finely ( < 90 µm) crushed basalt and graphite under normal stresses of 5-20 MPa, comparable to ~2 km of overburden on Mercury (Fig. 3a; Methods). Notably, the grain size of the simulated gouges overlaps the average size fraction of lunar regolith (i.e., ~70 µm^50,51^) and, given the higher impact velocities on Mercury^52^, is also a reasonable analogue for Mercurian paleoregolith. For each gouge composition, we measured the shear stress during steady-state sliding in the experimental fault at different imposed normal stresses. The average rocks coefficient of friction, computed from the slope of linear regressions in Mohr space (shear vs. normal stress; Fig. 3a), ranges from µ = 0.71 (pure basalt), to µ = 0.58–0.59 (5-10% graphite, respectively), µ = 0.37-0.18 (25% and 50% graphite, respectively), and finally to µ = 0.11 (pure graphite), consistent with refs. ^32,39,53^. Considering a basaltic crust partially mixed with minor amounts of graphite (e.g., 5%; Fig.3a) consistent with the relative weak crust needed for the concurrent action of global contraction and tidal despinning^37,38^, and with the estimated surface content of LRM^29,34,35^ we used µ = 0.59 to constrain the internal friction of the wedge (µ_w_) and adopted low cohesive strengths of 0.5 MPa for both the wedge (C) and the detachment (S_b_). These values are consistent with previous work indicating that Mercury’s crust was already heavily fractured before the onset of global contraction^37,38,54^. With α_obs_, ß, H, µ_w_, and the cohesion values (C, S_b_) constrained as above, we used our BFM (Basal Friction Model) to estimate the basal friction coefficient (µ_b_) which under dry conditions dominates the effective strength of the basal detachment (F), with only a minor contribution from the detachment cohesion (S_b_), such that F = µ_b_ + S_b_/ρgH (Methods).

### Critical taper modeling of weak thrust faults

Using the CT parameters described above (Supplementary Table 1), we applied the BFM under dry Mercury conditions (i.e., negligible pore-fluid pressure) to estimate the maximum basal friction coefficient (µ_b_) consistent with the observed topography (α_obs_) across a range of basal dip angles ß = 5°-15°^16,17^(Methods). The resulting µ_b_ values represent the maximum friction allowed for slip on a given thrust in a critical tectonic wedge. Within this range, dip angles are independently constrained for three of our seven targets: Endeavor Rupes (H02, β = 6°^17^), Blossom System (β = 10°^16^), and Enterprise Rupes (β = 15° measured^16^, with a modeled minimum of 9°^20^).

The resulting maximum basal friction values range from µ_b_ ~ 0.17-0.76 across the seven thrust systems (Table 1; Fig. 3b; full outputs in Supplementary Table 1). At β = 5°, estimated µ_b_ reaches its minimum, ranging from ~0.18 to ~0.37 while Endeavor Rupes, independently constrained at β = 6°^17^, yields µ_b_ ~ 0.25. At β = 10°, values rise to 0.37-0.53. For the three directly constrained targets, µ_b_ remains well below µ_w_, with Blossom System at β = 10° yielding µ_b_ ~ 0.37, and Enterprise Rupes at its modeled minimum of β = 9° ^20^yielding µ_b_ ~ 0.5. At β = 15°, four systems (Blossom, Paramour, Unity, and Endeavor Rupes) remain below µ_w_, while Al-Hadmahani (µ_b_ ~ 0.61), Picasso System, and Enterprise Rupes at its measured upper dip (µ_b_ ~ 0.71) exceed µ_w_, indicating conditions inconsistent with critical taper. In this context, Enterprise Rupes and the Picasso fault system represent end-members in which µ_b_ > µ_w_. Enterprise Rupes shows a significant strike-slip component^18^, with frontal thrust dip angles ranging from a modeled average ~9°^20^ to a measured 15°^16^, and lateral ramp dips between ~30° and ~57°^16^. As already seen, the lower dip estimate is within physically admissible conditions for frictional sliding (i.e., µ_b_ = ~0.5). The high µ_b_ values at β = 15° most likely indicate that a simple frontal thrust model cannot account for the significant oblique component of this FTB. At β = 15°, the Picasso system yields an µ_b_ ~ 0.67, exceeding µ_w_, which under dry conditions would preclude slip. Thus, this dip angle becomes admissible only if pore-fluid pressure reduces the effective normal stress during fault activity, as might be supported by the proximity of this system to a pyroclastic volcanic vent^55^. Despite these potential exceptions, the majority of the analyzed FTBs yield µ_b_ below µ_w_ and below µ_b_ ~ 0.5 for dip angles between 5-10° (including the targets with independently constrained dip angles), suggesting that a weak solid material facilitated fault slip.

**Table 1:** Statistics of the calculated friction coefficient (µ_b_) *

Name	FTB	ß^1^	H^2^	α_obs_^3^	µ_b_^4^
		(°)	(m)	(°)	
H02 - Victoria	Victoria Rupes	5	2942	0.71 ± 0.05	0.21 ± 0.00
		10	5930	0.71 ± 0.05	0.39 ± 0.00
		15	9011	0.71 ± 0.05	0.58 ± 0.0
H03 - Shakespeare	Al-Hadmahani System	5	1829	1.25 ± 0.4	0.23 ± 0.02
		10	3687	1.25 ± 0.4	0.42 ± 0.02
		15	5603	1.25 ± 0.4	0.61 ± 0.02
H05 - Hokusai	Unity Rupes	5	1391	0.78 ± 0.5	0.20 ± 0.03
		10	2804	0.78 ± 0.5	0.39 ± 0.03
		15	4261	0.78 ± 0.5	0.58 ± 0.03
H08 - Tolstoj	Paramour Rupes	5	1803	0.54 ± 0.1	0.19 ± 0.00
		10	3633	0.54 ± 0.1	0.38 ± 0.00
		15	5521	0.54 ± 0.1	0.56 ± 0.00
H09 - Eminescu	Blossom System	5	1588	0.35 ± 0.2	0.18 ± 0.01
		10	3201	0.35 ± 0.2	0.37 ± 0.01
		15	4864	0.35 ± 0.2	0.55 ± 0.01
		5	1627	2.60 ± 0.4	0.30 ± 0.02
H10 -Derain	Picasso fault System	10	3280	2.46 ± 0.4	0.49 ± 0.02
		15	4984	2.60 ± 0.4	0.67 ± 0.02
		5	1350	3.37 ± 0.8	0.34 ± 0.04
H14 - Debussy	Enterprise Rupes	10	2720	2.50 ± 0.5	0.53 ± 0.04
		15	4134	3.38 ± 0.8	0.71 ± 0.04

^1^ Fault dip angle

^2^ Depth of the décollement

^3^ Average observed topographic angle

^4^ Average calculated friction coefficients of the fault plane

^*^Cohesion of the fault plane (S_b_) and Cohesion of the wedge (C) = 0.5 MPa

^*^Friction coefficient of the wedge (µ_w_) = 0.59

We then compare these µ_b_ values inferred for the basal detachments of our case studies with the results of the rotary-shear friction experiments (Fig. 3a). Experimental results show that fault gouges with graphite enrichments on the order of ~5-10 vol% and up to ~40 vol% (Supplementary Fig. 4) successfully reproduce the range of modeled weak thrusts, therefore graphite-rich fault gouge is a suitable candidate for this solid lubrication mechanism.

## Discussion

### Graphite lubrication and the origin of weak detachments on Mercury

Mercury’s crust is layered and anisotropic, and includes low-angle thrust faults whose fault rocks must be weak compared to typical silicate rocks to be able to slip (i.e., commonly µ ~ 0.6-0.8 for crystalline silicates in upper crustal conditions^56^). On Earth, comparably weak detachments in thrust belts are usually associated with viscous evaporitic layers, phyllosilicate-rich gouges with very low friction (µ < 0.25)^22,57–59^, or water-saturated and occasionally overpressured shales^25,60,61^. However, several of these terrestrial weakening mechanisms are unlikely to operate at a planetary scale on Mercury. Evaporitic minerals require persistent surface (or near-surface) brines and fluid circulation to accumulate, and phyllosilicates (e.g., clay minerals and micas) are either associated with siliciclastic sedimentation in aqueous environments or with diagenesis and hydrothermal alteration^23^. Such conditions are not expected on Mercury, where liquid water can only be stable as ice within the permanent shadowed regions^62^. In addition, global spectral data show no diagnostic ~1.4 µm absorption bands typical of hydrated minerals (including phyllosilicates and evaporites)^63^.

Long-term, regionally elevated pore-fluid pressures are difficult to sustain in a highly fractured, impact-disrupted crust with no long-lived hydrologic cycle. Even if explosive volcanism and volatile-driven degassing have been documented on Mercury by the presence of pyroclastic vents^64–66^and faculae^67,68^, this activity is both spatially limited and transient, and is therefore unlikely to have maintained elevated pore pressures across the hundreds-to-thousands of kilometers scales and ~100 Myr timescales characteristic of large FTBs^4,69^.

By contrast, the existence of a graphite-enriched layer is particularly likely, since it may have constituted a primary graphite flotation crust during the early stages of the planet’s formation^28,36^. Major basin-forming impacts (e.g., Rembrandt, Rachmaninoff, and Tolstoj) are thought to have excavated and locally exposed fragments of this primordial layer^29,33,34,36^. Notably, our analysis of Mercury’s fold-and-thrust belts suggests that remnants of this graphite-rich layer may locally be preserved beneath portions of the present upper crust. In addition, Mercury’s upper crust was likely already heavily fractured before large-scale contraction began, due to early impact gardening, lava sheets cooling, and thermal stresses^37^, in a way analogous to the intensely damaged shallow crust observed on the Moon by the GRAIL mission^70^. This implies that deformation in Mercury’s thrust fault systems localized within pre-fractured, megaregolith-like material mixed with a minor amount of graphite rather than intact basalt, suggesting that the crust strength was reduced before any major tectonic compression. Within this framework, the frictional behavior of the simulated crust composition of our experiments (Fig. 3a; µ_w_ = 0.59) represents that of a damaged basaltic crust interleaved with paleoregolith layers, transected by inherited anisotropies underlined by fine gouges and intermixed with a slight admissible content of graphite. Comparing these experimental results with the µ_b_ values inferred from BFM (Fig. 3c), we suggest that the required weakness corresponds to mixtures containing from 5-10 vol.% up to 40 vol.% graphite (Fig. 3a). This concentration of graphite is likely confined to the principal slip zones rather than the bulk rock. Typically, slip zones on Earth are ~mm or even ~sub-mm thick^48,71^. Thus, graphite was likely progressively dragged into slip zones from deep graphite-rich layers or pockets through shear-parallel smearing^53^, facilitating the localization of deformation along fault slip surfaces (Supplementary Fig. 5). In this context, our modeling and experiments indicate that graphite-rich material along low-angle anisotropies is a plausible weakening agent for the low friction coefficients inferred along Mercury’s thrust detachments, and together they provide a mechanically consistent explanation for the observed contractional landforms.

### From primordial differentiation to graphite-controlled shrinking

**Fig. 4:**
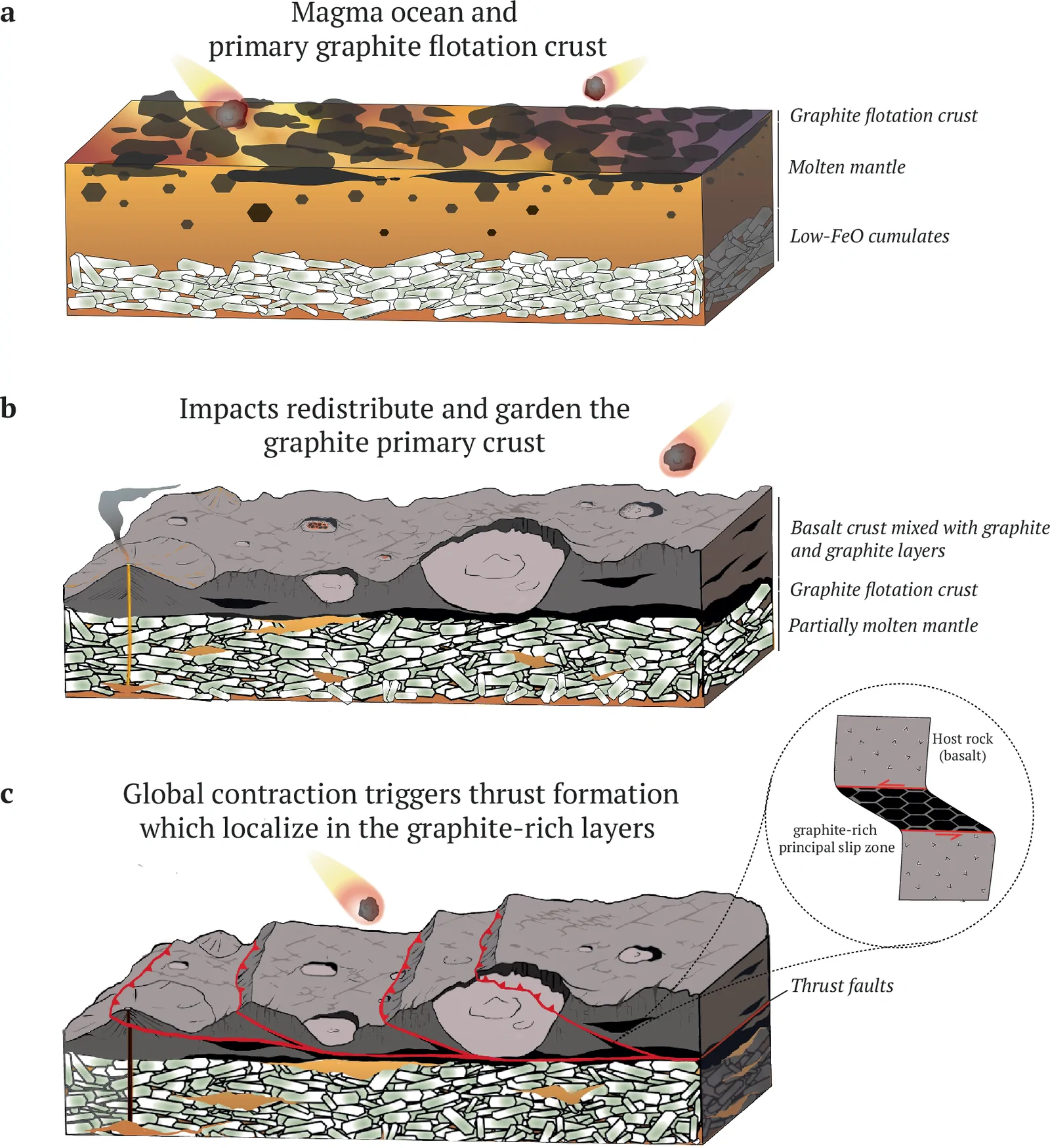
Cartoon (not to scale) of the proposed scenario for the evolution of Mercury’s crust. **a** formation of a heterogeneous flotation graphite crust during early differentiation; **b** formation of a secondary crust heavily fractured during a pre-contraction stage; **c** contraction stage controlled by inherited anisotropies and graphite rich pockets and layers that through interconnection, fault weakening and shear parallel smearing led to the formation of large-scale FTBs.

Considering that other known weakening mechanisms (e.g., evaporites, phyllosilicate-rich gouges, long-term fluid overpressure) are unlikely under Mercury’s crustal conditions, our results indicate that the low basal friction required to reproduce the observed surface deformation in several major fold-and-thrust systems is most consistently explained by graphite-bearing horizons. Current models of early Mercury’s history suggest a magma ocean stage^72,73^ with prevailing reducing conditions during early differentiation. Given the limited density range of Mercury’s melts^36^, a primary graphite flotation crust (Fig. 4a) should have formed from carbon originally dissolved in the iron-poor silicate melt of Mercury’s magma ocean^28–30,36,74,75^. As the buoyant crust progressively cooled, ongoing impacts would have locally disrupted and thinned it. Over time, this led to the formation of a heterogeneous primordial crust, reflecting both the initial conditions of crystallization and the subsequent impact-driven modifications that shaped Mercury’s earliest crustal architecture. In Fig. 4b, this stage is followed by mantle partial melting and widespread volcanic resurfacing, forming a secondary crust. Continuous impacts to the planet fractured and reshuffled the primary and secondary crust, resulting in a dense fracture network (mega-regolith-like) and graphite-rich pockets and layers distributed at different crustal depths. Low-reflectance material (LRM) observed at the surface today, e.g. ref. ^34^, is further evidence of such process. LRM thickness locally exceeds 7 km^76,77^, and its graphite content is estimated to range between 1 and 5 wt%^28,29,78^. Thus, the graphite flotation crust must have been significantly mixed into the early-forming secondary crust, and graphite abundances are likely higher at depth. Interestingly, LRM deposits are found in several locations across the planet, but not all large craters and basins have excavated LRMs^33,76^, indicating a heterogeneous crustal distribution.

As shown in Fig. 4c, the onset of global contraction led to fault nucleation and slip favored by interconnection of inherited anisotropies and the presence of graphitic pockets and laterally confined horizons. Smearing along the slip zone allowed graphite to be dragged upward from graphite-rich layers and pockets through the crust, enabling the growth of large-scale thrust belts accommodating Mercury’s contraction. The graphite abundance that experimentally explains the weak fault behavior goes from 5-10 vol.% up to 40 vol.% However, given the relatively low graphite abundances suggested for LRM deposits^29,34,35^, even allowing for higher subsurface concentrations, the total volume of this dispersed weak phase within the crust would likely be insufficient to produce significant weakening without considering two factors of paramount importance: (1) the amount of graphite needed to induce weakness can be confined within very narrow films (i.e., ~mm or even ~sub-mm) along the principal slip zone^48,71^, and (2) the effective coating of a weak phase (graphite) on a slip plane results from mechanical smearing^53^ (Supplementary Fig. 5). This allows a relatively small volume fraction of weak material, at the planetary scale, to have an overly large effect on fault strength.

Our work suggests that graphite smearing along crustal fault planes provides a robust explanation for the geometry and kinematics of the large-scale fold-and-thrust belts observed today on Mercury’s surface, which would otherwise be mechanically unconceivable. Consequently, Mercury’s early magma ocean differentiation led to a unique, graphite-dominated global tectonic regime in the Solar System, demonstrating how critically early planetary evolution controls later crustal mechanics.

### Global implications of weak and low-angle faults

**Fig. 5:**
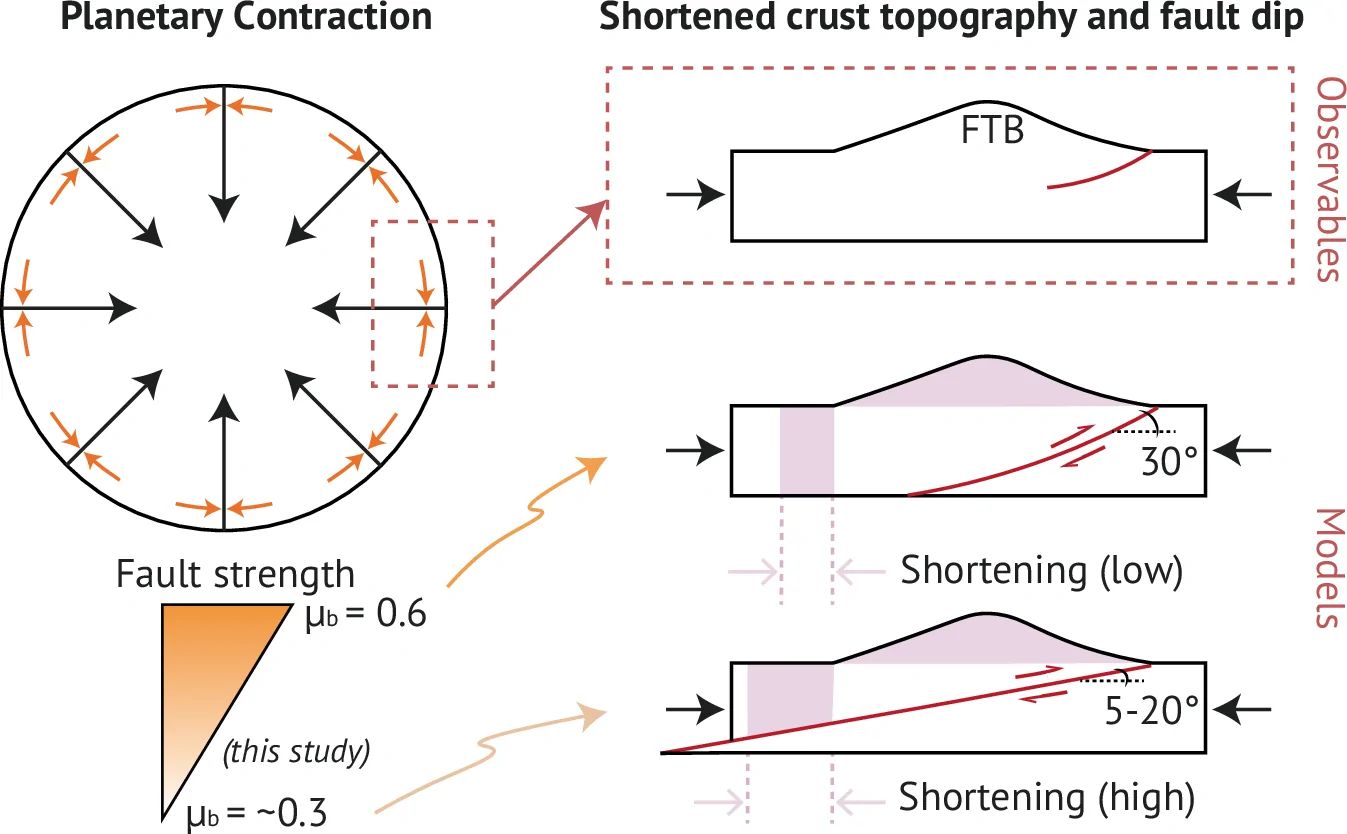
Schematic cartoon (not to scale) illustrating the implications of FTB geometry and fault strength (µ_b_) for Planetary contraction of Mercury. Lower fault strength and shallower thrust faults require more shortening compared to more steeply dipping thrusts. The end-member µ_b_ ~ 0.3 represents the estimated basal friction coefficient for Endeavor Rupes (H02), for which the dip angle is well constrained^17^.

In this work, we have demonstrated the importance of low-angle thrust faults (5°−15°) in accommodating Mercury’s global contraction (Fig. 5). The quantification of Mercury’s global contraction is strongly influenced by assumptions regarding the dip angles (ß) of thrust faults and their regional mapping. Previous estimates of Mercury’s contraction^5,7,79–81^ are based on traditional Andersonian fault theory, which predicts that most thrust faults form at moderate to high dip angles (typically 25°-40°)^8^, depending on the internal coefficient of friction of rocks, generally assumed in the Byerlee range (i.e., µ = 0.6-0.85)^71^. The different amounts of contraction estimated by various authors mainly depend on the structures assumed to be responsible for global deformation: only major lobate scarp systems (i.e., fold-and-thrust belts) for Watters et al. (2021), who envisaged a radial contraction between ~1.3-2 km, or the majority of lobate scarps and wrinkle ridges distributed across the surface (excluding features within major basins such as Caloris and Rembrandt) for Byrne et al. (2014), who suggested up to 7 km of contraction. Regardless of the final estimate, the assumption of Andersonian fault behavior contradicts the low thrust fault dip angles derived by crater deformation analysis^16,17^, recent modeling efforts on large FTBs^20^, and our CT fits of the observed surface morphology of faults and thrust belts. If a significant fraction of Mercury’s lobate scarps are the surface expressions of low-angle thrust faults, the total horizontal shortening -and thus the inferred global contraction- should be substantially higher than previous conservative estimates. In this context, we encourage a future revision of the global catalogue of contractional features to include low-angle thrust faults, as the range of contraction estimates, and thus the interpretations of the planet’s thermal evolution and internal composition, remain highly sensitive to structural assumptions.

## Methods

### Data and target selection

For the creation of the cross-section topographic profiles and target selection, we used the 665 m/pixel digital elevation model (DEM) (available at https://astrogeology.usgs.gov/search/map/mercury_messenger_global_dem_665m) and the ~166 m/pixel Global Map projected Basemap (BDR) (available at https://astrogeology.usgs.gov/search/map/Mercury/Messenger/Global/Mercury_MESSENGER_MDIS_Basemap_BDR_Mosaic_Global_166m) acquired by the Mercury Surface, Space Environment, Geochemistry and Ranging (MESSENGER) Mercury Dual Imaging System (MDIS). Targets were selected using published surface traces of contractional features. Each one of the seven targets selected across Mercury’s surface comprises 2 surface features: a thrust belt zone (i.e., a zone characterized by two or more thrust faults with the same vergence and a wedge-shaped cross-section profile) for mechanical calculations, along with a frontal thrust of the same system used for geometry assessment (i.e., detachment depth calculation). Each target was divided into a series of profiles (cross sections) to carry out the Basal Friction Model (BFM) (Supplementary Table 1).

### Geometric model

The geometric model, implemented in MATLAB, is based on a fault-propagation-folding geometry^49,82^, and is used to calculate the amount of shortening (S_h_; Eq. 1) and the detachment depth (H; Eq. 2). For each target, multiple cross-sections were traced, and the mean of these transects was used to calculate the geometry, following the equations below:

1 $${{{\rm{S}}}}_{{{\rm{h}}}}=\cot {{\rm{\beta }}}*{{\rm{h}}}$$

2 $$H=\,\frac{A}{{S}_{h}}$$

Where β represents the dip angle of the décollement, h represents the maximum thickness of the bulge, and A represents the bulge area calculated through trapezoidal integration (Fig. 2d of the main text). The geometry for each target was calculated using the average cross-section.

### Mechanical model

The Basal-Friction Model (BFM), implemented in MATLAB, relies on the Critical Taper theory and uses the simplified relations from Suppe (2007) based on the weak-base theory of Dahlen, 1990^44^ (Eqs. 3-5). BFM directly computes the basal friction coefficient (µ_b_) for each FTB cross-section, given the detachment depth H (from geometric analysis; Methods, Geometric Model), the observed topographic angle (α_obs_), and the material parameters. The latter are the coefficient of friction of the wedge (μ_w_) derived from our experiments considering a crust partially mixed with about 5% vol. of graphite, and the wedge and basal cohesions C and S_b_ (0.5 MPa each) derived supported by literature^37,38,54^. We also explore fault dips (β**)** from independent measurements by Galluzzi et al. (2015, 2019) and Loveless et al. (2025). In the equations below, F and W represent the nondimensional décollement and wedge strengths, respectively; φ = arctan(µ_w_) is the internal friction angle; g is the acceleration due to gravity (3.7 m/s^2^)^83,84^; ρ is the average density of the wedge material (3340 kg/m³; e.g^30,85^.); μ_b_ is the basal coefficient of friction, ρ_f_ is the density of the overlying fluid ( ≈ 0 here); λ is the pore-fluid pressure ratio; and C and S_b_ are the cohesion terms (0.5 MPa). The model assumes airless, dry conditions with negligible pore-fluid pressure (P_f_ ≈ 0 so the pore-fluid pressure ratio λ = P_f_ /ρgH ≈ 0 and Eqs. 3-4 reduce to 6-7) and no overlying fluid, (ρ_f_ ≈ 0, hence ρ_f/_ρ ≈ 0 and Eq. 5 reduces to Eq. 8). μ_b_ is computed analytically for β ∈ {5°, 10°, 15°}, reporting the resulting μ_b_ values (Supplementary Table 1).

3 $$F=\,{{{\rm{\mu }}}}_{b}\left(1-\lambda \right)+\,\frac{{S}_{b}}{\rho {gH}}$$

4 $$W=2\left(1-\lambda \right)\left[\frac{\sin {{\rm{\varphi }}}}{1-\sin {{\rm{\varphi }}}}\right]+\frac{C}{\rho {gH}}$$

5 $$\alpha=\frac{F}{\left[1-\left(\frac{{\rho }_{f}}{\rho }\right)\right]+W}+\frac{W}{\left[1-\left(\frac{{\rho }_{f}}{\rho }\right)\right]+W}\,{{\rm{\beta }}}$$

In dry, airless conditions on Mercury, ρ_f_ and λ are negligible^86^, yielding the simplified forms in Eqs. 6-8.

6 $$F=\,{{{\rm{\mu }}}}_{b}+\frac{{S}_{b}}{\rho {gH}}$$

7 $$W=2\left[\frac{\sin {{\rm{\varphi }}}}{1-\sin {{\rm{\varphi }}}}\right]+\frac{C}{\rho {gH}}$$

8 $$\alpha=\frac{F}{1+W}+\frac{W}{1+W}\,{{\rm{\beta }}}$$

The details of the script workflow and pseudocode are shown in Supplementary Figs. 2, 3.

### Friction experiments

We used the *Ro*tary-*S*hear *A*pparatus (RoSA)^87^ at the Department of Geosciences of Padua University (Padua, Italy) for the friction experiments. We sheared samples composed of basalt and graphite mixtures considered as representative endmembers of Mercury’s basaltic crust/paleoregolith and of the most likely weak phase at depth, respectively. Samples are finely ground powders obtained by grinding in a disk mill and sieving down to grain size <90 µm. Powders of each rock, basalt and graphite, were then mixed in six different proportions: 100% basalt (pure basaltic crust endmember), 95% basalt 5% graphite (assumed weak crust due to limited graphite content), 90% basalt 10% graphite, 75% basalt 25% graphite, 50% basalt 50% graphite, and 100% graphite (primordial graphite layer). 4 grams of each mixture were then sandwiched between two grooved sample holders to form 3mm-thick layers that simulate the fault slip surface and mounted on the RoSA apparatus. Each test was carried out at a shearing velocity of 10 µm/s and imposing normal stresses (σ_n_) from 5 to 20 MPa, under dry conditions and 25 °C. Samples were sheared for 5 mm at each normal stress, until a constant shear stress during dynamic frictional sliding was attained in each step. Data were collected at a frequency of 10 Hz. Details of the RoSA apparatus, sensor measurements and experimental procedures follow Rempe et al.^80^. We measured the shear stress (τ) during stable sliding of the gouges at each normal stress and determined the average friction of each mixture by the slope of the linear Coulomb envelope.

## Supplementary information

Supplementary Information

Transparent Peer Review file

## Data Availability

For the creation of topographic cross sections and target selection, we used the global digital elevation model of Mercury at 665 m pixel⁻¹ resolution and the ~166 m pixel⁻¹ global MDIS basemap (BDR), both acquired by the MESSENGER Mercury Dual Imaging System (MDIS) and publicly available through the USGS Astrogeology Science Center. Published surface traces of contractional tectonic features were used to define the target regions (Byrne et al., 2014; Giacomini et al., 2020; Man et al., 2023). All data generated in this study and the related raw data, including cross-section topographic profiles, geometrical measurements, a QGIS project to reproduce Fig. 1, and outputs of the Basal Friction Model (BFM), are provided as Source Data with this paper and are available via Figshare (10.6084/m9.figshare.30885902).

## Code availability

All custom code used for the mechanical modeling (BFM) is available in the Code Ocean capsule created for this study at 10.24433/CO.6220849.v1. The capsule contains the full analysis workflow, documentation, and all dependencies required to reproduce the results.
