# Supplementary material for: Graphite lubricates Mercury’s global contraction
Source: Nat Commun. 2026 Aug 26;17:9063. doi: 10.1038/s41467-026-75459-x (PMC13518970; doi:10.1038/s41467-026-75459-x)
Supplement: Supplementary file 1 [file 41467_2026_75459_MOESM1_ESM.pdf]

Supplementary Information

Supplementary Table 1. **Parameters and Outputs of BFM for each target**

Target	Quadrangle	FTB[1]	Profile	ß[2]	H[3]	C[4]	S[5]	μ_w[6]	α_{obs}[7]	μ_b[8]
				(°)	*(m)*	*(MPa)*	*(MPa)*		*(°)*	
1	H02 - Victoria	Endeavour Rupes	P1_ER	5	2942	0.5	0.5	0.59	0.75	0.21
			P2_ER	5	2942	0.5	0.5	0.59	0.67	0.20
			P3_ER	5	2942	0.5	0.5	0.59	0.64	0.20
			P4_ER	5	2942	0.5	0.5	0.59	0.76	0.21
			P1_ER	10	5930	0.5	0.5	0.59	0.75	0.40
			P2_ER	10	5930	0.5	0.5	0.59	0.67	0.39
			P3_ER	10	5930	0.5	0.5	0.59	0.64	0.39
			P4_ER	10	5930	0.5	0.5	0.59	0.76	0.40
			P1_ER	15	9011	0.5	0.5	0.59	0.75	0.58
			P2_ER	15	9011	0.5	0.5	0.59	0.67	0.57
			P3_ER	15	9011	0.5	0.5	0.59	0.64	0.57
			P4_ER	15	9011	0.5	0.5	0.59	0.76	0.58
2	H03 - Shakespeare	Al-Hadmahani system	P1_AH	5	1829	0.5	0.5	0.59	1.51	0.24
			P2_AH	5	1829	0.5	0.5	0.59	1.57	0.24
			P3_AH	5	1829	0.5	0.5	0.59	1.53	0.24
			P4_AH	5	1829	0.5	0.5	0.59	1.46	0.24
			P5_AH	5	1829	0.5	0.5	0.59	1.16	0.22
			P6_AH	5	1829	0.5	0.5	0.59	0.61	0.19
			P1_AH	10	3687	0.5	0.5	0.59	1.51	0.43
			P2_AH	10	3687	0.5	0.5	0.59	1.57	0.44
			P3_AH	10	3687	0.5	0.5	0.59	1.53	0.43
			P4_AH	10	3687	0.5	0.5	0.59	1.46	0.43
			P5_AH	10	3687	0.5	0.5	0.59	1.16	0.41
			P6_AH	10	3687	0.5	0.5	0.59	0.61	0.38
			P1_AH	15	5603	0.5	0.5	0.59	1.51	0.62

			P2_AH	15	5603	0.5	0.5	0.59	1.57	0.62
			P3_AH	15	5603	0.5	0.5	0.59	1.53	0.62
			P4_AH	15	5603	0.5	0.5	0.59	1.46	0.61
			P5_AH	15	5603	0.5	0.5	0.59	1.16	0.60
			P6_AH	15	5603	0.5	0.5	0.59	0.61	0.57
3	H05 - Hokusai	Unity Rupes	P1_UR	5	1391	0.5	0.5	0.59	1.11	0.21
			P2_UR	5	1391	0.5	0.5	0.59	0.57	0.18
			P3_UR	5	1391	0.5	0.5	0.59	0.38	0.17
			P4_UR	5	1391	0.5	0.5	0.59	0.38	0.17
			P5_UR	5	1391	0.5	0.5	0.59	1.45	0.23
			P1_UR	10	2804	0.5	0.5	0.59	1.11	0.41
			P2_UR	10	2804	0.5	0.5	0.59	0.57	0.38
			P3_UR	10	2804	0.5	0.5	0.59	0.38	0.37
			P4_UR	10	2804	0.5	0.5	0.59	0.38	0.37
			P5_UR	10	2804	0.5	0.5	0.59	1.45	0.43
			P1_UR	15	4261	0.5	0.5	0.59	1.11	0.59
			P2_UR	15	4261	0.5	0.5	0.59	0.57	0.56
			P3_UR	15	4261	0.5	0.5	0.59	0.38	0.55
			P4_UR	15	4261	0.5	0.5	0.59	0.38	0.55
			P5_UR	15	4261	0.5	0.5	0.59	1.45	0.61
4	H08 - Tolstoj	Paramour Rupes	P1_PR	5	1803	0.5	0.5	0.59	0.59	0.19
			P2_PR	5	1803	0.5	0.5	0.59	0.48	0.19
			P3_PR	5	1803	0.5	0.5	0.59	0.48	0.19
			P4_PR	5	1803	0.5	0.5	0.59	0.47	0.19
			P5_PR	5	1803	0.5	0.5	0.59	0.65	0.20
			P1_PR	10	3633	0.5	0.5	0.59	0.59	0.38
			P2_PR	10	3633	0.5	0.5	0.59	0.48	0.38
			P3_PR	10	3633	0.5	0.5	0.59	0.48	0.38
			P4_PR	10	3633	0.5	0.5	0.59	0.47	0.38

			P5_PR	10	3633	0.5	0.5	0.59	0.65	0.39
			P1_PR	15	5521	0.5	0.5	0.59	0.59	0.57
			P2_PR	15	5521	0.5	0.5	0.59	0.48	0.56
			P3_PR	15	5521	0.5	0.5	0.59	0.48	0.56
			P4_PR	15	5521	0.5	0.5	0.59	0.47	0.56
			P5_PR	15	5521	0.5	0.5	0.59	0.65	0.57
5	H09 - Eminescu	Blossom System	P1_BR	5	1588	0.5	0.5	0.59	0.32	0.17
			P2_BR	5	1588	0.5	0.5	0.59	0.57	0.19
			P3_BR	5	1588	0.5	0.5	0.59	0.42	0.18
			P4_BR	5	1588	0.5	0.5	0.59	0.30	0.17
			P5_BR	5	1588	0.5	0.5	0.59	0.14	0.16
			P1_BR	10	3201	0.5	0.5	0.59	0.32	0.37
			P2_BR	10	3201	0.5	0.5	0.59	0.57	0.38
			P3_BR	10	3201	0.5	0.5	0.59	0.42	0.37
			P4_BR	10	3201	0.5	0.5	0.59	0.30	0.37
			P5_BR	10	3201	0.5	0.5	0.59	0.14	0.36
			P1_BR	15	4864	0.5	0.5	0.59	0.32	0.55
			P2_BR	15	4864	0.5	0.5	0.59	0.57	0.57
			P3_BR	15	4864	0.5	0.5	0.59	0.42	0.56
			P4_BR	15	4864	0.5	0.5	0.59	0.30	0.55
			P5_BR	15	4864	0.5	0.5	0.59	0.14	0.54
6	H10 - Derain	Picasso fault system	P1_PC	5	1627	0.5	0.5	0.59	2.52	0.29
			P2_PC	5	1627	0.5	0.5	0.59	2.05	0.27
			P3_PC	5	1627	0.5	0.5	0.59	2.36	0.28
			P4_PC	5	1627	0.5	0.5	0.59	3.17	0.33
			P5_PC	5	1627	0.5	0.5	0.59	2.91	0.31
			P1_PC	10	3280	0.5	0.5	0.59	2.52	0.49
			P2_PC	10	3280	0.5	0.5	0.59	2.05	0.46
			P3_PC	10	3280	0.5	0.5	0.59	2.36	0.48

			P4_PC	10	3280	0.5	0.5	0.59	3.17	0.52
			P5_PC	10	3280	0.5	0.5	0.59	2.91	0.51
			P1_PC	15	4984	0.5	0.5	0.59	2.52	0.67
			P2_PC	15	4984	0.5	0.5	0.59	2.05	0.65
			P3_PC	15	4984	0.5	0.5	0.59	2.36	0.66
			P4_PC	15	4984	0.5	0.5	0.59	3.17	0.70
			P5_PC	15	4984	0.5	0.5	0.59	2.91	0.69
7	H14 - Debussy	Enterprise Rupes	P1_EntR	5	1350	0.5	0.5	0.59	3.96	0.37
			P2_EntR	5	1350	0.5	0.5	0.59	3.88	0.36
			P3_EntR	5	1350	0.5	0.5	0.59	4.24	0.38
			P4_EntR	5	1350	0.5	0.5	0.59	3.20	0.33
			P5_EntR	5	1350	0.5	0.5	0.59	2.88	0.31
			P6_EntR	5	1350	0.5	0.5	0.59	2.12	0.27
			P1_EntR	10	2720	0.5	0.5	0.59	3.96	0.56
			P2_EntR	10	2720	0.5	0.5	0.59	3.88	0.56
			P3_EntR	10	2720	0.5	0.5	0.59	4.24	0.58
			P4_EntR	10	2720	0.5	0.5	0.59	3.20	0.52
			P5_EntR	10	2720	0.5	0.5	0.59	2.88	0.50
			P6_EntR	10	2720	0.5	0.5	0.59	2.12	0.46
			P1_EntR	15	4134	0.5	0.5	0.59	3.96	0.75
			P2_EntR	15	4134	0.5	0.5	0.59	3.88	0.74
			P3_EntR	15	4134	0.5	0.5	0.59	4.24	0.76
			P4_EntR	15	4134	0.5	0.5	0.59	3.20	0.71
			P5_EntR	15	4134	0.5	0.5	0.59	2.88	0.69
			P6_EntR	15	4134	0.5	0.5	0.59	2.12	0.65

[1] Studied fold-and-thrust belt system

[2] Fault dip angle

[3] Fault plane depth

[4] Cohesion of the wedge

[5] Cohesion of the fault plane

[6] Friction coefficient of the wedge

[7] Observed topographic angle

[8] Calculated friction coefficient of the fault plane

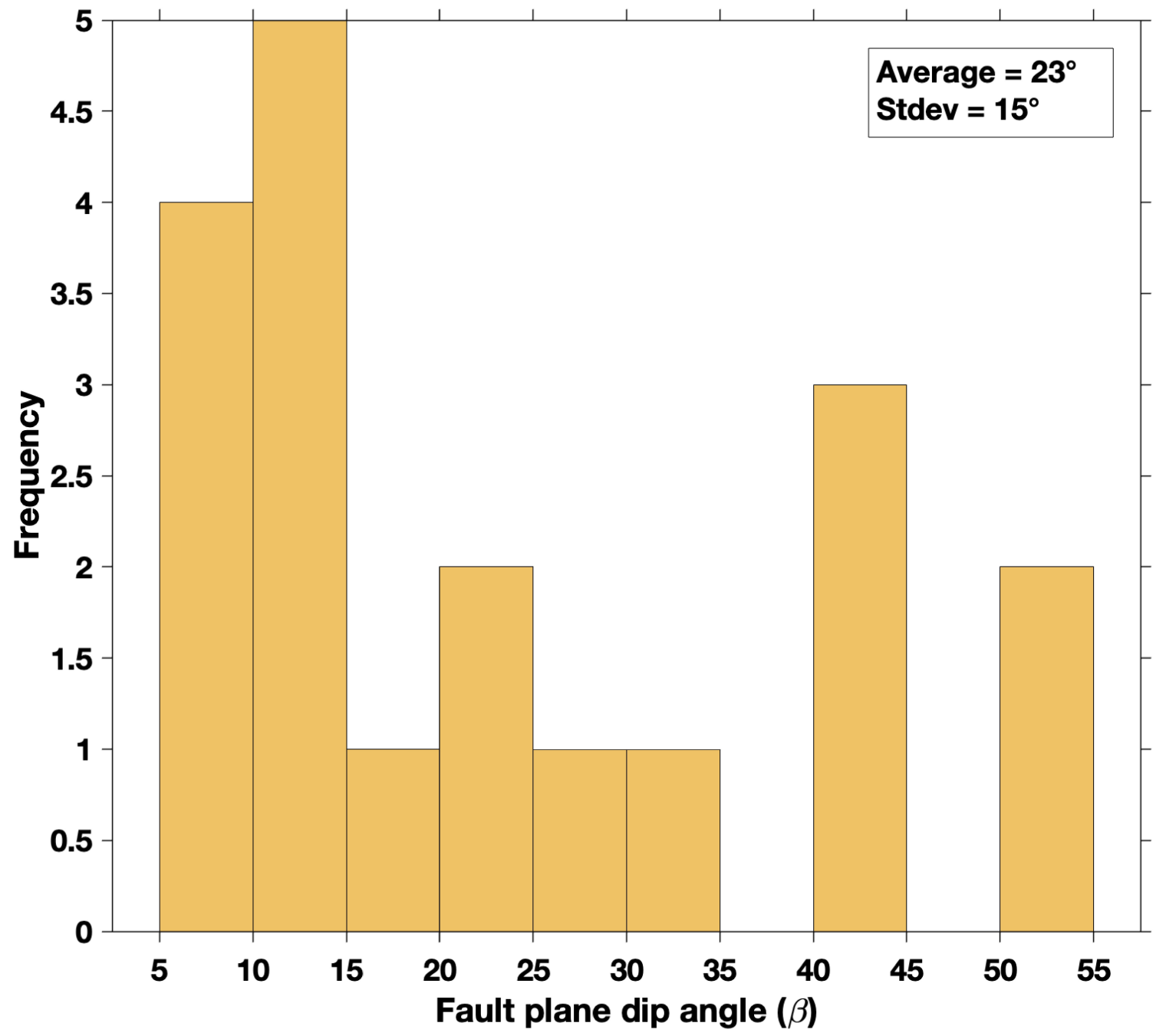

Supplementary Figure 1. **Frequency analysis of fault dip angles.** Frequency distribution of fault plane dip angles (β) for Mercurian thrust faults from [1, 2].

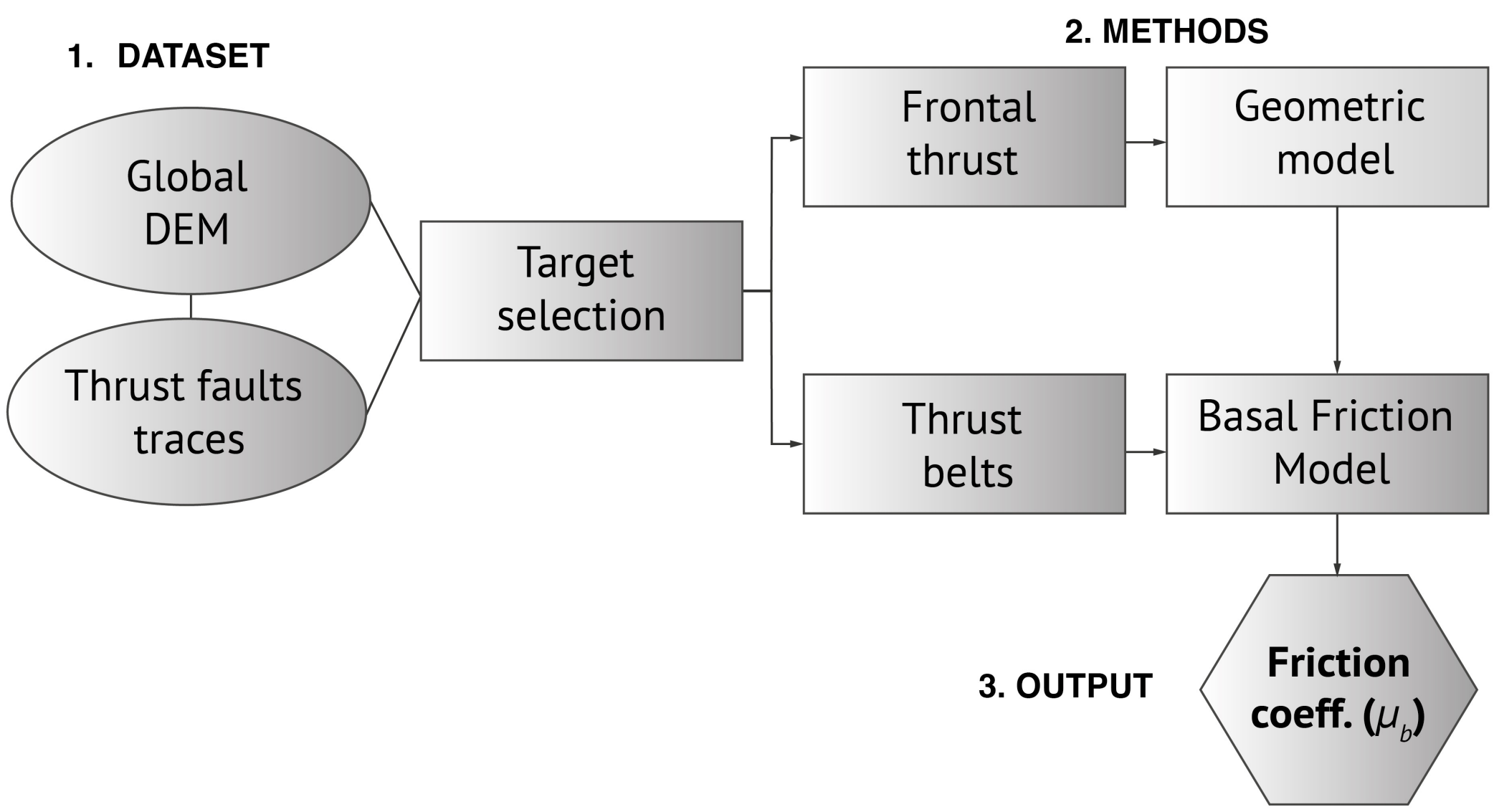

Supplementary Figure 2. **Basal-Friction Model (BFM) workflow.** Workflow of the Basal-Friction Model (BFM). Inputs (global 665 m/pixel DEM and mapped thrust fault traces) are combined during

target selection to identify fold-and-thrust belts (FTB) and their associated frontal thrust (FT). FTs provide geometric constraints (fault depth H) via the geometric model, while FTBs provide topographic constraints (α_{obs}) via the mechanical model. The obtained parameters are fed into the BFM which calculates μ_b using the Critical Taper equations (refer to Methods).

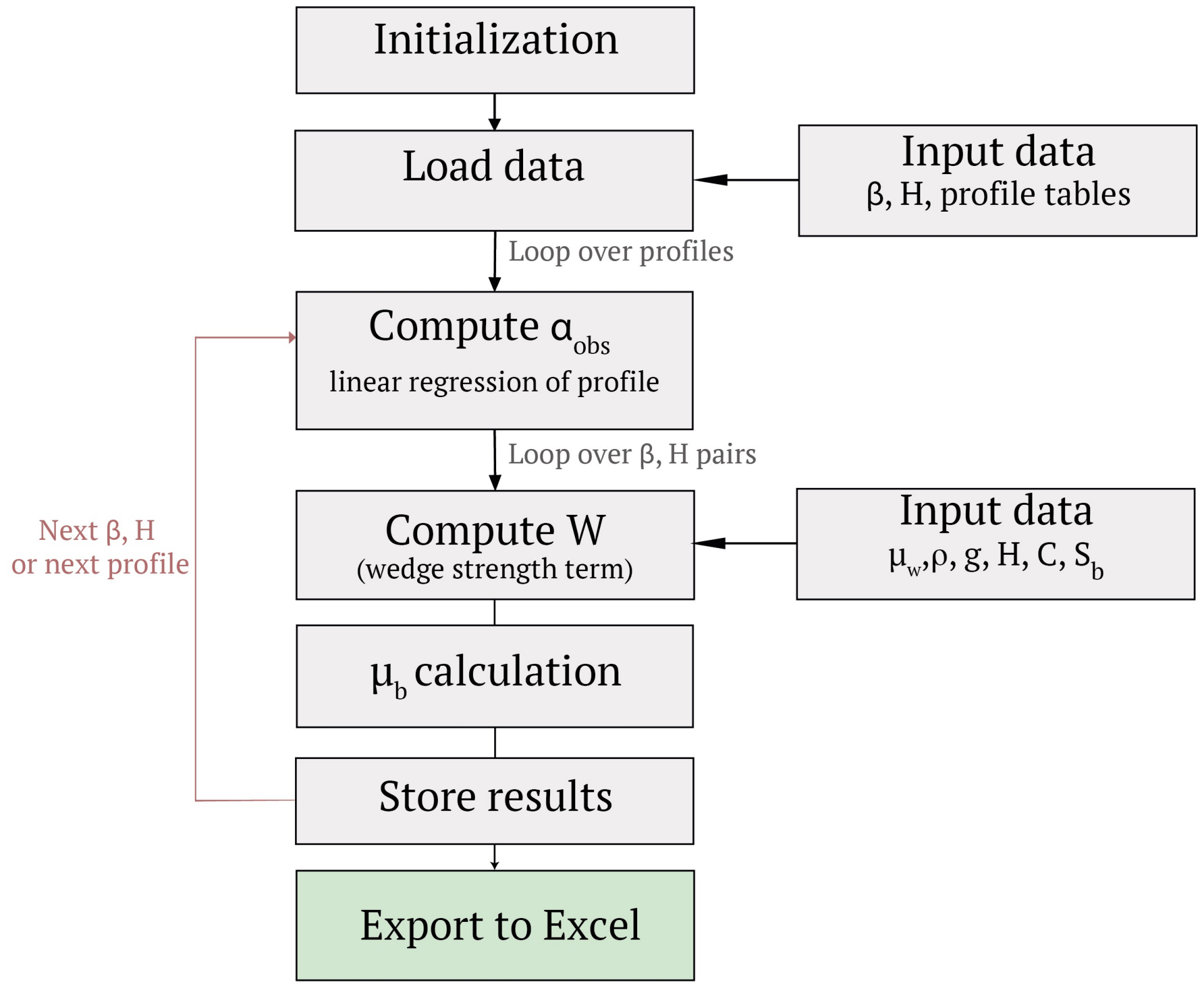

Supplementary Figure 3. **Pseudocode of the Basal-Friction Model (BFM)**. After initialization and data loading, the workflow iterates over topographic profiles to compute the observed wedge taper (α_{obs}) by linear regression, and over (β, H) pairs to compute the wedge strength term (W) and the basal friction coefficient (μ_b; Methods). Results are stored and exported to Excel.

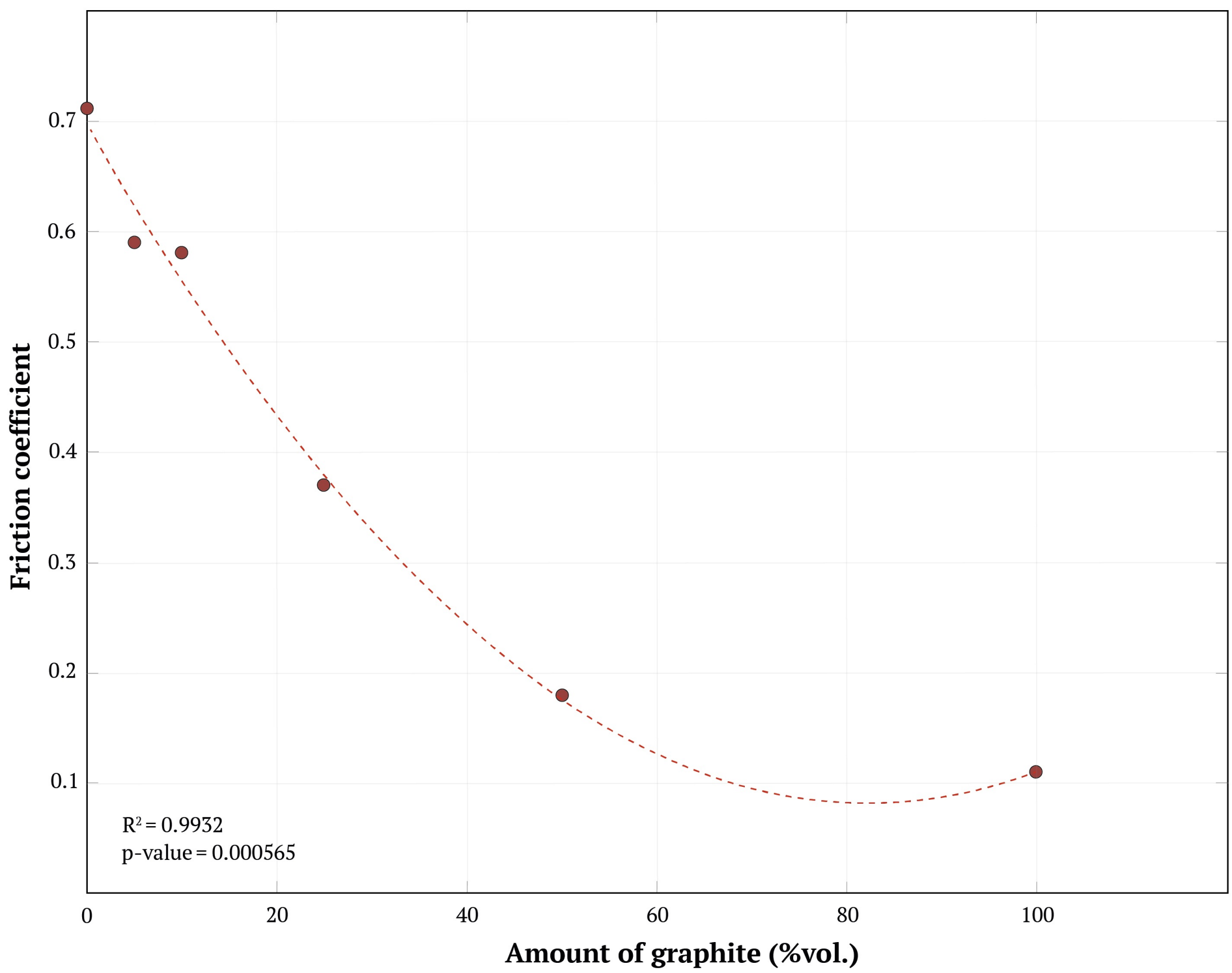

Supplementary Figure 4. **Variation of experimental friction coefficient with graphite content in basalt-graphite gouge mixtures.** Second-order polynomial fit through experimental friction coefficients at 0, 5, 10, 25, 50, and 100 vol% graphite, used to estimate the graphite content corresponding to $\mu_b \sim 0.25$ (~40 vol%).

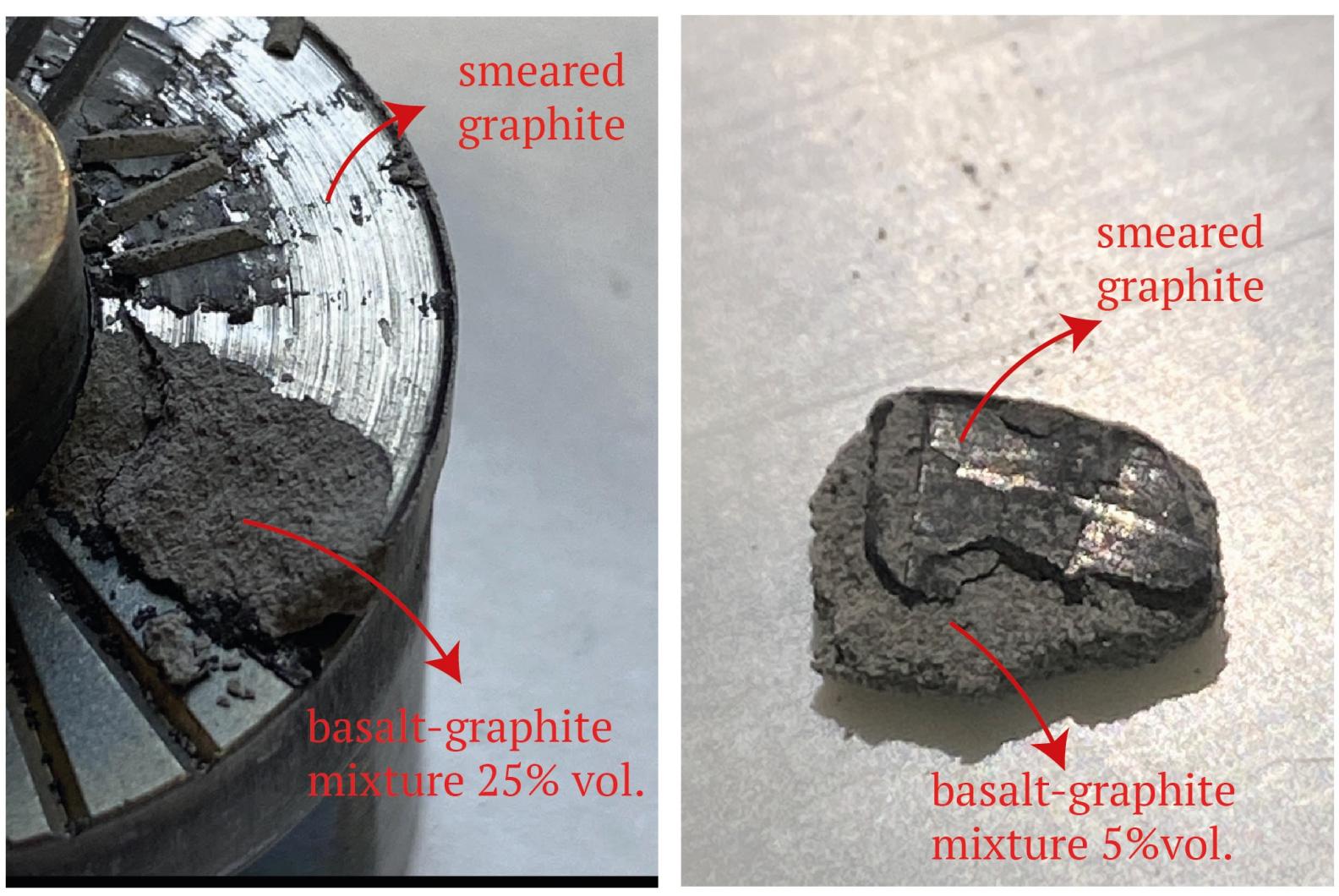

Supplementary Figure 5. **Graphite mechanical smearing.** Evidence of graphite mechanical smearing along the striated slip surface of experimental faults (reflective material), retrieved after

friction experiments in the Rotary shear apparatus. Similarly shiny, graphite-smeared slip surfaces are found in all experiments, regardless the initial graphite/basalt proportions. The left panel shows a gouge mixture containing 25% vol. graphite; the right panel shows a mixture containing 5%vol. graphite.

References

1. V. Galluzzi, G. Di Achille, L. Ferranti, C. Popa, P. Palumbo. Faulted craters as indicators for thrust motions on Mercury. Geological Society, London, Special Publications 2015 :313–325.

2. V. Galluzzi, L. Ferranti, M. Massironi, L. Giacomini, L. Guzzetta, P. Palumbo. Structural Analysis of the Victoria Quadrangle Fault Systems on Mercury: Timing, Geometries, Kinematics, and Relationship with the High-Mg Region. Journal of Geophysical Research: Planets 2019 :2543–2562.
