# Supplementary material for: Graphite lubricates Mercury’s global contraction
Source: Nat Commun. 2026 Aug 26;17:9063. doi: 10.1038/s41467-026-75459-x (PMC13518970; doi:10.1038/s41467-026-75459-x)
Supplement: Supplementary file 2 [file 41467_2026_75459_MOESM2_ESM.pdf]

nature portfolio

Peer Review File

Graphite lubricates Mercury's global contraction

Corresponding Author: Dr Natalia Vergara Sassarini

This file contains all reviewer reports in order by version, followed by all author rebuttals in order by version.

Version 0:

Reviewer comments:

Reviewer #1

(Remarks to the Author)
This manuscript presents an interesting and important topic; the global contraction estimate for Mercury is both very constraining and a topic of wide disagreement, yet estimates are still based on simplified models of the tectonics. A treatment of the surface expression of global contraction considering Mercury's unique, graphite-rich crust is welcome. However, I have several concerns with the manuscript as presented.

Major comment 1: The organization is hard to follow. Background, methods, and results are quite mixed. For example, it was not clear to me until line 138 that the authors actually did physical friction experiments, which seems quite important.

Major comment 2: The friction experiments require much more description and contextualization. What standards were used to validate the experimental setup? How were properties like porosity and temperature controlled? Results should be presented in more detail, and should be contextualized in terms of literature findings. Have there been other experiments on the influence of graphite on shear strength? How do the authors' results compare? How does the influence of graphite compare to the influence of grain size, temperature, or other important properties?

Major comment 3: It is not very clear in the manuscript whether weak basal detachments are an assumption or a finding. It seems to me that by beginning with shallow dip angles, there is an assumption of low strength, and that the major finding is that graphite can provide the coefficient of friction matching that low strength. But the paper is framed as if the major finding is that there is low strength. This should be clarified.

Major comment 4: I don't understand how the authors are applying the CT model to Mercury. A diagram would really help, as would a little more explanation. How does the crust of Mercury relate to the fault along which contraction is being accommodated, the folding of the upper portion and how that turns into a wedge, formation of asymmetry in the topographic profile, and to what specifically is the tectonics vocabulary used in the introduction referring?

Major comment 5: The description of the MIM confusing. What does it do beyond using equations 3-5 to calculate the coefficient of friction, given the input parameters specified in Extended Data Table 1? Phrases like "iterative model", "progressively refines", and "convergence" make it sound like it's doing a lot more, but I don't see this kind of thing in the code. If it is doing just a calculation based on equations 3-5, it should be described much more simply.

Major comment 6: the language requires editorial cleanup throughout. I have specified some but not all places in the minor comments.

Minor comments:

Line 12 – Secular cooling of the core was probably a main driver, but why exclude secular cooling of the mantle? Would this not have driven lobate scarp formation in the same manner?

Line 20 – "such layer" "such a layer", "impacts and gardening" redundant, can just be "impacts"

Line 21 – What is meant by the "entire" crust? There is certainly evidence of coherent graphite-poor regions, e.g. the thick volcanic units in the floor of Caloris.

Some words have unclear meaning. For example:
- Line 26: “representative”
- Line 35: “envision”
- Line 56: “architectures”

Line 28 – thermal contraction may certainly be dominant, but there should probably also be some volume change due to ongoing differentiation of the planet (inner core crystallization especially).

Line 29 – this should cite the various studies, and “according to different studies” is not necessary.

Punctuation with references is strange in places, e.g. line 33, 35, 36, etc.

Line 40 – justify the “only” plausible weak material; are there no others? Sulfide? Overpressure from volcanic degassing (Picasso hosts vents)? Impact-influenced materials? Or remove “only”; graphite is a very good candidate, but need not be the only possibility. The bulk of the results would apply equally to any weak material that might be discovered in the shallow layers of Mercury, why limit it?

Figure 1c is really not clear to me. Is this intended to illustrate a FTB? Why is there a rover in it? Where are the different layers that slip, and how are they bending/displacing?
Figure 1c: numbered equations should be present in text (repetition in figure is fine).
Figure 1d-1e: which transect is illustrated? H10 is a transect?

Line 75: “fall are in”

Line 74: How can crater deformation studies determine the fault dip angle? This seems worth a sentence of explanation given how fundamental the fault dip angle is to the conclusions of this paper.

Line 82: Why can low cohesion be assumed for the wedge and basal detachment?

Line 66-95: this seems like methods, not FTB mechanics as suggested by section heading.

Line 93-95: Existing fractures are assumed, not discovered, if I am understanding correctly. In this case, they cannot also be interpreted as they are in this sentence.

Line 110: missing word

Line 156: I believe both Peplowski and Klima find higher concentrations, locally.

Line 158-61: “most likely” and “the only way” require context. Which other materials were considered and are less likely or inconsistent with the observed deformation architecture?

Line 163: why would this grain size be representative of deep Mercurian crust? Lunar regolith is probably highly processed through impact gardening. There seems to be a hypothesized crust structure that is not really explained.

Lines 198-199: This seems like an over-interpretation: the results indicate a weakening agent, but cannot prove that it is graphite.

Line 241-242: Where is the importance of low-angle thrust faults in accommodating Mercury’s global contraction demonstrated?

Line 255-258: Why would shortening align with the higher estimates of Byrne et al. (2014)? Those were calculated with a (relatively) high dip angle of 25°.

Line 301: is there a unique relationship between the fault parameters and the basal friction coefficient? If not, how is non-uniqueness dealt with in the MIM?

Line 306: Authors mention their MIM distributes uncertainties related to DEM resolution: can this be explained further?

Line 311: a typo in the equation.

Line 313: Is it assumed that ? This seems implied by their definitions.

Line 316-317: How are the parameter values (cohesion parameters, density, etc.) chosen? How well are these known? How sensitive is to uncertainty in the input values?

(Remarks on code availability)
The code seems to carry out a series of simple calculations based on rows in an excel file. There is a step where several

values of mu_b are considered to see which produces a value for alpha matching the observed, but this seems to be a way of numerically solving equations (3)-(5) for the one unknown, mu_b.

Reviewer #2

(Remarks to the Author)
I have read the manuscript by Vergara Sassarini et al. titled: “Graphite lubricates Mercury’s global contraction”. The manuscript analyses 7 shortening landforms interpreted to be fold-and-thrust belts on Mercury as orogenic wedges using the critical taper approach. Paired with shear experiments using basalt-graphite mixtures, the results indicate that friction coefficients must be much lower than expected for basaltic rock alone, concluding that graphite must be facilitating slip on very low-angle thrust faults. Overall, this is an interesting (and somewhat higher impact) idea that is being tested in this research and the authors are commended for carrying out this detailed study. I really like the discussion on the confinement in narrow films along the fault plane and the required fault smear, both are concepts that we observe on faults on Earth but have not been considered for faults on Mercury. I have some overall concerns outlined below pertaining to (1) readability of the manuscript and (2) a wide applicability of the model to shortening landforms on Mercury and the interpretations arising from it. In its current format, the manuscript is not ready for publication in Nature Communications and I would personally prefer if the research were communicated in a longer format.

The manuscript is very condensed and arrives extremely quickly at the results. Important information that is needed to understand the logic of the research is reported elsewhere. In the presentation of the model and all following text, nearly every sentence refers the reader to another place in the manuscript or has additional information presented in parentheses. Both of these stylistic choices disrupt the flow of the logic and make it hard to read. In addition, Figure 1 is composed of at least 4 stand-alone figures, and therefore the figure caption is not detailed enough. Each of these figures should also be explained in more detail in the main manuscript text, as they are intended to get the point across of how many of the variables are derived. The symbols in the equations in this figure remain unexplained and the equation numbers are not the same as the ones in the Methods part. Together, this makes the description of the mechanical model disjointed. The manuscript would be much improved by separating Figure 1 into multiple figures and tying the methods section into the main text and have all aspects explained in logical order.

I have concerns about the overall applicability of the critical taper model. It is a model that was tested on 7 structures and as stated in the manuscript, not even all of them work. There are many other studies investigating the geometry/structural styles of hundreds of faults (Crane and Klimczak, 2019), derive dip angles and faulting depths/ thrust geometries (Petersen et al., 2020; Loveless et al., 2025). From this literature data, hundreds of structures were explained without the need to invoke graphite smear or a critical taper wedge. I have no doubt that the proposed mechanisms are indeed present at Mercury, but it remains unclear if this is widespread. I would regard it as an endmember process that is able to explain a few peculiar structures but isn’t necessarily representative of all of Mercury. I agree with the manuscript that all structures on Mercury should be assessed to identify if they require this mechanism and thus have accommodated more strain, but for now (because there is no case that a substantial number of faults have very low dips) the implications for global contraction are not dramatically changed.
Crane, K.T., Klimczak, C., 2019b. Tectonic patterns of shortening landforms in Mercury’s northern smooth plains. Icarus 317, 66–80. https://doi.org/10.1016/j.icarus.2018.05.034.
Peterson, G.A., Johnson, C.L., Byrne, P.K., Phillips, R.J., 2020. Fault structure and origin of compressional tectonic features within the smooth plains on Mercury. J. Geophys. Res.: Planets 125. https://doi.org/10.1029/2019je006183.
Loveless, S. R., Klimczak, C., Crane K. T., and Byrne, P. K., 2025, Geometric forward modeling of thrust systems underlying shortening landforms on Mercury. Journal of Structural Geology 198, 105449.

The manuscript has a few places where stress and strain terminology are inconsistently applied, I only list a few, but please check the issue throughout the manuscript. Compression refers to stresses. Normal faulting also requires compression, and so using the term “compression” when referring to structures formed by global contraction is misleading:
- Line 56: “Mercury’s major compressional architectures” should be “the morphology of shortening landforms on Mercury”
- Line 92: “compressional deformation” should be “contractional deformation”
- Line 94: “before any major tectonic compression” should be “before the onset of global contraction”

The extraction of thrust fault dips from faulted craters, its applicability to the interpretation of fault dips, and its application to the critical taper wedge needs a more complete discussion. The cited method detects the local dip at the very shallow portions of thrust faults. Geometric forward modeling using several techniques (not only the COULOMB code, but also MOVE) shows that dips may be variable along the fault. Faulting can be listric, indicating a shallowing of dip at depth. Fault geometries may also show a shallowing of dips at the near surface to accommodate the over-thrusting and folding at the surface. The dip issue becomes apparent when discussing Enterprise Rupes, where the model didn’t work so well. The dip discrepancies between the faulted crater method and the geometric forward modeling results at Enterprise Rupes were discussed in Loveless et al., 2025, which supports the findings discussed in line 133.

As an overall idea, does this research predict orogenic wedges on Mercury to be correlated with regions of increased LRM? Maybe this could be discussed as part of the schematic model (Figure 3).

Some Line-by-line comments:

Line 17: “we demonstrate that the observed topography of fold and thrust belts can be only fit by graphite bearing low angle fault slip zones” This is misleading, because the observed topography can only be fit by graphite bearing low angle fault slip

zones when using this particular model. Many thrust fault-related landforms can be explained with other thrust geometric models.

Line 18: “This indirectly supports models proposing the formation of a floating layer of graphite during Mercury’s early differentiation.” This is not true. The method here did not detect graphite on faults, it only invokes graphite. The model fits into the igneous evolution of Mercury.

Line 57: “crustal deformations and the Mercury’s early crust composition” should be “crustal deformation and the composition of Mercury’s early crust.
Line 65: Citation 33 is very general. The chapter should be specified.
Line 79 and elsewhere: What is the purpose of giving the quadrangle name? It is unclear to anyone unfamiliar with mapping Mercury. It is unexplained in the manuscript. It is fine just mentioning the name of the landforms.

Line 149: “Our analysis on Mercury fold and thrust belts shows that remnants of this layer must persist beneath the present upper crust” This is misleading. The analysis only shows that the utilized model requires a weakening lithology, and graphite is a plausible choice. As stated above, the model may not be universally applicable to all such landforms on Mercury. If a reader doesn’t consider this model applicable (of which I am sure there are some), then a remnant of this layer does not necessarily need to facilitate faulting.

Line 207 and Figure 3b: partial melting and volcanic resurfacing is not very obvious in the image.

Line 222: either remove “subsurface” or “at depth” as one of them is enough.

Figure 4 and associated discussion: the friction coefficient of 0.1 showing “this study” is misleading. This study really shows that pure graphite has a friction coefficient of 0.11 and the model solutions yielded a coefficient of 0.3 for ~25% graphite, as stated in line 220.

In addition, the statement in line 264: “Lower fault strength and shallower thrust faults lead to more efficient crustal shortening compared with stronger and steeper faults which result in less shortening” is incorrect (or the logic is reversed). The fault doesn’t cause shortening from global contraction. Shortening from global contraction causes the fault. It’s just that shallower thrust fault dips require more shortening to produce the observed topography when compared to the same topography produced by more steeply dipping thrust fault. Imagine Mercury undergoing a certain finite amount of global contraction, the same amount can be accommodated either by shallowly or steeply dipping thrusts. So instead of “lead to more efficient crustal shortening” I would put “require more shortening compared to more steeply dipping thrusts” and leave it at that.

Line 339: A noun is missing after “sheared”. Maybe “samples”?

I hope these comments and suggestions are helpful.
Regards, Christian Klimczak

(Remarks on code availability)
I have clicked through the code and visually inspected the structure, but I did not try to run it from my computer.

Version 1:

Reviewer comments:

Reviewer #1

(Remarks to the Author)
The manuscript organization and presentation have been very much improved. Overall, the manuscript is clear, well-illustrated, and quite readable. I maintain my opinion that the experiments are a useful contribution to the community, as is a quantification of the physical properties implied by particular fault dips. Furthermore, I appreciate the detailed responses to my previous comments, which have been largely addressed.

However, in reading the new manuscript, I have one major new concern regarding the calculation of basal friction; I believe the values calculated by the code do not match the equations presented in the manuscript, and that recalculation and possibly reinterpretation of the results and conclusions may be necessary.

I have provided a more detailed version of this comment as well as several more less major comments in the uploaded file.

[Editorial Note: See end of file for attachment]

(Remarks on code availability)
I have visually inspected the code, focusing particularly on the part calculating the basal friction coefficient. I have skimmed but not read in detail the portions which fit the transect parameters and read input files. I have not run the authors' code.

Reviewer #2

(Remarks to the Author)
I have read the revised version of the manuscript by Vergara Sassarini et al. titled: “Graphite lubricates Mercury’s global contraction”. The manuscript is much improved and reads well at this time. My previous comments were either incorporated into the revised manuscript and/or they were discussed in detail in the response letter. I have a few minor remarks for this version, but I think the manuscript is otherwise ready for publication.

Lines 40 and 46: I would reword the term “severely misoriented” when referring to low angle thrust faults. I would describe it as “inconsistent with Mohr-Coulomb theory in the context of Andersonian faulting”. It is probably not accurate to say “non-Andersonian faulting” (line 48) because low-angle thrust faults still form with horizontal sigma1 and so they are Andersonian. It is better to say “fault orientations deviating from orientations considered optimal for basaltic rock masses”.
For the map in Figure 2, please state projection and center of projection, so the reader would quickly be able to locate it in their own GIS.
Line 161 when referring to Loveless and Klimczak, 2025, it should be Loveless et al., 2025 (Reference 19).

(Remarks on code availability)

Version 2:

Reviewer comments:

Reviewer #3

(Remarks to the Author)
I am satisfied with the revisions of the manuscript in response to my previous comments and especially with the updates to correct the typo in the code and revise the text and discussion as necessary. I recommend publication. I thank the authors for their thorough responses.

(Remarks on code availability)
I have confirmed the update to the equations implemented. I have not otherwise inspected or run the code.

Open Access This Peer Review File is licensed under a Creative Commons Attribution 4.0 International License, which permits use, sharing, adaptation, distribution and reproduction in any medium or format, as long as you give appropriate credit to the original author(s) and the source, provide a link to the Creative Commons license, and indicate if changes were made.
In cases where reviewers are anonymous, credit should be given to 'Anonymous Referee' and the source.
The images or other third party material in this Peer Review File are included in the article’s Creative Commons license, unless indicated otherwise in a credit line to the material. If material is not included in the article’s Creative Commons

license and your intended use is not permitted by statutory regulation or exceeds the permitted use, you will need to obtain permission directly from the copyright holder.
To view a copy of this license, visit https://creativecommons.org/licenses/by/4.0/

Manuscript NCOMMS-25-45873-A

REPLY TO REVIEWER 1

Dear reviewer,

This letter accompanies the resubmission of the manuscript NCOMMS-25-45873-A.

We would like to sincerely thank you for your constructive criticism and thoughtful comments. Most of your suggestions have been addressed, and we believe that the changes have improved the quality, clarity and overall readability of the work.

Changes in the manuscript that directly address the reviewers' comments are highlighted in bright yellow, while additional modifications to the text are shown using track changes.

Point-by-point reply to Reviewer 1:

This manuscript presents an interesting and important topic; the global contraction estimate for Mercury is both very constraining and a topic of wide disagreement, yet estimates are still based on simplified models of the tectonics. A treatment of the surface expression of global contraction considering Mercury's unique, graphite-rich crust is welcome. However, I have several concerns with the manuscript as presented.

Major Comments Reply

1. The organization is hard to follow. Background, methods, and results are quite mixed. For example, it was not clear to me until line 138 that the authors actually did physical friction experiments, which seems quite important.

We appreciate this comment. In the first submission we had adopted a more "narrative" flow rather than follow the typical results-discussions structure. We now recognize this could confuse readers, and we have massively reorganized the manuscript to improve readability by: (i) establishing section titles (i.e., Results, Discussion, Methods) and improving the Results chapter readability by organizing our parameterization, modelling and experimental outputs into two sub-chapters; (ii) enriching the methodologies to avoid repetitions throughout the manuscript. You can see all these modifications in the new, revised version of the manuscript. In addition, we revised both the Abstract and the Introduction chapters to state up front that we performed friction experiments in parallel with the Critical Taper analysis.

Reviewed Abstract Line 15-17: "*Using Critical Taper analysis calibrated by friction experiments, we invert basal friction (μb) from observed wedge geometry and show that the topography of large fold-and-thrust belts is best explained by graphite-bearing, low-angle fault zones.*"

Reviewed Introduction Line 70-71: "*In this work, we employ the CT theory, constrained by friction experiments, to derive the friction coefficient of the basal detachment (μb) for thrust faults on Mercury*"

2. The friction experiments require much more description and contextualization. What standards were used to validate the experimental setup? How were properties like porosity and temperature controlled? Results should be presented in more detail and should be contextualized in terms of literature findings. Have there been other experiments on the influence of graphite on shear strength? How do the authors' results compare? How does the influence of graphite compare to the influence of grain size, temperature, or other important properties?

We agree the friction experiments needed clearer description. We revised the manuscript accordingly: we added concise methodological detail in the "Friction Experiments" subsection (Methods) and better contextualized the experimental results in the Results section. Below we address each of your sub-questions:

The friction experiments require much more description and contextualization. What standards were used to validate the experimental setup? How were properties like porosity and temperature controlled?

Experiments were carried out with the RoSA (Rotary Shear Apparatus) machine, hosted at the Department of Geosciences of University of Padova, fully described in Rempe et al., 2014. In the Methods section we added several details on the experimental apparatus. We note, however, that rotary shear testing is routine in rock mechanics and therefore the "setup" does not require validation, as it is not a new experimental procedure. Porosity is controlled through compaction in the machine (Vs. initial gouge volume) measured via LVDT and temperature was controlled via measurement with an internal thermocouple inserted in the sample holder.

Reviewed Methods Lines 383-398: "*We used the Rotary-Shear Apparatus (RoSA)*[87] *at the Department of Geosciences of Padua University (Padua, Italy) for the friction experiments. We sheared samples composed of basalt and graphite mixtures considered as representative endmembers of Mercury's basaltic crust/paleoregolith and of the most likely weak phase at depth, respectively.*

Samples are finely ground powders obtained by grinding in a disk mill and sieving down to grain size < 90 µm. Powders of each rock, basalt and graphite, were then mixed in four different proportions: 100% basalt (basaltic crust endmember), 75% basalt 25% graphite, 50% basalt and 100% graphite (primordial graphite layer). 4 grams of each mixture were then sandwiched between two grooved sample holders to form 3mm-thick layers that simulate the fault slip surface and mounted on the RoSA apparatus. Each test was carried out at a shearing velocity of 10 µm/s and imposing normal stresses (σn) from 5 to 20 MPa, under dry conditions and 25° C. Samples were sheared for 5 mm at each normal stress, until constant shear stress during dynamic frictional sliding was attained in each step. Data were collected at a frequency of 10 Hz. Details of the RoSA apparatus, sensor measurements and experimental procedures follow Rempe et al. 81. We measured the shear stress (τ) during stable sliding of the gouges at each normal stress and determined the average friction of each mixture by the slope of the linear Coulomb envelope."

Results should be presented in more detail, and should be contextualized in terms of literature findings.

As suggested, we added a more detailed sentence about the friction experiment results in the first Results sub-chapter, and compared our results with literature findings:

Reviewed Line 99-108: "*We directly tested the mechanical effect of graphite enrichment on mixtures of finely (< 90 µm) crushed basalt and graphite under normal stresses of 5-20 MPa, comparable to ~2 km of overburden on Mercury (Fig. 3a; Methods). Notably, the grain size of the simulated gouges overlaps the average size fraction of lunar regolith (i.e., ~70µm [46,47]) and, given the higher impact velocities on Mercury [48], is also a reasonable analogue for Mercurian paleoregolith. For each gouge composition, we measured the shear stress during steady-state sliding in the experimental fault at different imposed normal stresses. The average rocks coefficient of friction, computed from the slope of linear regressions in Mohr space (shear vs. normal stress; Fig. 3a), ranges from μ= 0.71 (pure basalt), to μ=0.37-0.18 (25% and 50% graphite, respectively), to μ=0.11 (pure graphite), consistent with refs. [31,35,49].***"**

Have there been other experiments on the influence of graphite on shear strength? How do the authors' results compare? The results of friction experiments are consistent with previous literature on graphite friction, which is a well-known low-friction material at a wide range of temperatures and pressures (e.g. Oohashi et al., 2011, 2013, and Rutter et al., 2013 already cited in line 110). The novelty of our experiments is the mixture of graphite with basalt gouges that are the analogue of

basaltic regolith (i.e. similar fine grain size and composition). These experiments on variable graphite-basalt mixtures allow to estimate the potential amount of graphite in Mercury's faults to explain fault weakness (discussed below).

How does the influence of graphite compare to the influence of grain size, temperature, or other important properties?

The influence of grain size and temperature, in dry conditions, are minor or negligible compared to the first-order weakening induced by the mixture with graphite. It is well-known from rock mechanics literature that diminishing grain size has the general effect of a small increase in friction, whereas bulk friction is largely insensitive to temperature, up to temperatures close to the brittle-ductile transition, especially at the dry conditions expected on Mercury (e.g. Rabinowicz, 1965; Blampied et al., 1995; Scholz, 2019 and references therein).

3. It is not very clear in the manuscript whether weak basal detachments are an assumption or a finding. It seems to me that by beginning with shallow dip angles, there is an assumption of low strength, and that the major finding is that graphite can provide the coefficient of friction matching that low strength. But the paper is framed as if the major finding is that there is low strength. This should be clarified.

The manuscript shows that some most prominent lobate scarps on Mercury are compatible with low angle detachment thrusts whose dip angles has been directly measured in their frontal part (Galluzzi et al., 2015, 2019). This faulting is only possible along weak fault plains that within Mercury's crust can be obtained by graphite reach horizons. The needed low friction value was obtained through shear experiments on mixtures containing at least 25% of graphite (see Figure 3a). Thus, the main findings of the manuscript are: (i) The subsurface detachment dip angles able to explain some of the observed, most prominent lobate scarps and fold-and-thrust belts on Mercury surface assuming critical taper geometries; (ii) The friction coefficient values allowing slip along such low-angle detachments; (iii) The graphite content able to achieve such low friction coefficients through shear experiments. To clarify these findings, we have modified the text within the manuscript (see example below) (see also our reply to Reviewer 2, comment 2 C, D).

About low-angle faults:

Reviewed Line 35-39: "*Other recent investigations of the most prominent lobate scarps* [14–18] *indicate that they can be interpreted as fold-and-thrust belts (FTBs*[7]*) and suggest the presence of regionally continuous low-angle thrust faults associated with hanging-wall anticlines. Indeed, low-angle thrusts*

associated with several lobate scarps have been directly derived from deformed craters [15,16] *and modeling* [19]*."*

About this work findings:

***Reviewed Abstract Lines 15-24**: "Using Critical Taper analysis calibrated by friction experiments, we invert basal friction (μb) from observed wedge geometry and show that the topography of large fold-and-thrust belts is best explained by graphite-bearing, low-angle fault zones. This finding indirectly supports models proposing a graphite flotation crust during Mercury's early differentiation. We infer that remnants of this carbon-rich layer, heterogeneously distributed within the Hermean crust because of early impacts, caused the weakening of large portions of the crust during later contraction, promoting the nucleation of low-angle thrust faults. Since the gently dipping, frictionally weak thrusts require greater horizontal shortening to build the observed relief than steeper, stronger faults, Mercury's cumulative radial contraction may exceed previous, more conservative estimates."*

***Reviewed Introduction Lines 73-75: "**Our approach highlights the crucial role of graphite-bearing layers in controlling the geometry and mechanics of Mercury's major shortening landforms, linking surface expressions of crustal deformation to the composition of Mercury's early crust."*

***Reviewed Lines 217-220:** "In this context, our modeling and experiments indicate that graphite-rich material along low-angle anisotropies is the most plausible weakening agent for the low friction coefficients inferred along Mercury's thrust detachments, and together they provide a mechanically consistent explanation for the observed contractional landforms."*

***Reviewed Lines 277-281:** "Our work suggests that graphite smearing along crustal fault planes provides a robust explanation for the geometry and kinematics of the large-scale fold-and-thrust belts observed today on Mercury's surface, which would otherwise be mechanically unconceivable. Consequently, Mercury's early magma ocean differentiation led to a unique, graphite-dominated global tectonic regime in the Solar System, demonstrating how critically early planetary evolution controls later crustal mechanics."*

4. I don't understand how the authors are applying the CT model to Mercury. A diagram would really help, as would a little more explanation. How does the crust of Mercury relate to the fault along which contraction is being accommodated, the folding of the upper portion and how that turns into a wedge,

formation of asymmetry in the topographic profile, and to what specifically is the tectonics vocabulary used in the introduction referring?

In the revised manuscript, we split the former Figure 1 into two figures. The new Figure 2 clarifies how lobate-scarp topography relates to the critical Taper model by explicitly marking the basal detachment, the active thrust, and back-thrust surfaces to illustrate wedge growth. We have standardized the terminology used in the Introduction paragraph to distinguish stress (compressional) from strain/structures (contractional/shortening), replaced ambiguous terms (e.g., 'architectures'→ geometries), and defined all symbols at first use. **See also our reply to Reviewer #2, point 3**.

5. The description of the MIM is confusing. What does it do beyond using equations 3-5 to calculate the coefficient of friction, given the input parameters specified in Extended Data Table 1? Phrases like "iterative model", "progressively refines", and "convergence" make it sound like it's doing a lot more, but I don't see this kind of thing in the code. If it is doing just a calculation based on equations 3-5, it should be described much more simply.

We thank the reviewer for highlighting this point. We have dropped the term MIM (and language like "iterative", and "progressively refines") because could be misleading. We now describe the method as a Critical Taper basal-friction inversion and refer to it as the **Basal-Friction Inversion Model (BFIM)**. In practice, BFIM performs a discrete parameters inversion using the simplified Critical Taper equations (Suppe, 2007). For each profile we fix the depth (H), derived from the wedge thickness calculated through the geometry analysis, the observed topographic angle (α_{obs}), and the material parameters: coefficient of friction of the wedge (μ_w) derived from our experiments; wedge and basal cohesion (C, S_b) derived from shear experiments and literature. We also use a range of fault dips (β) derived from Galluzzi et al. (2015, 2019) measurements. The model assumes dry and airless conditions (pore fluid pressure ratio in the wedge $\lambda \approx 0$, with a pore fluid pressure $P_f \approx 0$). We sweep $\mu_b \in [0.05, 1.00]$ with $\Delta = 0.01$ at $\beta \in \{5°, 10°, 15°, 20°\}$; compute a topographic angle α_{calc}; and accept solutions where $|\alpha_{calc} - \alpha_{obs}| \leq 0.1°$. Lack of a solution at a given β is interpreted as a dip constraint (e.g., $\beta > 5°$). The Methods section has been revised accordingly to reflect this parameter inversion and to avoid overstating complexity.

Reviewed Lines 347-364: "*The Basal-Friction Inversion Model (BFIM), implemented in MATLAB, relies on the Critical Taper theory and uses the simplified relations from Suppe (2007) based on the weak-base theory of Dahlen, 1990* [40] *(Eqs. 3-5). BFIM performs a discrete parameter inversion for the basal friction coefficient* (μ_b)*. For each FTB cross-section we fix the detachment depth H (from*

geometric analysis; Methods, Geometric Model), the observed topographic angle (α_{obs}), and the material parameters. The latter are the coefficient of friction of the wedge (μ_w) derived from our experiments, and the wedge and basal cohesions C and S_b (0.5 MPa each) derived from shear experiments and supported by literature[50–52]. We also explore fault dips (β) from independent measurements by Galluzzi et al. (2015, 2019). In the equations below, F and W represent the nondimensional décollement and wedge strengths, respectively; $\varphi = \arctan(\mu_w)$ is the internal friction angle; g is the acceleration due to gravity (3.7 m/s^2) [83,84]; ρ is the average density of the wedge material (3340 kg/m^3; e.g. [29,85]); μ_b is the basal coefficient of friction, ρ_f is the density of the overlying fluid (≈ 0 here); λ is the pore-fluid pressure ratio; and C and S_b are the cohesion terms (0.5 MPa). The model assumes airless, dry conditions with negligible pore-fluid pressure ($P_f \approx 0$ so the pore-fluid pressure ratio $\lambda = P_f/\rho g H \approx 0$ and Eqs. 3-4 reduce to 6-7) and no overlying fluid, ($\rho_f \approx 0$, hence $\rho_f/\rho \approx 0$ and Eq. 5 reduces to Eq. 8). We sweep $\mu_b \in [0.05, 1.00]$ with $\Delta\mu_b = 0.01$ for $\beta \in \{5°, 10°, 15°\}$ to compute a topographic angle α_{calc} and accept solutions where $|\alpha_{calc} - \alpha_{obs}| \leq 0.1°$, reporting accepted μ_b values and goodness-of-fit metrics (RMSE, R^2; Extended Data, Fig. 2).” See equations from 3 to 8 in the revised manuscript text.

6. The language requires editorial cleanup throughout. I have specified some but not all places in the minor comments.

We have thoroughly revised the manuscript to improve clarity and structure and implemented your in-line suggestions. We have also corrected citation punctuation.

Line-by-line Comments Reply

Line 12 – Secular cooling of the core was probably a main driver, but why exclude secular cooling of the mantle? Would this not have driven lobate scarp formation in the same manner?

We agree that mantle cooling also contributes. Consistent with Tosi et al. (2013), both the core (likely the dominant contributor) and the mantle drive bulk volume loss. We revised the text accordingly:

Reviewed **Line 12-13**: "*Secular cooling of Mercury's interior drove planetary contraction, forming widespread lobate scarps…*"

In addition, we add details to **reviewed Lines 28-31** in the **Introduction** chapter: "*They vary in length from tens to hundreds of kilometers* [1,2] *and are interpreted as the surface expressions of thrust faults that accommodate planetary contraction driven primarily by secular cooling of Mercury's core and mantle* [3,4]. *The estimated decrease of planetary radius widely ranges between 1-3 km* [5,6] *and 7 km* [7] *(Fig. 1).* "

Line 20 – "such layer" "such a layer", "impacts and gardening" redundant, can just be "impacts"

We have modified the text within the manuscript.

Reviewed Line 19: "*…remnants of this carbon-rich layer…*"

Line 21 – What is meant by the "entire" crust? There is certainly evidence of coherent graphite-poor regions, e.g. the thick volcanic units in the floor of Caloris.

We agree that "entire crust" was overstated. We revised the manuscript text accordingly:

Reviewed Line 19-21: "*We infer that remnants of this carbon-rich layer, heterogeneously distributed within the Hermean crust because of early impacts, caused the weakening of large portions of the crust during later contraction, promoting the nucleation of low-angle thrust faults.*"

Some words have unclear meaning. For example:

We have systematically revised the manuscript, in addition to the specific changes detailed below:

- **Line 26**: "representative"

Reviewed Line 27: "*Lobate scarps are Mercury's **most characteristic** and widely distributed contractional tectonic features*."

- **Line 35**: "envision"

Reviewed Line 36: "*Other recent investigations of the most prominent lobate scarps* [14–18] ***indicate that they can be interpreted** as fold-and-thrust belts (FTBs;* [7]*)…*"

- **Line 56**: "architectures"

Reviewed Lines 73-75: "*Our approach highlights the crucial role of graphite-bearing layers in controlling the geometry and mechanics of Mercury's major **shortening landforms***"

Line 28 – thermal contraction may certainly be dominant, but there should probably also be some volume change due to ongoing differentiation of the planet (inner core crystallization especially).

We agree that compositional contraction from inner core crystallization can contribute. However, Tosi et al. (2013) neglect this term in their radius calculation, arguing that with sufficient light elements (e.g., S) the inner core growth starts toward the end of the evolution and does not contribute significantly to the global radius balance, as previously discussed in Grott et al., (2011). In this context, the main contributions to global contraction are given by volume changes associated with thermal contraction of the core and mantle, with additional effects from partial melting and accompanying differentiation. However, since we are not directly calculating radial shortening (and our CT inversion is independent of the specific driver of contraction) nor discussing the mechanisms that drove thermal contraction, in the revised version of the manuscript we describe contraction as due to secular cooling of the core and mantle citing Tosi et al. (2013), which we think is sufficient for the scope of this work.

See our previous response to **Line 12**.

Line 29 – this should cite the various studies, and "according to different studies" is not necessary.

We have modified the text accordingly in the new Lines 28-31:

Reviewed Line 28-31: "*They vary in length from tens to hundreds of kilometers [1,2] and are interpreted as the surface expressions of thrust faults that accommodate planetary contraction driven primarily by secular cooling of Mercury's core and mantle [3,4]. The estimated decrease of planetary radius widely ranges between 1-3 km [5,6] and 7 km [7] (Fig. 1).*"

Punctuation with references is strange in places, e.g. line 33, 35, 36, etc.

We used the commas to indicate separate individual citations (e.g., 1,3,6), and an end dash to indicate a range of citations (e.g., 1-6). This notation is consistent with the Nature referencing style scheme, according to Nature Communications guidelines for citation (https://www.nature.com/ncomms/submit/how-to-submit). We decided to keep this formatting since Zotero assigns citation numbers automatically, and manually editing punctuation could break the numbering consistency across the manuscript. We are of course willing to adapt the punctuation style if the editor prefers.

Line 40 – justify the "only" plausible weak material; are there no others? Sulfide? Overpressure from volcanic degassing (Picasso hosts vents)? Impact-influenced materials? Or remove "only"; graphite

is a very good candidate but need not be the only possibility. The bulk of the results would apply equally to any weak material that might be discovered in the shallow layers of Mercury, why limit it?

Response to Line 40 (this comment) and Lines 158-61 (comment below):

Thank you for this helpful point. We address both comments together because they raise the same issue. We agree that, in general, we need more context to justify graphite as the most plausible weak material. In this context, in the manuscript (Introduction and Results) we now (1) explicitly evaluate alternative weak mechanisms in a Mercury-specific framework, and (2) replace "most likely / only way" with "most consistent" and "plausible endmember."

Below we summarize the reasoning about the different potential fault weakening factors:

- **Evaporites**. Thick halite décollements on Earth require long-lived brines and a hydrologic cycle. Mercury lacks a substantial atmosphere/hydrosphere; water is confined as ice in permanently shadowed regions at the poles (Chabot et al., 2012), with no evidence for evaporitic basins or salt tectonics at global scale.
- **Phyllosilicate-rich gouges**. Weak clay-rich gouges on Earth generally form via aqueous alteration (e.g., diagenesis/hydrothermal processes; Meunier, 2005). Orbital spectroscopy at Mercury shows no widespread hydrated phases (Inzenberg et al., 2014).
- **Fluid overpressure (and sulfides)**. Elevated pore-fluid ratios (λ) weaken many terrestrial wedges, but long-term, regional overpressure is unlikely on Mercury's anhydrous, impact-fractured crust with no hydrologic cycle. We therefore adopt dry conditions (λ≈0) in our CT inversions. Although pyroclastic vents and faculae document volatile release (Kerber et al., 2011; Pegg et al., 2021), these are localized and episodic, not at basin-scale and unlikely sustained pressure sources over geologic time (Tosi et al., 2013; Giacomini et al., 2020). Moreover, the explosive activity that emplaced pyroclastic deposits is relatively recent spanning from ~3.9 Ga until less than a 1Ga (Thomas et al., 2014; Byrne et al., 2016). Therefore, volatile release recorded by vents and faculae post-dates the main phase of FTB formation, which most likely ended around 3.6–3.8 Ga (Giacomini et al., 2020), and could not have sustained long-lived, basin-scale overpressure during their growth. Finally, sulfides are associated with local volatile release, such as hollows (Barraud et al., 2023) and pyroclastic deposits (Lucchetti et al., 2011). Although MESSENGER observations indicate that sulfur (Ca-rich and Mg-rich sulfides) is present on Mercury (e.g., Nittler et al., 2011; Weider et al., 2012; Evans et al., 2012), it is unlikely that these recent and localized phenomena could have facilitated the formation of regional scale detachments.

- **Sulfide or impact-modified materials**. To our knowledge, sulfides are not documented to act as primary weakening agents in Earth fault gouges; therefore, we did not discuss them in the main text. In addition, as we stated in the previous sentence, sulfides are associated with local volatile release, such as hollows (Barraud et al., 2023) and pyroclastic deposits (Lucchetti et al., 2011). Although MESSENGER observations indicate that sulfur (Ca-rich and Mg-rich sulfides) is present on Mercury (e.g., Nittler et al., 2011; Weider et al., 2012; Evans et al., 2012), it is unlikely that these recent and localized phenomena could have facilitated the formation of regional scale detachments.

- **Graphite**. Graphite aligns with (i) reducing differentiation and the proposed graphite flotation crust, (ii) remote sensing of carbon-rich low-reflectance material (LRM) excavated from depth, and (iii) low-friction behavior in laboratory studies (including ours). Graphite can also localize as thin smeared films along slip zones, avoiding the need for volumetrically abundant carbon in the bulk crust.

Accordingly, the sentence in **Line 40** was rewritten as:

Reviewed Line 58: "*Graphite can greatly reduce friction in fault zones [35–39], making it a strong candidate for facilitating slip in Mercurian faults.*"

Then, we added more context after **reviewed Lines 178-194**: "*Evaporitic minerals require persistent surface (or near-surface) brines and fluid circulation to accumulate, and phyllosilicates (e.g., clay minerals and micas) are either associated with siliciclastic sedimentation in aqueous environments or with diagenesis and hydrothermal alteration[22]. Such conditions are not expected on Mercury, where liquid water can only be stable as ice within the permanent shadowed regions [61]. In addition, global spectral data show no diagnostic ~1.4 µm absorption bands typical of hydrated minerals (including phyllosilicates and evaporites) [62]. Long-term, regionally elevated pore-fluid pressures are difficult to sustain in a highly fractured, impact-disrupted crust with no long-lived hydrologic cycle. Although explosive volcanism and volatile-driven degassing are documented by the presence of pyroclastic vents [63–65]and faculae [66,67], such activities are spatially limited and transient, and are unlikely to maintain elevated pore pressures on scales of hundreds to thousands of kilometers over the ~100 Myr timescales corresponding to the lifespan of large FTBs [4,53]. Moreover, the explosive*

activity that emplaced pyroclastic deposits is relatively recent, spanning ~3.9 Ga to < 1 Ga [68,69]*. Therefore, the volatile release recorded by vents and faculae postdates the main phase of FTB formation, which most likely ended around 3.6–3.8 Ga* [53]*, and could not have sustained long-lived, regional overpressure during their activity. By contrast, the existence of a graphite-enriched layer is particularly likely, since it may have constituted a primary graphite flotation crust during the early stages of the planet's formation* [27,33]*.*"

In addition, we mention this clearly in the **Introduction, reviewed Lines 43-49**: "*On Earth, weak layers are commonly constituted by bodies of salt, sulphates- or phyllosilicate-rich rocks with a low friction coefficient (μ < 0.25; e.g.,* [21,23–26]*) and often associated with elevated fluid pressure. However, fluid pressure acts isotropically and does not favor misoriented faults over optimally oriented ones, whereas low-angle layers and anisotropies associated with weak materials are pivotal for non-Andersonian faulting. On Mercury, long-term regional fluid overpressure and widespread hydrated weak rocks are not expected because of its anhydrous crust lacking any hydrologic cycle.*"

Figure 1:

Figure 1c: is really not clear to me. Is this intended to illustrate a FTB? Why is there a rover in it? Where are the different layers that slip, and how are they bending/displacing?

We have now revised Figure 1c to improve clarity; it is now presented as **Figure 2b**. This panel shows a simplified illustration (cartoon) of the Critical Taper depicted as the self-similar growth of a "bulldozer wedge", as presented by several authors including Dahlen (1990), Dahlen and Suppe (1984), Bilotti and Shawn (2005), Yang et al. (2016), Ring et al. (2019) for Earth, and Nahm et al. (2010) for Mars as well as in Structural Geology textbooks (Fossen, 2016).

The rover is used as a light adaptation of the traditional "bulldozer" icon that is commonly shown on Earth-based critical wedge illustrations; here, we replaced the bulldozer with a rover to emphasize the planetary setting. We can replace it with a neutral arrow if the editor prefers. In addition, **Figure 2c** now explicitly labels the basal detachment, the active thrust and, back-thrust within the wedge, and we added bigger displacement arrows to illustrate slip and folding.

Figure 1c: numbered equations should be present in text (repetition in figure is fine).

The equations shown in the Figure 1c (**now Figure 2 b**) are now thoroughly explained in the Methodology section. In the new Figure 2b, the CT modelling parameters are shown and explained both in the main text and the caption. We ensured consistent notation between the figure and the

Methodology text, and we decided to not include the equations within the figure, since they're thoroughly explained in the methodology chapter "Mechanical Model".

Figure 1d-1e: which transect is illustrated? H10 is a transect?

Figures 1d and 1e (**now Figure 2 c, d**) illustrate the average topographic profiles obtained from multiple transects traced across the main Fold-and-Thrust Belt (FTB) and the associated frontal thrust in the H10 (Picasso fault system) target area (see panel in the **new Figure 2 for** reference). For each selected target, including H10, several transects were drawn in the vergence direction across the FTB and isolated thrusts using the Qprof tool in QGIS. The profiles shown in panels **2c-2d** represent the mean profiles derived from these transects, and H10 is used here as a representative example. We have modified the figure caption to clarify this point.

Line 75: "fall are in"

Reviewed Line 93: "*…yielding typical dip angles between ~5° and ~15°"*

Line 74: How can crater deformation studies determine the fault dip angle? This seems worth a sentence of explanation given how fundamental the fault dip angle is to the conclusions of this paper.

Thanks for this helpful comment. We agree that this statement could benefit from a brief explanation. Galluzzi et al. (2015) estimate thrust fault dip by treating impact craters as passive strain markers (analogous to kinematic indicators used on Earth). The relative displacement across a thrust fault cutting a crater, produces characteristic vertical and horizontal offsets between the rims in its hangingwall and the footwall. By measuring those displacements on images and DTMs (digital terrain models) and fitting a simple thrust-fault geometry (i.e. a planar thrust), they solve for the slip and dip of the underlying fault often finding dips <20°. To clarify this point, we added a brief explanation within the manuscript.

Reviewed Line 89-93: "*Independent constraints on thrust geometry, specifically the basal dip (ß) of the studied thrust faults, come from crater-deformation studies which treat faulted impact craters as passive strain markers[15,16]. In those cases, vertical rim offset, and asymmetric hanging-wall uplift measured in digital terrain models (DTMs) are used to reconstruct the geometry of the shallow portion of the thrust geometry, yielding typical dip angles between ~5° and ~15° (Extended Data, Fig. 1)."*

Line 82: Why can low cohesion be assumed for the wedge and basal detachment?

In the revised manuscript, within the "**Fold-and-thrust belts geometry and mechanical characterization**" section, we made now clear that the cohesion values are (i) estimated from rotary shear results and (ii) confirmed by previous works. The revised version of the manuscript is now as follows:

Reviewed Lines 109-112: "*and adopted low cohesive strengths of 0.5 MPa for both the wedge (C) and the detachment (S_b). These values are experimentally supported by the rotary shear results (i.e., cohesion of basalt and basalt-graphite mixtures is < 1MPa; Fig. 3a) and are consistent with previous work indicating that Mercury's crust was already heavily fractured before the onset of global contraction* [50–52]*.*"

Our reasoning is that cohesion values for intact competent basalt can be significantly higher (from a few MPa up to tens of MPa) (Bieniawski and Bieniawski, 1989; Schulz, 1993) but such values are not representative of a heavily damaged, impact-processed and thermally stressed crust. Indeed repeated impact fracturing, cooling-related cracking of lavas, and global thermal stresses are thought to have substantially reduced crustal strength well before contractional tectonics initiated (Bieniawski and Bieniawski, 1989; Klimczak 2015), in a way analogous to the intense upper-crustal damage documented on the Moon (Carrier, 1973). For this reason, we adopt a low effective cohesion (0.5 MPa) as a boundary condition, rather than the cohesion of an intact, unfractured rock mass.

We also address this in the Discussion **Reviewed Lines 201-206**: "*This implies that deformation in Mercury's thrust fault systems localized within pre-fractured, megaregolith-like material rather than intact basalt, suggesting that the crust strength was significantly reduced before any major tectonic compression. Within this framework, the frictional behavior of pure basalt in our experiments represents that of a damaged basaltic crust interleaved with paleoregolith layers and transected by inherited anisotropies underlined by fine gouges*"

Line 66-95: this seems like methods, not FTB mechanics as suggested by section heading. We appreciate this point and we agree that, in the original submission, methods (see the revised "**Fold-and-thrust belt characterization**" section) and results were interleaved in a way that made the section hard to follow. In the revised manuscript we have separated the two topics much more

effectively. The full workflow of the BFIM, the Critical Taper formulation, and the inversion method used to derive μ_b, together with a more detailed description of the friction experiments appears in the Methods section (at the end of the manuscript for NCOMMS journal). What remains in the manuscript under "**Fold-and-thrust belts geometry and mechanical characterization**" is now limited to the observational and experimentally derived constraints that feed the inversion, which we consider results. So, the measured topographic angle, the range of detachment dips, the estimated depths, the internal friction of the wedge and the cohesion are quantitative constraints derived from topography, structural measurements, and our rotary shear experiments. We view them as key geological findings about the geometry and mechanics of the studied FTBs.

Line 93-95: Existing fractures are assumed, not discovered, if I am understanding correctly. In this case, they cannot also be interpreted as they are in this sentence.

We agree that, in the original text, the wording could be read as if we had directly observed pre-existing fractures in Mercury's crust from our own dataset. In the revised version, we no longer present the Mercury fractured crust as "detected" in our work, but explicitly as a assumed condition supported by published studies: repeated impact fracturing, cooling-related cracking of lavas, and global thermal stresses are all expected to have significantly weakened Mercury's upper crust before large-scale contraction began (Schulz et al., 1993; Klimczak et al., 2015). We now state that a low effective cohesion (0.5 MPa) was adopted to reflect the previously damaged state of the crust. This clarification is incorporated in the revised Results section within the text (see our reply to comment **Line 82**).

Line 110: missing word

This line was corrected in the revised manuscript new **Line 138**

Line 156: I believe both Peplowski and Klima find higher concentrations, locally.

Revised Lines 54-57: *"…are thought to be particularly enriched in graphite, with modeled global near-surface abundances on the order of 1 wt.%*[27]*, locally enriched up to ~3-4 wt%. This material is thought to be derived from a graphite-rich flotation crust formed during the early stages of crustal differentiation and subsequently excavated by large impacts* [27,32–34]*.*"

Line 158-61: "most likely" and "the only way" require context. Which other materials were considered and are less likely or inconsistent with the observed deformation architecture?

Thank you again for this helpful point. In the manuscript we now (1) explicitly evaluate alternative weak mechanisms in a Mercury-specific framework (see response to **LINE 40**).

Then, we replaced "most likely / only way" with "suggesting that" and "successfully reproduce", as shown in the reviewed text below:

Reviewed Lines 164-168: *"Despite these steeper angles, the friction coefficients (μ_b) remain among the lowest in our dataset (Fig. 3b), suggesting that a weakening material played a role in facilitating fault slip in both systems. We then compare these μ_b values inferred for the basal detachments of our case studies with the results of the rotary-shear friction experiments (Fig. 3a). Experimental results show that fault gouges with graphite enrichments on the order of ~25 vol% successfully reproduce the modeled weak thrusts."*

Line 163: why would this grain size be representative of deep Mercurian crust? Lunar regolith is probably highly processed through impact gardening. There seems to be a hypothesized crust structure that is not really explained.

Experimental fault gouges (< 90 μm) align with the most common grain size range of lunar regolith (Colwell et al., 2007) and, given the higher average impact velocities on Mercury (e.g. Marchi et al. 2009), are also representative of Mercury's paleoregolith.

As stated before, Mercury's crust was already fractured by the time that compressional forces began due to the early heavy bombardment, lava sheets cooling, and thermal stresses (Klimczak et al. 2015). This is comparable to the lunar case, where GRAIL mission observations show pervasive fracturing of the crust (Wieczorek et al., 2013). In addition, as on the lunar maria (Fagents et al. 2010), paleoregolith layers are likely interleaving Mercury's lava sheets. This implies that fine-grained, mechanically weakened material was already present in the brittle crust, so the use of gouge in our friction experiments is meant to approximate realistic slip-zone material, not intact deep crustal lithologies. This supports the sounding assumption that Mercury's crustal strength was significantly reduced before major tectonic contraction.

Reviewed Line 99-103: *"We directly tested the mechanical effect of graphite enrichment on mixtures of finely (< 90 μm) crushed basalt and graphite under normal stresses of 5-20 MPa, comparable to ~2 km of overburden on Mercury (Fig. 3a; Methods). Notably, the grain size of the simulated gouges*

overlaps the average size fraction of lunar regolith (i.e., ~70µm [46,47]) and, given the higher impact velocities on Mercury [48], is also a reasonable analogue for Mercurian paleoregolith."

Lines 198-199: This seems like an over-interpretation: the results indicate a weakening agent, but cannot prove that it is graphite.

Please refer to our reply to **LINE 40**.

Line 241-242: Where is the importance of low-angle thrust faults in accommodating Mercury's global contraction demonstrated?

The sentence you refer to is meant as a logical implication of our results, a new global contraction calculation, which is not the aim of our work, would now require a throughout revision of each contractional structure on Mercury surface. If a significant fraction of Mercury's lobate scarps are underlain by shallowly dipping (5–20°), laterally extensive, thrust faults, the same observed surface relief requires more horizontal shortening than in cases of more steeply dipping faults. In other words, if a consistent number of low-angle major thrusts cut through the brittle crust, the total shortening required to build the observed topography would be higher, which would tend to push the global contraction estimate at least toward the upper end of previously published ranges (e.g., Byrne et al., 2014, for reference). This mechanical relationship between fault dip and required horizontal shortening is also discussed in recent geometric forward modeling work (e.g., Loveless and Klimczak, 2025), who show that the assumed thrust-fault dip is one of the dominant controls on the total radial contraction inferred for Mercury.

We note that this is a forward implication of our findings and of published evidences of thrust with low dip angles on Mercury (Galluzzi et al., 2015, 2019; Loveless and Klimczak, 2025), and it relies on the assumption that several prominent lobate scarps can be indeed controlled by low-angle detachments.

For further discussion of this point and of the related modifications made to the manuscript, please refer to our reply to Reviewer 2, comment 2.

Line 255-258: Why would shortening align with the higher estimates of Byrne et al. (2014)? Those were calculated with a (relatively) high dip angle of 25°.

The reviewer is right; the sentence is misleading. As said an estimate of global contraction would now require a throughout revision of each contractional structure on Mercury surface, however it is foreseeable that a good number of prominent lobate scarps insist on low angle detachments. Hence

the estimates of Watters et al. (2021) and at least the more conservative ones of Byrne et al., (2014) cannot hold anymore. Please refer to our reply above (Reviewer 1, **LINE 241-242**)

Reviewed Lines 304-310: *"If a significant fraction of Mercury's lobate scarps are the surface expressions of low-angle thrust faults, the total horizontal shortening -and thus the inferred global contraction- should be substantially higher than previous conservative estimates. In this context, we encourage a future revision of the global catalogue of contractional features to include low-angle thrust faults, as the range of contraction estimates, and thus the planet interpretations of the planet's thermal evolution and internal composition, remain highly sensitive to structural assumptions."*

Line 301: is there a unique relationship between the fault parameters and the basal friction coefficient? If not, how is non-uniqueness dealt with in the MIM?

For a given structure, the relationship between μ_b and the other parameters is not always strictly unique. In the now BFIM we fix H, α_{obs}, and material properties, and then perform a discrete grid search over basal dip (β = 5°, 10°, 15°, 20°) and μ_b (0.05–1.00 in steps of 0.01). All (β, μ_b) pairs that satisfy $|\alpha_{calc} - \alpha_{obs}| \leq 0.1°$ are accepted. This means that in some cases a range of μ_b values is allowed rather than a single best-fit value. We report that range directly in Table 1 / Extended Data Table 1 instead of collapsing it to one number. When no μ_b satisfies the criterion at a given β, we treat that as a geometric constraint (i.e., being β unlikely that low), rather than forcing a friction value. This is how we handle non-uniqueness: by (i) bounding the allowed family of solutions, and (ii) using lack of convergence as information on basal dip. Now, we have clarified this in the revised Methods.

Line 306: Authors mention their MIM distributes uncertainties related to DEM resolution: can this be explained further?

The statement about "distributing uncertainties" refers to how we treat DEM noise when estimating α_{obs}. Rather than inverting each noisy transect separately, we extract multiple transects across each structure, average them to obtain a representative cross section, and then compute α_{obs} from that averaged profile via linear regression (Figure 1d–e). We report the fit statistics (R^2, p-value). BFIM uses that averaged α_{obs} and the corresponding averaged wedge thickness H, so pixel-scale DEM noise is suppressed before inversion. Any remaining geometric uncertainty appears as a range of acceptable μ_b values for each β, instead of as an unstable single number. We have now clarified this in the Methods.

Line 311: a typo in the equation.

This line was corrected in the revised manuscript in the new **Line 356**

Line 313: Is it assumed that ? This seems implied by their definitions.

This line was corrected in the revised manuscript in the new **Lines 356-357**.

Line 316-317: How are the parameter values (cohesion parameters, density, etc.) chosen? How well are these known? How sensitive is to uncertainty in the input values?

The parameter choices are constrained by published estimates for Mercury's crustal properties and by our own experiments; we have clarified this in the revised Methods and within the results in the main text.

We note that, as usual in studies of Mercury's contractional tectonics, some model parameters must be assumed rather than directly measured. This is unavoidable given current data limitations (spatial resolution of available topography, incomplete knowledge of subsurface structure, and the lack of in situ mechanical constraints), and the same type of simplifying assumptions are adopted in previous work interpreting lobate scarps and thrust systems (e.g., Byrne et al., 2014; Watters et al., 2021; Loveless and Klimczak, 2025; Loveless et al., 2025). In our case, we fix cohesion, density, gravity, and wedge friction within plausible bounds (some of which obvious such as gravity and basalt density and others as cohesion and wedge friction even constrained by our own experiments), and then invert for the basal friction needed to reproduce the observed taper. These choices reflect current best estimates rather than uniquely known values. We expect that many of these uncertainties, in particular fault geometry and crustal structure, will be significantly reduced with higher-resolution topography, imaging, and geophysical constraints from the BepiColombo mission.

- **Cohesion** (C for the wedge, S_b for the basal detachment):

We set both cohesion terms to 0.5 MPa. These values are obtained from rotary shear experiments, in which the y-intercept of the normal vs shear stress plot represents the cohesion of the material (Figure 3a). In addition, these values reflect the fact that Mercury's upper crust was already heavily fractured before the onset of large-scale contraction, due to early bombardment, thermal stresses, and cooling of large volcanic sheets (e.g. Schultz, 1993; Klimczak, 2015; Wieczorek et al., 2013). Under those conditions, intact basalt cohesion (which can be several MPa to tens of MPa) is not appropriate. We therefore adopt a reduced cohesion representative of damaged, brecciated crust rather than intact rock. We now state this explicitly in the text. See our previous reply to the comment on **Line 74.**

- **Density (ρ)**:

We use ρ = 3340 kg/m³ as a representative bulk crustal/regolith density for Mercury's basaltic upper crust, consistent with previous estimates of Mercury's surface and near-surface basaltic composition and its high Mg content (e.g. Peplowski et al., 2015; Nittler et al., 2011). We assume this is spatially uniform at the scale of the wedge. Variations of a few percent in ρ do not change the qualitative outcome of the inversion. We added these references and reviewed the information in the Methodology chapter.

- **Gravity (g)**:

We use g = 3.7 m/s², Mercury's surface gravity. This is a well constrained value (e.g., Smith et al., 2012; Genova et al., 2019). We added these references and reviewed the information in the Methodology chapter.

- **Wedge friction (μ_w)**:

μ_w is taken from our basalt gouge rotary shear experiments under dry conditions, as explicitly stated within the manuscript in **Line 110**. In other words, μ_w is measured, not assumed.

- **Basal friction (μ_b)**:

μ_b is the unknown we solve for.

- **Sensitivity**:

in the Basal-Friction Inversion Model we invert μ_b by matching the observed wedge taper (α_{obs}). Because α depends primarily on the ratio between wedge strength and basal strength, moderate changes in cohesion (C, S_b) or density (ρ) can slightly shift the absolute μ_b values but do not change the main result: the required effective μ_b is systematically low.

Remarks on code availability

The code seems to carry out a series of simple calculations based on rows in an excel file. There is a step where several values of mu_b are considered to see which produces a value for alpha matching the observed, but this seems to be a way of numerically solving equations (3)-(5) for the one unknown, mu_b.

We thank the reviewer for looking at the submitted code. The Methodology section has been revised accordingly to reflect this straightforward parameter inversion and to avoid overstating complexity. Please refer to our reply to Reviewer 1, comment 5. We have also updated the capsule description in CodeOcean to reflect the updated name and description of the FBIM.

Reply References (numbered references correspond to those in the manuscript)

K. **Oohashi**, T. Hirose, T. Shimamoto. Shear-induced graphitization of carbonaceous materials during seismic fault motion: Experiments and possible implications for fault mechanics. Journal of Structural Geology 2011 :1122–1134.

K. **Oohashi**, T. Hirose, T. Shimamoto. Graphite as a lubricating agent in fault zones: An insight from low- to high-velocity friction experiments on a mixed graphite–quartz gouge. Journal of Geophysical Research: Solid Earth 2013: 2067–2084.

E. H. **Rutter**, C. J. Peach, D. J. Faulkner. Structure and permeability of graphite-bearing fault rocks, and implications for fault lubrication. Nature Geoscience 2013: 553–558.

Rabinowicz. Friction and Wear of Materials. Wiley 1965 :1–180.

M. L. **Blanpied**, D. A. Lockner, J. D. Byerlee. Frictional slip of granite at hydrothermal conditions. Journal of Geophysical Research: Solid Earth 1995 :13011–13025.

C. H. **Scholz**. The Mechanics of Earthquakes and Faulting. Cambridge University Press 2019

V. **Galluzzi**, G. Di Achille, L. Ferranti, C. Popa, P. Palumbo. Faulted craters as indicators for thrust motions on Mercury. Geological Society, London, Special Publications 2015 :313–325

V. **Galluzzi**, L. Ferranti, M. Massironi, L. Giacomini, L. Guzzetta, P. Palumbo. Structural Analysis of the Victoria Quadrangle Fault Systems on Mercury: Timing, Geometries, Kinematics, and Relationship with the High-Mg Region. Journal of Geophysical Research: Planets 2019 :2543–2562

N. **Tosi**, M. Grott, A.-C. Plesa, D. Breuer. Thermochemical evolution of Mercury's interior. Journal of Geophysical Research: Planets 2013 :2474–2487

M. **Grott**, D. Breuer, A.-C. Plesa. Thermal evolution and global contraction of Mercury. Earth and Planetary Science Letters 2011 :253–268

N. L. **Chabot**, C. M. Ernst, B. W. Denevi, J. K. Harmon, S. L. Murchie, D. T. Blewett, E. D. Zhong, et al. Areas of permanent shadow in Mercury's south polar region ascertained by MESSENGER orbital imaging. Geophysical Research Letters 2012 :L09204

A. **Meunier**. Clays. Springer-Verlag Berlin Heidelberg 2005 :ISBN 3-540-21667-7

N. R. **Izenberg**, R. L. Klima, S. L. Murchie, D. T. Blewett, G. M. Holsclaw, W. E. McClintock, M. D. Dyar. The low-iron, reduced surface of Mercury as seen in spectral reflectance by MESSENGER. Icarus 2014 :364–374.

L. **Kerber**, J. W. Head, D. T. Blewett, S. C. Solomon, L. Wilson, S. L. Murchie, D. L. Domingue. The global distribution of pyroclastic deposits on Mercury: The view from MESSENGER flybys 1–3. Planetary and Space Science 2011 :1895–19

C. J. **Pegg**, J. Wright, M. Massironi, J. K. Steckloff, P. D'Incecco, L. Kerber, D. A. Rothery. Explosive volcanism on Mercury: Geometric and spectral properties of pyroclastic deposits and vents. Icarus 2021 :114510.

L. **Giacomini**, M. Massironi, V. Galluzzi, S. Ferrari, P. Palumbo. Dating long thrust systems on Mercury: New clues on the thermal evolution of the planet. Geoscience Frontiers 2020 :855–870.

L. R. **Nittler,** R. D. Starr, S. Z. Weider, T. J. McCoy, W. V. Boynton, D. S. Ebel, A. L. Sprague, et al. The major-element composition of Mercury's surface from MESSENGER X-ray spectrometry. Science 2011 :1847–1850.

R. J. **Thomas**, D. A. Rothery, S. J. Conway, M. Anand. Hollows on Mercury: Materials and mechanisms involved in their formation. Icarus 2014: 221–235.

P. K. **Byrne,** L. R. Ostrach, C. I. Fassett, C. R. Chapman, B. W. Denevi, A. J. Evans, C. Klimczak, M. E. Banks, J. W. Head, S. C. Solomon. Widespread effusive volcanism on Mercury likely ended by about 3.5 Ga. Geophysical Research Letters 2016: 7408–7416

L. G. **Evans**, P. N. Peplowski, E. A. Rhodes, D. J. Lawrence, T. J. McCoy, L. R. Nittler, J. O. Goldsten, et al. Major-element abundances on the surface of Mercury: Results from the MESSENGER Gamma-Ray Spectrometer. Journal of Geophysical Research: Planets 2012 :E12.
S. Z. **Weider**, L. R. Nittler, R. D. Starr, T. J. McCoy, K. R. Stockstill-Cahill, P. K. Byrne, S. C. Solomon, et al. Chemical heterogeneity on Mercury's surface revealed by the MESSENGER X-Ray Spectrometer. Journal of Geophysical Research: Planets 2012 :E12.
O. **Barraud**, S. Besse, A. Doressoundiram. Low sulfide concentration in Mercury's smooth plains inhibits hollows. Science Advances 2023 :eadd6452.
A. **Lucchetti**, M. Pajola, G. Poggiali, A. Semenzato, G. Munaretto, G. Cremonese, M. Massironi, et al. Volatiles on Mercury: The case of hollows and the pyroclastic vent of Tyagaraja crater. Icarus 2021 :114694.
F. A. **Dahlen**. Critical taper model of fold-and-thrust belts and accretionary wedges. Annual Review of Earth and Planetary Sciences 1990 :55–99
F. A. **Dahlen**, J. Suppe, D. Davis. Mechanics of fold-and-thrust belts and accretionary wedges: Cohesive Coulomb theory. Journal of Geophysical Research: Solid Earth 1984 :10087–10101.
F. **Bilotti**, J. H. Shaw. Deep-water Niger Delta fold and thrust belt modeled as a critical-taper wedge: The influence of elevated basal fluid pressure on structural styles. AAPG Bulletin 2005 :1475–1491.
C.-M. **Yang**, J.-J. Dong, Y.-L. Hsieh, H.-H. Liu, C.-L. Liu. Non-linear critical taper model and determination of accretionary wedge strength. Tectonophysics 2016 :213–226.
U. **Ring**, N. Mortimer, H. Deckert. Critical-wedge theory and the Mesozoic accretionary wedge of New Zealand. Journal of Structural Geology 2019 :1–10.
A. L. **Nahm**, R. A. Schultz. Evaluation of the orogenic belt hypothesis for the formation of the Thaumasia Highlands, Mars. Journal of Geophysical Research: Planets 2010 :115(E4)
H. **Fossen**. Structural Geology. Cambridge University Press, 2016.
Z. T. **Bieniawski**. Engineering rock mass classifications: a complete manual for engineers and geologists in mining, civil, and petroleum engineering. John Wiley & Sons 1989.
R. A. **Schultz**. Brittle strength of basaltic rock masses with applications to Venus. Journal of Geophysical Research: Planets 1993 :98(E6), 10883–10895
R. A. **Schultz**. Limits on strength and deformation properties of jointed basaltic rock masses. Rock Mechanics and Rock Engineering 1995 :1–15.
R. A. **Schultz**. Relative scale and the strength and deformability of rock masses. Journal of Structural Geology 1996 :1139–1149.
M. A. **Wieczorek**, G. A. Neumann, F. Nimmo, W. S. K.iefer, G. J. Taylor, H. J. Melosh, … M. T. Zuber. The crust of the Moon as seen by GRAIL. Science 2013 :671–675.
C. **Klimczak**. Limits on the brittle strength of planetary lithospheres undergoing global contraction. Journal of Geophysical Research: Planets 2015 :2135–2151
W. D. **Carrier** III. Lunar soil grain size distribution. The Moon 6 (1973): 250–263.
S. **Marchi**, S. Mottola, G. Cremonese, M. Massironi, E. Martellato. A new chronology for the Moon and Mercury. Astronomical Journal 137 (2009): 4936–4948.
J. E. **Colwell**, S. Batiste, M. Horányi, S. Robertson, S. Sture. Lunar surface: Dust dynamics and regolith mechanics. Reviews of Geophysics 2007 :2005RG000184P. N.

S. A. **Fagents**, M. E. Rumpf, I. A. Crawford, K. H. Joy. Preservation potential of implanted solar wind volatiles in lunar paleoregolith deposits buried by lava flows. Icarus 2010 :595–604.

T. R. **Watters**. A case for limited global contraction of Mercury. Communications Earth & Environment 2021 :9.

D. E. Smith, M. T. Zuber, R. J. Phillips, S. C. Solomon, G. A. Neumann, et al. Gravity field and internal structure of Mercury from MESSENGER. Science 2012 :214–217.

T. R. **Watters**. A case for limited global contraction of Mercury. Communications Earth & Environment 2021 : 9.

P. K. **Byrne**, C. Klimczak, A. M. Celâl Şengör, S. C. Solomon, T. R. Watters, and S. A. Hauck II. Mercury's global contraction much greater than earlier estimates. Nature Geoscience 7 (2014): 301–307.

S. R. **Loveless**, C. Klimczak. Several kilometers of global contraction on Mercury: A sample-size independent assessment of fault strain. AGU Advances 2025 :e2025AV001715.

S. R. **Loveless**, C. Klimczak, K. T. Crane, and P. K. Byrne. Geometric forward modeling of thrust systems underlying shortening landforms on Mercury. Journal of Structural Geology (2025): 105449.

P. N. **Peplowski**, D. J. Lawrence, L. G. Evans, R. L. Klima, D. T. Blewett, J. O. Goldsten, and S. Z. Weider. Constraints on the abundance of carbon in near-surface materials on Mercury: Results from the MESSENGER Gamma-Ray Spectrometer. Planetary and Space Science 108 (2015): 98–107.

D. E**. Smith**, M. T. Zuber, R. J. Phillips, S. C. Solomon, G. A. Neumann, et al. Gravity field and internal structure of Mercury from MESSENGER. Science 2012, 336, 214–217.

A**. Genova**, S. Goossens, E. Mazarico, F. G. Lemoine, G. A. Neumann, W. Kuang, et al. Geodetic evidence that Mercury has a solid inner core. Geophysical Research Letters 2019 :3625–3633.

REPLY TO REVIEWER 2

Dear reviewer,

We would like to sincerely thank you for your thorough and critical review of our manuscript. Your detailed comments were especially valuable given the complexity of the topic. Your feedback helped us identify key areas that required clarification and refinement, and we feel that the new version substantially improved the original manuscript. We also wish to express our appreciation for your decision to sign your review. In a field where open scientific dialogue is essential, we see this gesture as a sign of transparency and constructive engagement.

Changes in the manuscript that directly address the reviewers' comments are highlighted in bright yellow, while additional modifications to the text are shown using track changes.

Point-by-point reply to Reviewer 2:

I have read the manuscript by Vergara Sassarini et al. titled: "Graphite lubricates Mercury's global contraction". The manuscript analyses 7 shortening landforms interpreted to be fold-and-thrust belts

on Mercury as orogenic wedges using the critical taper approach. Paired with shear experiments using basalt-graphite mixtures, the results indicate that friction coefficients must be much lower than expected for basaltic rock alone, concluding that graphite must be facilitating slip on very low-angle thrust faults. Overall, this is an interesting (and somewhat higher impact) idea that is being tested in this research, and the authors are commended for carrying out this detailed study. I really like the discussion on the confinement in narrow films along the fault plane and the required fault smear, both are concepts that we observe on faults on Earth but have not been considered for faults on Mercury. I have some overall concerns outlined below pertaining to (1) readability of the manuscript and (2) a wide applicability of the model to shortening landforms on Mercury and the interpretations arising from it. In its current format, the manuscript is not ready for publication in Nature Communications and I would personally prefer if the research were communicated in a longer format.

We have now extended the main text to the full allowed length to clarify methods, assumptions, and interpretation, including the critical points raised by the reviewers. A detailed reply to the major and minor concerns of Reviewer 2, are addressed in the text below.

Major Comments Reply

1. The manuscript is very condensed and arrives extremely quickly at the results. Important information that is needed to understand the logic of the research is reported elsewhere. In the presentation of the model and all following text, nearly every sentence refers the reader to another place in the manuscript or has additional information presented in parentheses. Both of these stylistic choices disrupt the flow of the logic and make it hard to read. In addition, Figure 1 is composed of at least 4 stand-alone figures, and therefore the figure caption is not detailed enough. Each of these figures should also be explained in more detail in the main manuscript text, as they are intended to get the point across of how many of the variables are derived. The symbols in the equations in this figure remain unexplained and the equation numbers are not the same as the ones in the Methods part. Together, this makes the description of the mechanical model disjointed. The manuscript would be much improved by separating Figure 1 into multiple figures and tying the methods section into the main text and have all aspects explained in logical order.

We have substantially revised the manuscript to (i) expand the Methods section and, where requested by the reviewers, Results and Discussion, and (ii) improve readability by reorganizing paragraphs and removing unnecessary or repetitive cross references. We now clearly separate Results from

Discussion, keeping observations and model outputs distinct from interpretation. Because Nature Communications places the Methods at the end, we retained brief "Methods" pointers in the main text to direct readers to the full procedural details.

The original Figure 1 has been split into two figures (Fig. 1 and Fig. 2a-d) with expanded, panel-specific captions. In the main text, we now reference and explain how the CT variables are derived. Figure 2 now presents the CT parameters with a notation consistent across the manuscript and explained briefly in the caption, while the full equations are given in the Methods. Together, these changes resolve the disjointedness noted by the reviewer.

2. I have concerns about the overall applicability of the critical taper model. It is a model that was tested on 7 structures and as stated in the manuscript, not even all of them work. There are many other studies investigating the geometry/structural styles of hundreds of faults (Crane and Klimczak, 2019), derive dip angles and faulting depths/ thrust geometries (Petersen et al., 2020; Loveless et al., 2025). From this literature data, hundreds of structures were explained without the need to invoke graphite smear or a critical taper wedge. I have no doubt that the proposed mechanisms are indeed present at Mercury, but it remains unclear if this is widespread. I would regard it as an endmember process that is able to explain a few peculiar structures but isn't necessarily representative of all of Mercury. I agree with the manuscript that all structures on Mercury should be assessed to identify if they require this mechanism and thus have accommodated more strain, but for now (because there is no case that a substantial number of faults have very low dips) the implications for global contraction are not dramatically changed.

Crane, K.T., Klimczak, C., 2019b. Tectonic patterns of shortening landforms in Mercury's northern smooth plains. Icarus 317, 66–80. https://doi.org/10.1016/j.icarus.2018.05.034.

Peterson, G.A., Johnson, C.L., Byrne, P.K., Phillips, R.J., 2020. Fault structure and origin of compressional tectonic features within the smooth plains on Mercury. J. Geophys. Res.: Planets 125. https://doi.org/10.1029/2019je006183.

Loveless, S. R., Klimczak, C., Crane K. T., and Byrne, P. K., 2025, Geometric forward modeling of thrust systems underlying shortening landforms on Mercury. Journal of Structural Geology 198, 105449.

We thank the reviewer for the constructive comments, which can be summarized as follows:

A. Many contractional structures have been already explained and described by the former literature without the need for the Critical Taper mechanism and /or the presence of weakening agents.

B. Critical Taper on low-dip, low-friction detachments cannot be excluded but should be regarded as an endmember that may explain some peculiar structures.

C. There is no evidence of low-angle faulting on Mercury

D. All contractional structures on Mercury should be revised, but no major change in the contraction estimate is expected

For sake of clarity, we address these points in the order C, A, B, D.

C. We respectfully disagree. There are direct evidences for low-angle thrust faulting from DTM-based measurements of faulted craters (Galluzzi et al. 2015, 2019). Unfortunately, several widely cited global contraction estimates were published before these constraints were available (Watters et al. 2004; Di Achille et al. 2012; Byrne et al. 2014) or did not incorporate them (Watters et al. 2021). Assuming Andersonian geometries for all the structures (Watters et al. 2021) or the majority of them (Loveless and Klimczak, 2025), does not reconcile with a substantial fraction of the Galluzzi dataset showing fault dips <20°. Notably, Loveless and Klimczak, (2025) also report shallow dips (~9°-21°, from an average dip of ~9° to a better-fit dip angle of ~21°) for Enterprise Rupes, one of the most prominent shortening landform on Mercury. Taken together, these observations indicate that prominent, hundreds-of-kilometers-long faults on Mercury are not uniformly Andersonian; many are compatible instead with shallow, low-dip geometries and thus with low-friction detachments.

A. The geometric forward models of faulting by Peterson et al. (2020) and Loveless et al. (2025)/ Loveless and Klimczak (2025) (the latter two not yet published at the time of our submission) are based on elastic dislocation codes (e.g., COULOMB) or MOVE. These models fit the observed contractional morphotectonic structures by varying fault geometries and fault depths. This approach is able to model fault geometries, finding multiple solutions that fit the same topography and selecting the best fit. The modelling by those authors results in fault geometries that can fit even the low dip angles observed by Galluzzi et al. (2015, 2019) at the surface, while still satisfying the commonly assumed notion that most of the fault planes should behave in an Andersonian way within the crust invoking flat geometries at different depths. However, changing fault angles at depth has mechanical implications, because fault dip depends on effective friction and/or rheological transitions. Excluding rheological transitions at depths shallower than ~30 km (typical elastic lithosphere thickness, e.g.

Nimmo and Watters 2004; James et al. 2016; Buoninfante et al., 2024), the presence of a weakening agent is unavoidable. If a weakening agent is considered, the assumption of Andersonian faulting does not hold at a global scale anymore. In this context, the Critical Taper model is equally capable of fitting the topography where low dip angles are observed at the surface by inverting μ_b from observed wedge taper and known rock parameters and cross-checked with lab friction experiments. Moreover, it does not require additional assumptions of depth-variable or depth-constrained fault geometries, which in turn can be a source of further non-uniqueness and uncertainties.

B. Building on the discussion above, the Critical Taper model, although an endmember among others, is not only appropriate for the hundreds-of-kilometers-long fold-and-thrust belts considered in our study (e.g., Enterprise Rupes) but is also likely applicable to many systems with similar characteristics. We agree that the model "does not work for all structures", we have clarified this in the manuscript to avoid any misunderstanding on this point. Non-convergence at a dip of 5° simply indicates that the basal detachment for the two considered structures (Enterprise Rupes and the Picasso fault system) must be steeper than 5°. In fact, the model does converge at 10° and 15°, which, for Enterprise Rupes, is consistent with the dip angle estimates from Loveless and Klimczak (2025) (~9-21°, from an average dip of ~9° to a better-fit dip angle of ~21°) and still needs a weakening agent along the fault plane.

D. Existing global contractional estimates already span a wide range :~1-2 km (Watters et al., 2021) up to ~7 km (Byrne et al., 2014) of radius decrease Loveless and Klimczak (2025), favor a range of shortening of ~2-3.5 km considering three published shortening landforms datasets: Watters et al. (2021), Byrne et el. (2014), and Loveless et al. (2024, 2025), and establish that this estimate is independent of the dataset used; nevertheless their calculations are still determined by the combination of thickness, fault dips, and the shapes of the fault plains (Loveless and Klimczak, 2025). In this context, it is clear from points A-B that fault geometries at depth must be critically re-evaluated, as agreed by the reviewer. If shallow-dipping, major thrusts cut through the brittle crust, the total shortening required to produce the observed topography is higher, pushing estimates toward at least the upper end of prior ranges by Byrne et al. 2014. In any case, this issue should be reassessed as new constraints including gravity, topography, and imaging from the BepiColombo mission become available.

Modifications to the manuscript text:

Reviewed Lines 35-39: *"Other recent investigations of the most prominent lobate scarps [14–18] indicate that they can be interpreted as fold-and-thrust belts (FTBs[7]) and suggest the presence of regionally continuous low-angle thrust faults associated with hanging-wall anticlines. Indeed, low-angle thrusts associated with several lobate scarps have been directly derived from deformed craters [15,16] and modeling [19]."*

Reviewed Lines 87-93: "*Constraints on thrust geometry, specifically the basal dip (ß) of the studied thrust faults, come from crater-deformation studies, which treat faulted impact craters as passive strain markers [15,16]. In those cases, vertical rim offset and asymmetric hanging-wall uplift measured in digital terrain models (DTMs) are used to reconstruct the geometry of the shallow portion of the fault, yielding typical dip angles between ~5° and ~15° (Extended Data, Fig. 1). Such shallow dips imply that Mercury's crust is mechanically layered, including weak layers and low-angle faults that operate under effectively low-friction conditions [20,44].* "

Reviewed Lines 155-163: *"The model does not converge for the Picasso fault system and Enterprise Rupes when β = 5° (Fig. 3b; Table 1). This does not represent a model failure but rather a constraint, implying that the basal dip is >5°. Indeed, solutions exist for ß= 10° and 15°. These results can be explained in terms of local structural complications. Enterprise Rupes shows a significant strike-slip component [17], with fault dip angles ranging from ~15° in the frontal thrust to between ~30° and ~57° in the lateral ramps [15]. Our BFIM results for basal dips between 10° and 15° are consistent with both measurements by Galluzzi et al. (2015) and forward modeling of the same structure by Loveless and Klimczak (2025), which yields an average dip of ~9° and a best-fit dip angle of ~21°. A similar reasoning applies to the Picasso fault system, where convergence at ß > 5° likewise indicates a steeper detachment."*

Reviewed Lines 293-304: "*Previous estimates of Mercury's contraction[5,7,79–81] are based on traditional Andersonian fault theory, which predicts that most thrust faults form at moderate to high dip angles (typically 25°-40°) [8], depending on the internal coefficient of friction of rocks, generally assumed in the Byerlee range (i.e., $\mu = 0.6$-0.85)[71]. The different amounts of contraction estimated by various authors mainly depend on the structures assumed to be responsible for global deformation: only major lobate scarp systems (i.e., fold-and-thrust belts) for Watters et al. (2021), who envisaged a radial contraction between ~1.3–2 km, or the majority of lobate scarps and wrinkle ridges distributed across the surface (excluding features within major basins such as Caloris and Rembrandt) for Byrne et al. (2014), who suggested up to 7 km of contraction.*

Regardless of the final estimate, the assumption of Andersonian fault behavior contradicts the low thrust fault dip angles derived by crater deformation analysis [15,16], recent modeling efforts on large FTBs[19], and our CT fits of the observed surface morphology of faults and thrust belts. "

Reviewed Lines 304-310: "*If a significant fraction of Mercury's lobate scarps are the surface expressions of low-angle thrust faults, the total horizontal shortening -and thus the inferred global contraction- should be substantially higher than previous conservative estimates. In this context, we encourage a future revision of the global catalogue of contractional features to include low-angle thrust faults, as the range of contraction estimates, and thus the planet interpretations of the planet's thermal evolution and internal composition, remain highly sensitive to structural assumptions."*

We have also revised the abstract to frame graphite as the most consistent plausible mechanism, rather than a certainty.

Reviewed Lines 384-386: *"We sheared samples composed of basalt and graphite mixtures considered as representative endmembers of Mercury's basaltic crust/paleoregolith and of the most likely weak phase at depth, respectively."*

*The Methods section on friction experiments was further revised, following Reviewer 1's suggestions, to clarify the experimental setup and provide additional technical details (see reply to Reviewer 1, major comment 2)

3. The manuscript has a few places where stress and strain terminology are inconsistently applied, I only list a few, but please check the issue throughout the manuscript. Compression refers to stresses. Normal faulting also requires compression, and so using the term "compression" when referring to structures formed by global contraction is misleading:

- **Line 56**: "Mercury's major compressional architectures" should be "the morphology of shortening landforms on Mercury"
- **Line 92**: "compressional deformation" should be "contractional deformation"
- **Line 94**: "before any major tectonic compression" should be "before the onset of global contraction"

We changed the terminology according to the reviewer suggestion across the text, however it is worth mentioning that there are several examples in literature in which terms as compressional structures or deformation are used as synonyms of contractional structures and deformations (e.g., on planetary surfaces: Watters et al., 1993; Mangold et al., 2000; Mueller et al., 2004; Carboni et al., 2025. On Earth: Ziegler et al., 1998; Li et al., 2021; Frueh et al., 2023). In any case within the manuscript, we modified:

Line 56 to **reviewed Lines 73-75**: *"Our approach highlights the crucial role of graphite-bearing layers in controlling the geometry and mechanics of Mercury's major* ***shortening landforms****, linking surface expressions of crustal deformation to the composition of Mercury's early crust."*

Line 92-94 to **reviewed Lines 108-112**: *"We used $\mu = 0.71$ to constrain the internal friction of the wedge (μ_w) and adopted low cohesive strengths of 0.5 MPa for both the wedge (C) and the detachment (S_b). These values are experimentally supported by the rotary shear results (i.e., cohesion of basalt and basalt-graphite mixtures is < 1MPa; Fig. 3a) and are consistent with previous work indicating that Mercury's crust was already heavily fractured before* ***the onset of global contraction*** *[50–52]."*

4. The extraction of thrust fault dips from faulted craters, its applicability to the interpretation of fault dips, and its application to the critical taper wedge needs a more complete discussion. The cited method detects the local dip at the very shallow portions of thrust faults. Geometric forward modeling using several techniques (not only the COULOMB code, but also MOVE) shows that dips may be variable along the fault. Faulting can be listric, indicating a shallowing of dip at depth. Fault geometries may also show a shallowing of dips at the near surface to accommodate the over-thrusting and folding at the surface. The dip issue becomes apparent when discussing Enterprise Rupes, where the model didn't work so well. The dip discrepancies between the faulted crater method and the geometric forward modeling results at Enterprise Rupes were discussed in Loveless et al., 2025, which supports the findings discussed in line 133.

1. The extraction of thrust fault dips from faulted craters, its applicability to the interpretation of fault dips, and its application to the critical taper wedge needs a more complete discussion.

We agree that this method needs clarification. We address this point in detail in our reply to Reviewer 1, **Line 74**, and we summarize the key aspects here:

Briefly: Galluzzi et al. (2015, 2019) estimate shallow thrust dips on Mercury by treating impact craters cut by thrust faults as passive strain markers. When a thrust fault underthrusts or cuts a crater, it produces a diagnostic pattern of vertical rim offset and horizontal displacement. By measuring these displacements in DTMs and images, fitting a simple thrust geometry to reproduce the observed displacement field, they solve for the local dip of the thrust and slip vector at shallow crustal levels. This method consistently returns dip angles commonly in the 5-20° range for large lobate scarps. Those dip values are the ones we use in our Critical Taper inversion, because the taper formulation requires an input basal dip (β). So β in our model is directly tied to observed shallow dips where available, not assumed.

2. The cited method detects the local dip at the very shallow portions of thrust faults. Geometric forward modeling using several techniques (not only the COULOMB code, but also MOVE) shows that dips may be variable along the fault. Faulting can be listric, indicating a shallowing of dip at depth. Fault geometries may also show a shallowing of dips at the near surface to accommodate the over-thrusting and folding at the surface.

We agree that thrust dips may vary with depth, that some faults may be listric, and that near-surface dip may differ from deeper dip, however the Critical Taper model is equally capable of fitting the topography where low dip angles are observed at the surface and does not require additional assumptions of depth-variable or depth-constrained fault geometries that require changes in friction coefficient or rheology at depth and can be a source of further non-uniqueness and uncertainties. it is also worth mentioning that at the time of the submission of this manuscript, the works from Loveless and Klimczak, (2025) and Loveless et al., (2025) had not yet been published. Now we are pleased that results presented in their work (Loveless and Klimczak, 2025) regarding the dip angle of one of our targets (Enterprise Rupes) are mostly consistent with our own findings.

A few additional clarifications:

Yes, Galluzzi et al. (2015) method constrains the shallow portion of the fault, whereas geometric forward models (e.g., Peterson et al., 2020; Loveless and Klimczak, 2025a; Loveless et al., 2025) explore full-3D geometries at depth using elastic dislocation/MOVE reconstructions. Those forward models allow the dip to vary with depth (including listric geometries), and they can generate multiple

admissible geometries that all reproduce the very same surface relief. In that sense, there is non-uniqueness: more than one subsurface geometry/solution can match the same topography. However, in all cases, the way in which the structure evolves with depth, is still an assumption (at least on Mercury), because all these models ultimately start from the observed topography and then infer the subsurface geometry and mechanics. That inference requires assumptions about depth extent and structural style (e.g., depth of the detachment, whether the fault flattens, pop-up geometries, imbrication, etc.). Those assumptions are necessarily poorly constrained on Mercury because, at this time, we do not have MESSENGER observations (topographic or geophysical) that allow us to determine the fault plane geometry at depth. Importantly, even in those forward models, the "best fit" for some major structures still implies low to moderate dips (e.g., ~20° for Enterprise Rupes; Loveless and Klimczak, 2025), i.e., non-Andersonian behaviour. That is already consistent with the basic premise of our work: these thrust systems do not require ~30-40° everywhere, and in many cases appear mechanically compatible with shallow detachments that would require weak basal friction.

Regarding listric faults and near-surface shallowing, we agree that some thrust systems can have complex dip variations (shallower near the surface, steeper at depth). Our approach does not try to impose or make assumptions on such complexity. Instead, we treat ß as an effective basal dip and solve for the basal friction coefficient needed to sustain the observed taper. In other words, we make a deliberately simple geometric assumption (constant ß at depth), and we invert for μ_b. Forward models, in contrast, typically assume a mechanical behavior (often implicitly assuming higher friction and Andersonian geometries) and then just adjust the geometry until the surface relief is matched. The fact that forward models can match the observed topography without explicitly invoking Critical Taper does not remove the need for a weak detachment. On the contrary, when those models favor shallow effective dips (~10 to 15-20°) over tens to hundreds of kilometers, that geometry at depth shallower than 30 km (i.e. the elastic lithospheric thickness e.g., Nimmo and Watters 2004; James et al. 2016; Buoninfante et al., 2023) is only mechanically viable if the basal interface is weak (low μ_b). That is exactly what we test quantitatively with the now BFIM (ex MIM; see our reply to Reviewer 1, major comment 5).

3. The dip issue becomes apparent when discussing Enterprise Rupes, where the model didn't work so well. The dip discrepancies between the faulted crater method and the geometric forward modeling results at Enterprise Rupes were discussed in Loveless et al., 2025, which supports the findings discussed in line 133.

Enterprise Rupes is a good stress test because it is structurally complicated. It shows a significant strike-slip component (Massironi et al., 2015), and Galluzzi et al. (2015) measured fault dip angles of ~15° in the frontal thrust, increasing to ~30°–57° in the lateral ramps. In our inversion framework, when we force β = 5° the model does not converge, i.e., no μ_b can reproduce the observed wedge taper. This does not mean that "the model fails," but rather that "β = 5° is too low for this structure." When we instead use β = 10°-15°, the model converges and returns reasonable μ_b. This is consistent with Enterprise Rupes having an effective dip steeper than 5°, which is exactly what the Galluzzi measurements and the strike-slip complexity already imply. In addition, Loveless and Klimczak (2025) modelled Enterprise Rupes independently using geometric forward modelling. They report effective dips in the ~9°-21° range (i.e., shallower than Andersonian 30°–35°, but not as low as 5° everywhere). This is entirely consistent with our results: Enterprise Rupes is not a uniformly 5° detachment, but it is also not a classic 30° thrust everywhere. It occupies the same low-to-moderate dip range that, in our model, requires a weak detachment. To clarify this point, we have modified the text within the manuscript as follows:

Reviewed Lines 155-163: *"The model does not converge for the Picasso fault system and Enterprise Rupes when β = 5° (Fig. 3b; Table 1). This does not represent a model failure but rather a constraint, implying that the basal dip is >5°. Indeed, solutions exist for β= 10° and 15°. These results can be explained in terms of local structural complications. Enterprise Rupes shows a significant strike-slip component [17], with fault dip angles ranging from ~15° in the frontal thrust to between ~30° and ~57° in the lateral ramps [15]. Our BFIM results for basal dips between 10° and 15° are consistent with both measurements by Galluzzi et al. (2015) and forward modeling of the same structure by Loveless and Klimczak (2025), which yields an average dip of ~9° and a best-fit dip angle of ~21°. A similar reasoning applies to the Picasso fault system, where convergence at β > 5° likewise indicates a steeper detachment."*

In addition, we agree with Loveless and Klimczak (2025) on the need to reevaluate the global catalog of contractional structures on Mercury in order to better constrain thrust fault dip angles and ultimately obtain more robust estimates of global contraction.

5. As an overall idea, does this research predict orogenic wedges on Mercury to be correlated with regions of increased LRM? Maybe this could be discussed as part of the schematic model (Figure 3).

We actually had a look to the possible correlation between the distribution or regions of increased LRM and the presence of large thrust-faults, but we could not find a straightforward correlation. Due to the long-lasting and intense bombardment and gardening we do not actually expect that the surface LRM distribution strictly reflects the graphite distribution at depth. Even though it is possible that remnants of the primordial graphite-flotation crust may be brought up to the surface as ejecta from large impacts, regions of increased LRM can be explained mainly by the depth of excavation and ejecta distribution of major impact basins (Klima et al., 2018). In general, we think that the low resolution of MESSENGER's data can also hinder the detection of the possible association between LRM and lobate scarps and we nonetheless hope to investigate such association in the future with the higher resolution dataset of Bepi Colombo mission.

Line-by-line Comments Reply

Line 17: "we demonstrate that the observed topography of fold and thrust belts can be only fit by graphite bearing low angle fault slip zones" This is misleading, because the observed topography can only be fit by graphite bearing low angle fault slip zones when using this particular model. Many thrust fault-related landforms can be explained with other thrust geometric models.

While we agree that graphite is a plausible weak phase, we note that models able to fit the topography without Critical Taper typically assume Andersonian dips when building the depth geometry. As you also point out elsewhere, dip angles warrant re-evaluation (see response to comment 2B). If low-to-moderate dips are more common than previously thought, the Critical Taper framework is likely applicable to several systems with similar characteristics. Following your suggestion, we revised Line 17 (now Lines 15-17) to frame graphite as the material that **best explains** our model findings.

Reviewed Line 15-17: "*Using Critical Taper analysis calibrated by friction experiments, we invert basal friction (μ_b) from observed wedge geometry and show that the topography of large fold-and-thrust belts is best explained by graphite-bearing, low-angle fault zones. This finding indirectly supports models proposing a graphite flotation crust during Mercury's early differentiation.*"

Line 18: "This indirectly supports models proposing the formation of a floating layer of graphite during Mercury's early differentiation." This is not true. The method here did not detect graphite on faults, it only invokes graphite. The model fits into the igneous evolution of Mercury.

We agree that our method does not directly detect graphite. Our inference is mechanical: BFIM requires an exceptionally weak phase under dry conditions, and our rotary-shear data show that graphite-rich gouge reproduces the required weakness, whereas hydrated weak phases or long-lived overpressure are unlikely on Mercury. Thus, if slip in a critical taper wedge or in listric systems with flat detachments at depths < 30 km requires graphite, the mechanics indirectly support a substantial graphite content in the crust and the formation of a graphite flotation layer during early differentiation.

Line 57: "crustal deformations and the Mercury's early crust composition" should be "crustal deformation and the composition of Mercury's early crust.

Done.

Reviewed Lines 74-75: *"linking surface expressions of crustal deformation to the composition of Mercury's early crust."*

Line 65: Citation 33 is very general. The chapter should be specified.

We have now specified the book's page for this citation in the References section: "33. Mercury: The View after MESSENGER. Cambridge University Press 2018. pp. 251."

Line 79 and elsewhere: What is the purpose of giving the quadrangle name? It is unclear to anyone unfamiliar with mapping Mercury. It is unexplained in the manuscript. It is fine just mentioning the name of the landforms.

We agree. Quadrangle codes were originally used to shorten the text and are explained in Table 1. We have revised the manuscript to refer only to the landform names in the main text, as suggested. Quadrangle labels are preserved in Table 1 (and figure captions) for cross-reference.

Line 149: "Our analysis on Mercury fold and thrust belts shows that remnants of this layer must persist beneath the present upper crust" This is misleading. The analysis only shows that the utilized model requires a weakening lithology, and graphite is a plausible choice. As stated above, the model may not be universally applicable to all such landforms on Mercury. If a reader doesn't consider this model applicable (of which I am sure there are some), then a remnant of this layer does not necessarily need to facilitate faulting.

We agree that the original phrasing could be interpreted too strongly. Our results do not demonstrate that a graphite-rich layer universally persists beneath the entire upper crust of Mercury. What we can say is more limited: for the specific large fold-and-thrust belts we analyzed, the basal detachments require very low effective friction, which is consistent with the presence of a mechanically weak

phase. Graphite is the most plausible candidate for that phase for reasons now explained within the main text and in our answer to the question of Reviewer 1 about line 40. In addition, (i) graphite is expected from Mercury's early flotation crust and has been proposed to survive in the shallow subsurface in localized pockets (e.g., Denevi et al., 2009; Kaaden and McCubbin, 2015; Klima et al., 2018), and (ii) our friction experiments show that graphite-rich gouges can reproduce the low μ_b values required by the Critical Taper inversion. We recognize that other structures on Mercury may be accommodated by different fault geometries that do not require such a weak basal detachment. For this reason, in the revised manuscript we have softened the wording to indicate that graphite-rich material is the most plausible weakening agent beneath some thrust systems, rather than implying that it must be present everywhere.

Reviewed Lines 197-199: "*Notably, our analysis on Mercury's fold-and-thrust belts suggests that remnants of this graphite-rich layer may locally persist beneath parts of the present upper crust.*"

Line 207 and Figure 3b: partial melting and volcanic resurfacing is not very obvious in the image.
We have modified Figure 4, panel **b** to emphasize the resurfacing. Please refer to the revised Figure 4 in the manuscript. In addition, we added texture to the crust shown in panels **b** and **c**, to highlight the fractured crust.

Line 222: either remove "subsurface" or "at depth" as one of them is enough.
We have removed "at depth" in **Line 270**, in the revised manuscript.

Figure 4 and associated discussion: the friction coefficient of 0.1 showing "this study" is misleading. This study really shows that pure graphite has a friction coefficient of 0.11 and the model solutions yielded a coefficient of 0.3 for ~25% graphite, as stated in line 220.
Agree, we have corrected Figure 4 (now, Figure 5) adding the average of the derived friction coefficient.

In addition, the statement in line 264: "Lower fault strength and shallower thrust faults lead to more efficient crustal shortening compared with stronger and steeper faults which result in less shortening" is incorrect (or the logic is reversed). The fault doesn't cause shortening from global contraction. Shortening from global contraction causes the fault. It's just that shallower thrust fault dips require more shortening to produce the observed topography when compared to the same topography produced by more steeply dipping thrust fault. Imagine Mercury undergoing a certain finite amount

of global contraction, the same amount can be accommodated either by shallowly or steeply dipping thrusts. So instead of "lead to more efficient crustal shortening" I would put "require more shortening compared to more steeply dipping thrusts" and leave it at that.

Thank you for the insight, we agree that the sentence could be unclear as written. We have revised the manuscript following your suggestion:

Reviewed Lines 313-314: "*Lower fault strength and shallower thrust faults require more shortening compared to more steeply dipping thrusts.*"

Line 339: A noun is missing after "sheared". Maybe "samples"?

We added the missing word "samples" to the manuscript (**Line 384**)

Remarks on code availability

I have clicked through the code and visually inspected the structure, but I did not try to run it from my computer.

We have updated the capsule description in CodeOcean to reflect the updated name and description of the FBIM.

Reply References (numbered references correspond to those in the manuscript)

V. **Galluzzi**, G. Di Achille, L. Ferranti, C. Popa, P. Palumbo. Faulted craters as indicators for thrust motions on Mercury. Geological Society, London, Special Publications 2015 :313–325

V. **Galluzzi**, L. Ferranti, M. Massironi, L. Giacomini, L. Guzzetta, P. Palumbo. Structural Analysis of the Victoria Quadrangle Fault Systems on Mercury: Timing, Geometries, Kinematics, and Relationship with the High-Mg Region. Journal of Geophysical Research: Planets 2019 :2543–2562

T. R. **Watters**, M. S. Robinson, C. R. Bina, P. D. Spudis. Thrust faults and the global contraction of Mercury. Geophysical Research Letters 2004 : L04001.

G. **Di Achille**, C. Popa, M. Massironi, E. M. Epifani, M. Zusi, G. Cremonese, P. Palumbo. Mercury's radius change estimates revisited using MESSENGER data. Icarus 2012 : 456–460.

T. R. **Watters**. A case for limited global contraction of Mercury. Communications Earth & Environment 2021 : 9.

S. R. **Loveless**, C. Klimczak. Several kilometers of global contraction on Mercury: A sample-size independent assessment of fault strain. AGU Advances 2025 :e2025AV001715.

S. R. **Loveless**, C. Klimczak, K. T. Crane, and P. K. Byrne. Geometric forward modeling of thrust systems underlying shortening landforms on Mercury. Journal of Structural Geology (2025): 105449.

G. A. **Peterson**, C. L. Johnson, P. K. Byrne, R. J. Phillips. Fault structure and origin of compressional tectonic features within the smooth plains on Mercury. Journal of Geophysical Research: Planets 2020 : e2019JE006183.

F. **Nimmo**, T. R. Watters. Depth of faulting on Mercury: Implications for heat flux and crustal and effective elastic thickness. Geophysical Research Letters 2004 : 31(2).

P. B. **James**, R. J. Phillips, M. Grott, S. A. Hauck II, S. C. Solomon. The thickness of Mercury's lithosphere inferred from MESSENGER gravity and topography. Journal of Geophysical Research: Planets 2016

S. **Buoninfante**, M. Milano, B. Negri, C. Plainaki, G. Sindoni, M. Fedi. Gravity evidence for a heterogeneous crust of Mercury. Scientific Reports 2023, 13, 19854.

M. **Massironi**, G. Di Achille, D. A. Rothery, V. Galluzzi, L. Giacomini, S. Ferrari, and P. Palumbo. Lateral ramps and strike-slip kinematics on Mercury. Icarus 2015, 248, 385–397.

P. K. **Byrne**, C. Klimczak, A. M. Celâl Şengör, S. C. Solomon, T. R. Watters, and S. A. Hauck II. Mercury's global contraction much greater than earlier estimates. Nature Geoscience 7 (2014): 301–307.

S. R. **Loveless**, C. Klimczak, L. R. McCullough, K. T. Crane, S. M. Holland, P. K. Byrne. A statistical evaluation of the morphological variability of shortening landforms on Mercury. Icarus 2024, 416, 116106.

N. **Mangold**, P. Allemand, P. G. Thomas, and G. Vidal. Chronology of compressional deformation on Mars: Evidence for a single and global origin. Planetary and Space Science 2000, 48(12–14), 1201–1211.

K. **Mueller** and M. Golombek. Compressional structures on Mars. Annual Review of Earth and Planetary Sciences 2004, 32(1), 435–464.

F. **Carboni**, O. Karagoz, J. B. Ruh, and T. Kenkmann. Gravity-driven fold–thrust belt at the Martian dichotomy. Earth and Planetary Science Letters 2025, 671, 119645.

P. A. **Ziegler,** J. D. van Wees, and S. Cloetingh. Mechanical controls on collision-related compressional intraplate deformation. Tectonophysics 1998, 300, 103–129

W. **Li**, F. Zhang, Y. Zhang, L. Fu, and H. Li. Characteristics and formation mechanisms of Mesozoic compressional structures in the Huanghua Depression, Bohai Bay Basin, China. Australian Journal of Earth Sciences 2021, 68, 746–761.

T. **Frueh**, H. Hiesinger, C. H. van der Bogert, J. D. Clark, T. R. Watters, and N. Schmedemann. Timing and origin of compressional tectonism in Mare Tranquillitatis. Journal of Geophysical Research: Planets 2023, 128, e2022JE007533.

R. L. **Klima**, B. W. Denevi, C. M. Ernst, S. L. Murchie, and P. N. Peplowski. Global distribution and spectral properties of low-reflectance material on Mercury. Geophysical Research Letters 2018, 45, 2945–2953.

B. W. **Denevi**, M. S. Robinson, S. C. Solomon, S. L. Murchie, D. T. Blewett, D. L. Domingue, N. L. Chabot, et al. The evolution of Mercury's crust: A global perspective from MESSENGER. Science 2009, 324, 613–618.

K. E. Vander **Kaaden**, F. M. McCubbin. Exotic crust formation on Mercury: Consequences of a shallow, FeO-poor mantle. Journal of Geophysical Research: Planets 2015, 120, 195–209.

Manuscript NCOMMS-25-45873B

We want to thank the reviewers for their enormous contributions to improving and refining this work, which has now acquired a more robust shape. In particular, we thank Reviewer #1 for the detailed and thorough review that allowed us to correct the typo in our equations. Although corrections to the reported values were necessary, the main scientific finding of this work remains unvaried. ***Modifications to the previously submitted manuscript are shown in the highlighted version included within the submitted files.***

REPLY TO REVIEWER 1

Point-by-point reply to Reviewer 1:

The manuscript organization and presentation have been very much improved. Overall, the manuscript is clear, well-illustrated, and quite readable. I maintain my opinion that the experiments are a useful contribution to the community, as is a quantification of the physical properties implied by particular fault dips. Furthermore, I appreciate the detailed responses to my previous comments, which have been largely addressed.
However, in reading the new manuscript, I have one major new concern regarding the calculation of basal friction; I believe the values calculated by the code do not match the equations presented in the manuscript, and that recalculation and possibly reinterpretation of the results and conclusions may be necessary.
I have provided a more detailed version of this comment as well as several more less major comments in the uploaded file.

Major Comment Reply (from the attached pdf file)

While attempting to verify whether the suggested direct solution of equations 6-8 (see later comment) reproduces the authors' predicted values of μb, I found that the equations implemented in the provided code do not match those presented in the manuscript.
Specifically, the code implies:

$$\mu_b = (1+W)\frac{\alpha}{\beta} + W - \frac{S_b}{\rho g H} \quad (1)$$

with

$$W = 2\sin(\phi)(1-\sin(\phi)) + \frac{C}{\rho g H} \quad (2)$$

Using these equations, I am able to reproduce the values for μb reported in Extended Data Table 1, using $\alpha calc$ for α to ensure comparability with the authors' calculations.
However, the equations as written in the manuscript lead to:

$$\mu_b = (1+W)\alpha - W\beta - \frac{S_b}{\rho g H} \tag{3}$$

with

$$W = 2\frac{\sin(\phi)}{1-\sin(\phi)} + \frac{C}{\rho g H} \tag{4}$$

I verified that the equations written in the manuscript (from which equations 3-4 are derived algebraically) are consistent with those in Suppe, 2007. I have not performed an exhaustive recalculation, but a few examples demonstrate that basal friction coefficients calculated using these equations can sometimes differ substantially from the presented results:

Profile	β	α	μ_b from Equations 1-2	μ_b from Equations 3-4
P1_ER	5	0.82	0.24	0.28
P1_ER	10	0.79	0.37	0.53
P1_ER	15	0.78	0.41	0.77
P1_AH	5	1.45	0.05	0.32
P1_PC	10	2.58	0.1	0.64
P5_EntR	10	2.91	0.05	0.66
P5_PR	15	0.68	0.42	0.76

It is therefore necessary to ensure that the code is solving the equations presented in the manuscript. Resolving this inconsistency is likely to lead to updated values for μb, which may in turn affect the interpretation of the results and the conclusions of the manuscript.
I note that it is to the authors' credit that their effort to make their analyses transparent and reproducible and the clarity of their methodological presentation enabled this assessment.

We thank Reviewer #1 for the thorough and careful reading of the manuscript, and particularly for taking the time to independently verify the calculations using the provided code and supplemental data. Upon checking, we confirmed that there was indeed a typo in the code: a multiplication operator was used instead of a division operator in the formula for W (wedge strength), as identified by the reviewer. This error led to biased estimates of μ_b. We have corrected the code (updated in the Ocean Code capsule linked to this submission) and, following the reviewer's suggestion, replaced the inversion approach with a direct calculation. The revised algorithm yields updated μ_b values for all analyzed targets, which still support the main interpretations and conclusions of the manuscript. In addition, revisiting the calculations provided an opportunity to more carefully cross-check all input parameters, resulting in a more robust and well-constrained analysis overall. We believe the updated results offer a clearer and more accurate picture of the role of graphite in facilitating low-angle fault formation and slip on Mercury.

The main changes are in the "***Critical Taper modeling of weak thrust faults***" section of the revised manuscript. Tables and figures containing μ_b results were updated accordingly. Tables and figures form the "Extended Data" section as well as the material within "Data Source" containing μ_b results, were also updated. Point-by-point additional comments, along with insights on the updated results, are addressed below.

Additional Comments Reply

Line 29-30: I suggest replacing "primarily" with ", for example,". There is not a consensus that planetary contraction is driven primarily by secular cooling. For example, substantial contraction could have been driven by inner core crystallization.

We respectfully chose to maintain the use of "primarily," as there is broad consensus in the literature that secular cooling is the main driver of global contraction on Mercury (e.g., Watters et al., 2004; Byrne et al., 2014; Giacomini et al., 2020; Watters et al., 2021; Loveless and Klimczak, 2025; Klimczak et al., 2015). Inner core crystallization is itself a consequence of secular cooling rather than an independent process (e.g., Solomon, 1976, 1977; Schubert et al., 1988; Hauck et al., 2004; Grott et al., 2011) and therefore does not represent a separate competing mechanism.

Line 31: Byrne et al. estimate something like 4-7 km. Why give a range for the low estimates but not for the high?

Revised Lines 31-32: "*The estimated decrease of planetary radius widely ranges from 1-3 km* [5,6] *to ~4-7 km* [7] *(Fig. 1)*"

Line 33: Would be clearer if "Andersonian fault geometry" were either defined or a paper which gives a definition cited.

We added the citation after Anderson's theory from Anderson (1905) and Sibson (2012):

Revised Lines 34-36: "*and assume "Andersonian" fault geometries* **(Andreson, 1905; Sibson, 2012)** *to constrain fault parameters such as dip angle (i.e., ~30-35°), depth of faulting, and displacement of large-scale lobate scarps*"

And we added a better-defined explanation in the paragraph just below:

Revised Lines 40-43: "*…the formation of low-angle faults is inconsistent with Mohr-Coulomb rheology, in which faults nucleate at 30-40° to the major principal stress σ1 (that is horizontal in contractional regimes in the context of Anderson's theory* [8,9]*).*"

Figure 2: much improved, very informative.
We thank the reviewer for the positive feedback!

Lines 137-143: The way the model is presented is still confusing. The BFIM is a numerical function solver. It should be possible to solve for the μb which gives the observed topographic angle with arbitrary accuracy. I don't really understand why there is a distinction between αobs and $\alpha calc$ at all, or why the error quantification is necessary. From looking at the code, perhaps there needs to be a separate discussion of fitting αobs?
As mentioned above and after your suggestions, we replaced the inversion approach with a direct calculation method, as shown in the revised Ocean Code capsule linked to this submission. We have updated the "Mechanical Model" methods section to clarify that μ_b is now computed analytically. In addition, the calculation pipeline has been renamed from Basal-Friction Inversion Model to Basal-Friction Model (BFM) to better reflect the updated direct calculation approach.

Line 155: Is there another way to express the lack of solution other than "The model does not converge": convergence has a specific meaning which I'm not sure is relevant here. "No solution is possible"? "A value for μb outside the range considered would be necessary"?
Since we have adopted an analytical approach and updated the calculations, we have revised the text accordingly. In the direct calculations presented in the revised manuscript, there are no convergence issues. Please, refer to the "**Critical taper modeling of weak thrust faults**" section for more details.

Lines 156-157: This is a nice sentence which adds clarity, though my previous comment remains.
See the reply on the previous comment.

Line 189-190: I don't believe the onset of explosive volcanism is well constrained, since early explosive deposits could easily have been overprinted. Furthermore, given the planet-wide resurfacing which seems to have occurred/ended at 4.1-4.0 Gya (Marchi et al., 2013), an onset time of 3.9 Gya cannot be very firm. I believe the author's argument (that elevated pore pressure due to volcanic degassing is likely not the weakening agent) stands without any timing constraint.
Following the reviewer's suggestion, we have removed the sentence related to the timing of explosive volcanism. The sentence now reads as:

Revised Lines 197-201: "*Even if explosive volcanism and volatile-driven degassing have been documented on Mercury by the presence of pyroclastic vents* [66–68] *and faculae* [69,70]*, this activity is both spatially limited and transient, and is therefore unlikely to have maintained elevated pore pressures across the hundreds-to-thousands of kilometers scales and ~100 Myr timescales characteristic of large FTBs* [4,55]. "

Line 215-219: There are a few specifics here which I do not think are well supported, and removing them may strengthen the authors' points or widen the applicability of their findings. First, is it necessary for the deep graphite-rich layers to be primordial? The primordial graphite layer (the original flotation crust) ought to be buried beneath the 30 or so km of crust inferred from gravity data. But couldn't graphite just as well be sourced from shallower layers emplaced by impacts (that is, the deep graphite brought up in ejecta or melt pool differentiation)? I suggest simply removing the word "primordial".

We thank the reviewer for these insights. Regarding the first point, following the reviewer's suggestion we have removed the word 'primordial' (**see reviewed lines 219-222**), since we agree that graphite lubricating detachments do not require a direct connection to an intact, deep flotation crust/primordial layer; graphite could be supplied by impact-emplaced shallower pockets and layers. This is supported across the manuscript, for example, referring to the work of Klima et al. (2018), and is reflected in the following lines:

Lines 261-263: "*Continuous impacts to the planet fractured and reshuffled the primary and secondary crust, resulting in a dense fracture network (mega-regolith like) and graphite-rich pockets and layers distributed at different crustal depths*"

Lines 58-62: "*This material is thought to be derived from a graphite-rich flotation crust formed during the early stages of crustal differentiation and subsequently excavated by large impacts* [28,33–35]. *Remnants of this layer, heterogeneously distributed and disrupted by early bombardment, may persist at depth beneath the present-day upper crust and, through impact gardening, be transported into shallower layers or locally exposed at the surface* [29,33–35]."

Second, I am still unconvinced that graphite is the *most* plausible weakening agent, but rather that graphite is *a* plausible weakening agent. There are other possible weakening agents (e.g. sulfides) which have not been evaluated – not that they need to be mentioned, but "most" implies they have been evaluated. But the study also establishes how weakening an agent must be, which is a powerful contribution whether or not the agent turns out to be graphite – why limit it? The authors make a strong point that the weakening agents/processes typical for Earth should not be present on Mercury. This inspires the search for Mercury-typical weakening agents such as graphite.

Regarding the second point, we agree with the reviewer's wording concern. In the revised manuscript we avoid phrasing that implies we have exhaustively evaluated all potential weakening agents. We therefore replace "most plausible" with "a plausible candidate" where appropriate. Regarding sulfides, we acknowledge they can be present on Mercury and may be involved in localized volatile related processes (e.g., hollows and pyroclastic deposits). However, at present we are not aware of evidence that sulfide rich gouges act as a widespread, long lived low friction detachment analogue at the regional scales and timescales required for the major fold and thrust belts studied here. As well, sulfides are not documented to act as primary weakening agents in Earth fault gouges to our

knowledge. Consistent with MESSENGER geochemical observations indicating sulfur bearing phases (e.g., Nittler et al., 2011; Weider et al., 2012; Evans et al., 2012), we note that such occurrences are commonly linked to localized surface expressions (e.g., Barraud et al., 2023; Lucchetti et al., 2021) and therefore are unlikely to explain basin scale detachments. For these reasons, we do not emphasize sulfides as the primary explanation, while we present graphite as one plausible weakening phase consistent with both Mercury's geochemical context and the experimentally required friction reduction.

Reviewed Lines 222-225: "*In this context, our modeling and experiments indicate that graphite-rich material along low-angle anisotropies is a plausible weakening agent for the low friction coefficients inferred along Mercury's thrust detachments…*"

Figure 3: this figure is difficult to read because of its low resolution. I'm confused about the internal coefficient of friction. Is *μw* that of pure basalt? It looks like the figure is communicating that 25% graphite is assumed in the wedge, but the main text implies that graphite is present just in the shear zone and therefore lowering only the basal coefficient of friction.

We have updated Figure 3 to improve resolution and to incorporate the revised μ_b values from the corrected CT equations. Regarding μ_w: across the manuscript (see reviewed lines below) we describe Mercury's crust as a damaged basaltic material partially mixed with minor amounts of graphite, consistent with surface observations of LRM (e.g., Klima et al., 2018) and the expected effects of early bombardment (e.g., Ernst et al., 2010) we mention this both in the previous version and in the reviewed manuscript). A μ_w constrained by pure basalt gouge (μ_w = 0.71) is therefore physically inconsistent with this crust model. In the revised manuscript, we adopt μ_w = 0.59, corresponding to the experimental friction of a basalt-dominated gouge with a minor graphite quantity (~5 vol%; Fig. 3a), which may better reflect the assumed crustal composition and strength.

Reviewed Lines 116-119: "*Considering a basaltic crust partially mixed with minor amounts of graphite (e.g., 5%; Fig.3a) consistent with the relative weak crust needed for the concurrent action of global contraction and tidal despinning [53], and with the estimated surface content of LRM [28,29,51]*"

Reviewed Lines 210-213: "*This implies that deformation in Mercury's thrust fault systems localized within pre-fractured, megaregolith-like material mixed with a minor amount of graphite rather than intact basalt, suggesting that the crust strength was reduced before any major tectonic compression*"

Reviewed Lines 213-216: "*Within this framework, the frictional behavior of the simulated crust composition of our experiments (Fig. 3a; μ_w = 0.59) represents that of a damaged basaltic crust interleaved with paleoregolith layers, transected by inherited anisotropies underlined by fine gouges and intermixed with a slight admissible content of graphite*"

Reviewed Lines 266-267: "*…the graphite flotation crust must have been significantly mixed into the early-forming secondary crust, and graphite abundances are likely higher at depth*."

The 25 vol.% graphite value in the previous manuscript did not represent the bulk graphite content of the wedge, but rather the experimental fault gouge composition required to match the lowest μ_b values inferred from the BFM. With the updated and more realistic μ_w and corrected BFM equations, the inferred μ_b range at the basal detachment shifts, and the corresponding experimental graphite contents required to reproduce it span a broader and more geologically reasonable range of ~5-40 vol.%. As described in the revised manuscript, this enrichment need not to be present throughout the bulk crust since graphite can be confined to the principal slip zone and mechanically concentrated along the detachment by weak phase smearing, making even modest bulk abundances sufficient to sustain low basal friction.

Line 257: LRM is defined in two places in two ways (material vs. materials). I have most commonly seen material (not plural), but regardless, one should be chosen.
We now use Low Reflectance Material (LRM) consistently across the manuscript.

Figure 4: This is a really nice illustration: informative and beautiful.
We thank the reviewer for the kind comment. Considerable effort was devoted to making the figure both informative and visually clear.

Line 269-276: This is a very important and well-stated point. Given the inference of ~1 wt.% carbon in Mercury's crust in general, is a greater concentration of graphite at depth (i.e., LRM) even necessary to explain the fault weakening?
We agree that a high bulk graphite concentration at depth is not necessarily required. Although our friction experiments indicate that reproducing the inferred weakness corresponds to mixtures on the order of 5-10 up to ~40 vol.% graphite, this value should be interpreted as the local composition of the principal slip zone, not as the average composition of the crust or of a regionally extensive layer. In our interpretation, weakening can be achieved if graphite is mechanically localized into very thin films (mm to sub-mm) along the detachment and maintained by smearing, so that a small total mass/volume fraction of graphite at the planetary scale can have a disproportionately large effect on fault strength. Therefore, the inferred ~1 wt.% and locally enriched up to 5% in the most concentrated low-reflectance deposits (e.g., dark spots; Wang et al., 2022) in Mercury's crust is not inconsistent

with the required fault weakness, because only a small amount of carbon needs to be concentrated within slip surfaces. Local graphite-rich pockets or layers at depth could act as the source material for this localization.

Line 290-291: I am a bit confused by this statement. As I understand it, this manuscript does not really go into how low-angle thrust faults accommodate global contraction, but rather gives a plausible weakening mechanism that would allow for the low angles previously observed for thrust faults.
We thank the reviewer for this important clarification. We agree that the aim of this study is not to directly model how low-angle thrust faults accommodate global contraction. Rather, our objective is to propose and test a plausible weakening mechanism that could enable the formation and slip of low-angle thrust faults, as previously inferred from observations. We have revised the abstract to better reflect the scope of the work and avoid potential overinterpretation of our results:

Reviewed Lines 19-22: "*We infer that remnants of this carbon-rich layer, heterogeneously distributed within Mercury's crust because of early impacts, caused the weakening of large portions of the crust during later contraction, allowing the nucleation of weak, low-angle thrust faults*."

Line 293-296: The authors have presented calculations of low friction coefficients assuming a low dip angle. Does the model predict high friction coefficients at this classic high dip angle?
Yes. For fixed wedge properties and geometry, the Critical Taper relations predict that the basal friction required to reproduce a given taper increases with increasing basal dip. Accordingly, when we explore higher dip angles in BFM, the inferred μ_b shifts to higher values. This trend is shown explicitly in the reviewed Fig. 3b, where μ_b increases linearly (up to 15°) with ß.

Line 313: Planetary should not be capitalized.
This line has been corrected in the new revised manuscript.

Line 340: How is the starting point of the transect determined? This seems extremely important for calculating the bulge area and the height. Is there some fitting that needs to be described here?
The starting point of each transect was fixed at a distance ranging from 20 to 30km from the fault intersection point and applied consistently across all targets to ensure comparability, paying attention to avoid eventual major topographic variations (e.g., major impact craters or basins) in the thrust system proximity. The transects also captures a portion of footwall to better visualize the topography. The topographic slope (α) was then determined by linear regression over the full transect length, as described in the Methods section.

Line 352: This section is contributing to my confusion about whether μw must be lowered or only μb.
Please refer to the reply to the comment about "Figure 3" and to the reviewed "**Mechanical Model**" section.

Line 361-364: It should be possible to achieve $\alpha calc = \alpha obs$ with arbitrarily good precision by decreasing $\Delta\mu b$. I find it quite confusing for this to be presented as a fit-and-error calculation when it really seems to be simply a numerical solution to a function. In fact, an inversion is not necessary; Equations 6-8 permit an analytical solution for μb as a function of alpha as in Equation (3) above, repeated here:

$$\mu_b = (1 + W)\alpha_{obs} - W\beta - \frac{S_b}{\rho g H}$$

Following the reviewer's suggestion, we have replaced the iterative inversion with a direct analytical solution for μ_b, as shown in the revised "**Mechanical Model**" section and the updated Ocean Code capsule linked to this submission.

Extended Data Table 1: The columns need units.
The units were added to the reviewed Extended Data, Table 1.

Remarks on code availability

I have visually inspected the code, focusing particularly on the part calculating the basal friction coefficient. I have skimmed but not read in detail the portions which fit the transect parameters and read input files. I have not run the authors' code.

References

***numerical references are to be find within the manuscript**

Anderson, E. M. The dynamics of faulting. Trans. Edinb. Geol. Soc. 8, 387–402 (1905).

Barraud, O., Besse, S. & Doressoundiram, A. Low sulfide concentration in Mercury's smooth plains inhibits hollows. Sci. Adv. 9, eadd6452 (2023).

Byrne, P. K. et al. Mercury's global contraction much greater than earlier estimates. Nat. Geosci. 7, 301–307 (2014).

Ernst, C. M. et al. Exposure of spectrally distinct material by impact craters on Mercury: Implications for global stratigraphy. Icarus 209, 210–223 (2010).

Evans, L. G., Peplowski, P. N., Rhodes, E. A., Lawrence, D. J., McCoy, T. J., Nittler, L. R., & Goldsten, J. O. (2012). Major-element abundances on the surface of Mercury: Results from the MESSENGER Gamma-Ray Spectrometer. Journal of Geophysical Research: Planets, 117(E12).

Giacomini, L., Massironi, M., Galluzzi, V., Ferrari, S. & Palumbo, P. Dating long thrust systems on Mercury: New clues on the thermal evolution of the planet. Geosci. Front. 11, 855–870 (2020).

Grott, M., Breuer, D. & Laneuville, M. Thermo-chemical evolution and global contraction of mercury. Earth Planet. Sci. Lett. 307, 135–146 (2011).

Hauck II, S. A., Dombard, A. J., Phillips, R. J., & Solomon, S. C. (2004). Internal and tectonic evolution of Mercury. Earth and Planetary Science Letters, 222(3-4), 713-728.

Klima, R. L., Denevi, B. W., Ernst, C. M., Murchie, S. L. & Peplowski, P. N. Global Distribution and Spectral Properties of Low-Reflectance Material on Mercury. Geophys. Res. Lett. 45, 2945–2953 (2018).

Klimczak, C. Limits on the brittle strength of planetary lithospheres undergoing global contraction. J. Geophys. Res. Planets 120, 2135–2151 (2015).

Loveless, S. R. & Klimczak, C. Several Kilometers of Global Contraction on Mercury: A Sample-Size Independent Assessment of Fault Strain. AGU Adv. 6, e2025AV001715 (2025).

Lucchetti, A. et al. Volatiles on Mercury: The case of hollows and the pyroclastic vent of Tyagaraja crater. Icarus 370, 114694 (2021).
Nittler, L. R. et al. The Major-Element Composition of Mercury's Surface from MESSENGER X-ray Spectrometry. Science 333, 1847–1850 (2011).
Schubert, G., Ross, M. N., Stevenson, D. J., & Spohn, T. (1988). Mercury's thermal history and the generation of its magnetic field. Mercury, 1988, 429-460.
Sibson, R. H. Reverse fault rupturing: competition between non-optimal and optimal fault orientations. Geol. Soc. Lond. Spec. Publ. 367, 39–50 (2012).
Solomon, S. C. (1976). Some aspects of core formation in Mercury. Icarus, 28(4), 509-521.
Solomon, S. C. (1977). The relationship between crustal tectonics and internal evolution in the Moon and Mercury. Physics of the Earth and Planetary Interiors, 15(2-3), 135-145.
Wang, Y., Xiao, Z., Xu, R., Xiao, Z., & Cui, J. (2022). Dark spots on Mercury show no signs of weathering during 30 Earth months. Communications Earth & Environment, 3(1), 299.
Watters, T. R., Robinson, M. S., Bina, C. R. & Spudis, P. D. Thrust faults and the global contraction of Mercury. Geophys. Res. Lett. 31, 2003GL019171 (2004).
Watters, T. R. A case for limited global contraction of Mercury. Commun. Earth Environ. 2, 9 (2021).
Weider, S. Z., Nittler, L. R., Starr, R. D., McCoy, T. J., Stockstill-Cahill, K. R., Byrne, P. K., & Solomon, S. C. (2012). Chemical heterogeneity on Mercury's surface revealed by the MESSENGER X-Ray Spectrometer. Journal of Geophysical Research: Planets, 117(E12).

REPLY TO REVIEWER 2

We would like to thank the reviewer for their comments on this second version of the manuscript. We agree that their previous comments and corrections, as well as these minor requests, have greatly improved the quality of the manuscript.

Point-by-point reply to Reviewer 2:

I have read the revised version of the manuscript by Vergara Sassarini et al. titled: "Graphite lubricates Mercury's global contraction". The manuscript is much improved and reads well at this time. My previous comments were either incorporated into the revised manuscript and/or they were discussed in detail in the response letter. I have a few minor remarks for this version, but I think the manuscript is otherwise ready for publication.

Lines 40 and 46: I would reword the term "severely misoriented" when referring to low angle thrust faults. I would describe it as "inconsistent with Mohr-Coulomb theory in the context of Andersonian faulting". It is probably not accurate to say "non-Andersonian faulting" (line 48) because low-angle thrust faults still form with horizontal sigma1 and so they are Andersonian. It is better to say "fault orientations deviating from orientations considered optimal for basaltic rock masses".

Reviewed Lines 41-43: *"the formation of low-angle faults is inconsistent with Mohr-Coulomb rheology, in which faults nucleate at 30-40° to the major principal stress σ_1 (that is horizontal in contractional regimes in the context of Anderson's theory [8,9])."*

For the map in Figure 2, please state projection and center of projection, so the reader would quickly be able to locate it in their own GIS.
We have added the requested information to Figure 2 in the revised manuscript.

Line 161 when referring to Loveless and Klimczak, 2025, it should be Loveless et al., 2025 (Reference 19).

Reviewed Line 152-154: "*Within this range, dip angles are independently constrained for three of our seven targets: Endeavour Rupes (H02, β = 6°*[17]*), Blossom System (β = 10°*[16]*), and Enterprise Rupes (β = 15° measured*[16]*, with a modelled minimum of 9°*[20]*).*"

Reference 19 is now reference 20.

Review of NCOMMS-25-45873-T
February 5, 2026

The manuscript organization and presentation have been very much improved. Overall, the manuscript is clear, well-illustrated, and quite readable. I maintain my opinion that the experiments are a useful contribution to the community, as is a quantification of the physical properties implied by particular fault dips. Furthermore, I appreciate the detailed responses to my previous comments, which have been largely addressed.

In reading the new manuscript, I have one major new concern regarding the calculation of basal friction. While attempting to verify whether the suggested direct solution of equations 6-8 (see later comment) reproduces the authors' predicted values of μ_b, I found that the equations implemented in the provided code do not match those presented in the manuscript.

Specifically, the code implies:

$$\mu_b = (1+W)\frac{\alpha}{\beta} + W - \frac{S_b}{\rho g H} \tag{1}$$

with

$$W = 2\sin(\phi)(1-\sin(\phi)) + \frac{C}{\rho g H} \tag{2}$$

Using these equations, I am able to reproduce the values for μ_b reported in Extended Data Table 1, using α_{calc} for α to ensure comparability with the authors' calculations.

However, the equations as written in the manuscript lead to:

$$\mu_b = (1+W)\alpha - W\beta - \frac{S_b}{\rho g H} \tag{3}$$

with

$$W = 2\frac{\sin(\phi)}{1-\sin(\phi)} + \frac{C}{\rho g H} \tag{4}$$

I verified that the equations written in the manuscript (from which equations 3-4 are derived algebraically) are consistent with those in Suppe, 2007. I have not performed an exhaustive recalculation, but a few examples demonstrate that basal friction coefficients calculated using these equations can sometimes differ substantially from the presented results:

Profile	β	α	μ_b from Equations 1-2	μ_b from Equations 3-4
P1_ER	5	0.82	0.24	0.28

P1_ER	10	0.79	0.37	0.53
P1_ER	15	0.78	0.41	0.77
P1_AH	5	1.45	0.05	0.32
P1_PC	10	2.58	0.1	0.64
P5_EntR	10	2.91	0.05	0.66
P5_PR	15	0.68	0.42	0.76

It is therefore necessary to ensure that the code is solving the equations presented in the manuscript. Resolving this inconsistency is likely to lead to updated values for μ_b, which may in turn affect the interpretation of the results and the conclusions of the manuscript.

I note that it is to the authors' credit that their effort to make their analyses transparent and reproducible and the clarity of their methodological presentation enabled this assessment.

Additional comments which are mostly minor:

Line 29-30: I suggest replacing "primarily" with ", for example,". There is not a consensus that planetary contraction is driven primarily by secular cooling. For example, substantial contraction could have been driven by inner core crystallization.

Line 31: Byrne et al. estimate something like 4-7 km. Why give a range for the low estimates but not for the high?

Line 33: Would be clearer if "Andersonian fault geometry" were either defined or a paper which gives a definition cited.

Figure 2: much improved, very informative.

Lines 137-143: The way the model is presented is still confusing. The BFIM is a numerical function solver. It should be possible to solve for the μ_b which gives the observed topographic angle with arbitrary accuracy. I don't really understand why there is a distinction between α_{obs} and α_{calc} at all, or why the error quantification is necessary. From looking at the code, perhaps there needs to be a separate discussion of fitting α_{obs}?

Line 155: Is there another way to express the lack of solution other than "The model does not converge": convergence has a specific meaning which I'm not sure is relevant here. "No solution is possible"? "A value for μ_b outside the range considered would be necessary"?

Lines 156-157: This is a nice sentence which adds clarity, though my previous comment remains.

Line 189-190: I don't believe the onset of explosive volcanism is well constrained, since early explosive deposits could easily have been overprinted. Furthermore, given the planet-wide resurfacing which seems to have occurred/ended at 4.1-4.0 Gya (Marchi et al., 2013), an onset time of 3.9 Gya cannot be very firm. I believe the author's argument (that elevated pore pressure due to volcanic degassing is likely not the weakening agent) stands without any timing constraint.

Line 215-219: There are a few specifics here which I do not think are well supported, and removing them may strengthen the authors' points or widen the applicability of their findings. First, is it necessary for the deep graphite-rich layers to be primordial? The primordial graphite layer (the original flotation crust) ought to be buried beneath the 30 or so km of crust inferred from gravity data. But couldn't graphite just as well be sourced from shallower layers emplaced by impacts (that is, the deep graphite brought up in ejecta or melt pool differentiation)? I suggest simply removing the word "primordial". Second, I am still unconvinced that graphite is the *most* plausible weakening agent, but rather that graphite is *a* plausible weakening agent. There are other possible weakening agents (e.g. sulfides) which have not been evaluated – not that they need to be mentioned, but "most" implies they have been evaluated. But the study also establishes how weakening an agent must be, which is a powerful contribution whether or not the agent turns out to be graphite – why limit it? The authors make a strong point that the weakening agents/processes typical for Earth should not be present on Mercury. This inspires the search for Mercury-typical weakening agents such as graphite.

Figure 3: this figure is difficult to read because of its low resolution. I'm confused about the internal coefficient of friction. Is μ_w that of pure basalt? It looks like the figure is communicating that 25% graphite is assumed in the wedge, but the main text implies that graphite is present just in the shear zone and therefore lowering only the basal coefficient of friction.

Line 257: LRM is defined in two places in two ways (material vs. materials). I have most commonly seen material (not plural), but regardless, one should be chosen.

Figure 4: This is a really nice illustration: informative and beautiful.

Line 269-276: This is a very important and well-stated point. Given the inference of ~1 wt.% carbon in Mercury's crust in general, is a greater concentration of graphite at depth (i.e., LRM) even necessary to explain the fault weakening?

Line 290-291: I am a bit confused by this statement. As I understand it, this manuscript does not really go into how low-angle thrust faults accommodate global contraction, but rather gives a plausible weakening mechanism that would allow for the low angles previously observed for thrust faults.

Line 293-296: The authors have presented calculations of low friction coefficients assuming a low dip angle. Does the model predict high friction coefficients at this classic high dip angle?

Line 313: Planetary should not be capitalized.

Line 340: How is the starting point of the transect determined? This seems extremely important for calculating the bulge area and the height. Is there some fitting that needs to be described here?

Line 352: This section is contributing to my confusion about whether μ_w must be lowered or only μ_b.

Line 361-364: It should be possible to achieve $\alpha_{calc} = \alpha_{obs}$ with arbitrarily good precision by decreasing $\Delta\mu_b$. I find it quite confusing for this to be presented as a fit-and-error calculation when it really seems to be simply a numerical solution to a function. In fact, an inversion is not necessary; Equations 6-8 permit an analytical solution for μ_b as a function of alpha as in Equation (3) above, repeated here:

$$\mu_b = (1 + W)\alpha_{obs} - W\beta - \frac{S_b}{\rho g H}$$

Extended Data Table 1: The columns need units.

References

Suppe, J. (2007). Absolute fault and crustal strength from wedge tapers. Geology, 35(12), 1127. https://doi.org/10.1130/G24053A.1

Marchi, S., Chapman, C. R., Fassett, C. I., Head, J. W., Bottke, W. F., & Strom, R. G. (2013). Global resurfacing of Mercury 4.0–4.1 billion years ago by heavy bombardment and volcanism. Nature, 499(7456), 59–61. https://doi.org/10.1038/nature12280
